# Supplementary material for: Genomic adaptations to aquatic and aerial life in mayflies and the origin of insect wings
Source: Nat Commun. 2020 May 26;11:2631. doi: 10.1038/s41467-020-16284-8 (PMC7250882; doi:10.1038/s41467-020-16284-8)
Supplement: Supplementary file 1 — Supplementary Information [file 41467_2020_16284_MOESM1_ESM.pdf]

Supplementary Information

Genomic adaptations to aquatic and aerial life in mayflies and the origin of insect wings

Almudi et al.

Contents:

Supplementary Note 1. Genome sequencing and assembly
- 1.1 Sample collection
- 1.2 Genomic DNA samples of *C. dipterum* for genome-wide sequencing
  - 1.2.1 Illumina sequencing
  - 1.2.2 Nanopore sequencing
- 1.3 Hybrid Assembly Strategy
- 1.4 RNA sequencing
  - 1.3.1 PE RNA samples
  - 1.3.2 SE RNA samples
- 1.5 Gene annotation
- 1.6 Repeat masking and TEs

Supplementary Note 2. Resources for comparative genomics
- 2.1 Gene family reconstruction and orthology assignment

Supplementary Note 3. Comparative transcriptomics
- 3.1 Transcriptomics clustering along life cycle stages (Mfuzz)
- 3.2 Differential gene expression analysis (DESeq2)
- 3.3 Weighted Gene Correlation Network Analysis (WGCNA)

Supplementary Note 4. Chemosensory gene families
- 4.1 Identification and annotation of chemosensory gene complements
  - 4.1.1 Functional and structural classification of CS sequences
- 4.2 Phylogenetic reconstruction
- 4.3 Gene Clustering analysis
- 4.4 Odorant Binding Proteins and Chemosensory Proteins
- 4.5 Gustatory and Odorant Receptors
- 4.6 Ionotropic Receptors
- 4.7 Sensory neuron membrane proteins
- 4.8 OBPs spatial gene expression patterns (in situ hybridizations)
- 4.9 Gene clustering and heatmap plots

Supplementary Note 5. Opsin complement
- 5.1 Identification and annotation of opsin genes
- 5.2 Gene trees
- 5.3 In situ hybridizations

Supplementary Note 6. Phylostratigraphy

Supplementary Note 7. Evolutionary origin of wings
- 7.1 Functional test of shared genes
- 7.2 Tissue specific transcriptomics

Supplementary Figures
Supplementary References

Supplementary Note 1. Genome sequencing and assembly

1.1 Sample collection

The samples were collected from an inbred line of *C. dipterum* kept in the laboratory for 7 generations [1].

1.2 Genomic DNA samples of *C. dipterum* for genome-wide sequencing

1.2.1 Illumina sequencing

DNA was extracted from five individual adult males using a standard Phenol:Chloroform protocol. The average amount of gDNA obtained per individual was 1 μg. The extracted DNA was used to build an Illumina sequencing library with fragment size 450bp. This library was sequenced using TruSeq Rapid SBS Kit v2 (Illumina Inc.), in paired end mode with a read length of 2x250bp and in two sequencing lanes of HiSeq2500 flowcell v2 (Illumina Inc.) according to standard Illumina operation procedures. A total of 160.89 Gb of raw sequence were produced. Primary data analysis, the image analysis, base calling and quality scoring of the run, was processed using the manufacturer's software Real Time Analysis and followed by generation of FASTQ sequence files by CASAVA v1.8. The FastQC reports suggested that quality decays dramatically towards the end of the read, specially affecting some tiles during the last sequencing cycles. Noticeably, the average base quality scores are above 30 (1 error every 1000 base calls) in the first 180 bp of the reads.

1.2.2 Nanopore sequencing

Five other samples, also of adult males from the same inbred line, were utilised to extract High-Molecular-Weight DNA, using MagAttract HMW DNA Kit (Qiagen) following the manufacturer's instructions, and build different library preparations for Oxford Nanopore Technologies (ONT) Sequencing.

The $1D^2$ genomic libraries for the nanopore sequencing were prepared using the Ligation sequencing kit SQK-LSK308 (Oxford Nanopore Technologies, ONT) following the manufacturer's recommendations. Briefly, the adapter mix II was ligated to 2 μg of purified gDNA, followed by 0.4X AMPure XP Beads purification and $1D^2$ adapter ligation. These two ligation steps were performed using the NEB Blunt/TA ligase Master Mix and finally purified with

the 0.4X AMPure XP Beads, washed with ABB buffer (ONT) and eluted with Elution Buffer (ONT).

Five sequencing runs were performed using FLO-MIN107 R9.5 flow cells on the MinION/MKI Pk.1 instrument (ONT). The MinKNOW (v1.10.11) interface QC (ONT) was run in order to assess the flow cell quality and this was followed by the flow cell priming. After the flow cell priming, the pre-sequencing mix was combined with Running Buffer (ONT), water, and Library loading Beads (ONT) and loaded in the flow cell. The sequencing data was collected over 48 hours and the quality parameters of the sequencing runs were further monitored by the MinKNOW platform. The raw data were base-called with Albacore 2.1.0.

The total yield from the five runs was more than 7.56Gb of sequence. The read length distribution was characterized by an N25 of 5.37 Kb, N50 of 3.51 Kb (i.e. 50% of the data contained reads 3.51Kb or longer) and N95 of 1.64 Kb.

1.2.3 PreQC Analyses

Before processing the reads, we estimated the genome size using three different methods. First, an analysis of k-mers present in the sequence reads of the PE450 library was carried out using Jellyfish[2] to count k-mers of length 17. A peak k-mer depth was observed at 722-fold k-mer coverage (Supplementary Figure 1). A rough estimate of genome size can be made by dividing the total number of counted k-mers (150,314,958,593) by the k-mer coverage (722), which gives 208.19 Mb. Second, accounting for sequencing error, bias, and repetitive sequence using the program *gce*[3] with k=17, we obtained a similar estimate of 208.35 Mb. Using a k=31, Maryland Super-Read Celera Assembler (MaSuRCA) v3.2.3 [4,5] provided a slightly lower estimate, 198.9 Mb. Finally, SGA preqc [6], which also uses k=31, gave an estimate of approximately 176.7Mb (Supplementary Data 2).

A detailed look at the 17-mer distribution indicated the presence of at least two contaminant genomes of different molarity in our sample. In order to detect such contaminants before assembling the mayfly genome, we ran Kraken [7] on the raw paired-end reads. This approach allowed us to detect and identify contaminant species in 4.21% of the reads (Supplementary Data 3).

1.2.4 Pre-processing the sequencing data

Due to the base quality distribution and the massive sequencing coverage of the PE library, we decided to trim the reads to 180bp and select those pairs with higher base quality along the read. Therefore, after trimming to 180 bp, we used FASTX Toolkit 0.0.13 (http://hannonlab.cshl.edu/fastx_toolkit/) to keep those reads with a minimum base quality of 35 over at least 97% of their sequence. Finally, only valid pairs were kept for further filtering of contaminants.

Then, all remaining paired reads were filtered by mapping (gem-mapper[8] with up to 2% mismatches) against a contamination database that included phiX, Univec sequences, *E. coli*, the complete mitochondrial genome of *Ephemera orientalis* and the genomes of the contaminants detected with kraken [7] in the PE reads (Supplementary Data 4). These clean PE reads accounted for 55,502,519 2x180bp pairs, representing a sequencing coverage of 95.9x.

The nanopore reads were only filtered against the DNA_CS sequenced (lambda phage fragment spiked in to assess error rates). Matches to the control sequence using the following BWA MEM (v0.7.12)[9] options were filtered out: -k 16 -W 60 -r 10 -A 1 -B 1 -O 1 -E 1 -L 0 -t.

1.3 Hybrid Assembly Strategy

We ran a hybrid assembly using MaSuRCA v3.2.3 [5,10] with 95.9x short-read Illumina and 36.34x ONT long-read coverage. All the options were set to default, but activating the usage of the linking mates and choosing the *Celera* assembler for contigging and scaffolding. The final assembly, *clodip*2, accounted for a total of 180,286,980 bp, with a contig N50 of 434,950 bp and scaffold N50 of 461,411 bp (Supplementary Data 2).

We assessed the assembly quality in terms of total length and gene completeness. The assembly length of 180.28 Mb is within the range of genome size estimates obtained with different k-mer analyses (176.7-208.35 Mb). Gene completeness was estimated with two different programs. CEGMA v2.5 [11] identified 95.56% Complete and 1.21% Fragmented Core Eukaryotic Genes (CEGs) and 3.23% Missing CEGs (This means that only 8 out of 248 CEGs were not identified). BUSCO v3 [12], which used a more comprehensive database (*insecta_odb9*) that includes up to 1,658 BUSCOs, found 96.9% Complete BUSCOs (94.1% in single-copy and 2.8% Duplicated), 1.3%

Fragmented and only 1.8% missing (29 genes, see Supplementary Data 2). In summary, 98.2% of the *insecta_odb9* BUSCOs are present in our *clodip*2 assembly.

1.4 RNA sequencing

A comprehensive collection of 37 RNA-seq datasets of multiple developmental stages, e.g. days post fertilization (dpf), and dissected tissues and organs was generated using the Illumina technology.

Four embryonic stages (E4: 4 dpf or germ disc stage (2 replicas: x2), E6: 6 dpf or segmented embryo (x1), E10: 10 dpf or revolution stage (x2), E14: 14 dpf or pre-nymph (x2)), heads from nymphs at three stages: early (x4), mid (x2) and late nymph (x4), adult male heads (x2), adult female heads (x2), nymphal gills (x2), nymphal wing pads (x2), nymphal gut (x2), nymphal Malpighian tubules (x2), adult muscle (x2), testes (x2), ovaries (x2) and adult brain (x2) samples were obtained from the *C. dipterum* inbred line kept in the laboratory. Organs and embryos were processed immediately after dissection and RNA was extracted using RNeasy Mini Kit (Qiagen) or RNAqueous™-Micro Total RNA Isolation Kit (Ambion) following manufacturers' instructions.

1.4.1 PE RNA samples

Illumina libraries were constructed from total RNA samples of four embryonic stages (x2 E4, E10 and E14; x1 E6), adult male heads (x2), adult female heads (x2), nymphal gills (x2), nymphal wing pads (x2), adult muscle (x2), testes (x2) and ovaries (x2). Total RNA was quantified by Qubit® RNA BR Assay kit (Thermo Fisher Scientific) and the RNA integrity was estimated by using RNA 6000 Nano Bioanalyzer 2100 Assay (Agilent).

The RNASeq libraries were prepared with KAPA Stranded mRNA-Seq Illumina® Platforms Kit (Roche-Kapa Biosystems) following the manufacturer´s recommendations. Briefly, 400-500ng of total RNA was used as the input material, the poly-A fraction was enriched with oligo-dT magnetic beads and the mRNA was fragmented. The strand specificity was achieved during the second strand synthesis performed in the presence of dUTP instead of dTTP. The blunt-ended double-stranded cDNA was 3´adenylated and Illumina TruSeq indexed adapters were ligated. The ligation product was enriched with 15 PCR

cycles and the final library was validated on an Agilent 2100 Bioanalyzer with the DNA 7500 assay.

The libraries were sequenced on a HiSeq 2500 (Illumina, Inc) in paired-end mode with a read length of 2x76bp using TruSeq SBS Kit v4 or Rapid TruSeq v2 following the manufacturer's protocol. Over 45 million paired-end reads for each library were generated in a fraction of a sequencing flow cell lane. Image analysis, base calling and quality scoring of the run were processed using the manufacturer's software Real Time Analysis (RTA 1.18.66.3) and followed by generation of FASTQ sequence files.

In addition, samples of gut (x2), Malpighian tubules (x2) and female brain (x2), were sequenced on an Illumina HiSeq2500 machine generating an average of 50 million strand specific 100-nt paired-end reads.

We detected that one of the Female Head replicates showed an unusual pattern when we performed Principal Component Analysis (PCA) and clustering of the samples: instead of clustering together with Male heads or Female Brains, it was close to some embryonic samples. As an ovoviviparous species, the body cavity of *C. dipterum* adult female individuals is almost entirely full of developing embryos in close contact with the head region. Thus, some embryonic RNA could have been also captured during the extraction of this female head RNA sample. Therefore, we did not use this sample in some of the analyses in which the presence of this head replicate was not essential.

<u>1.4.2 SE RNA samples</u>

RNA obtained from the heads of three nymphal stages was used to generate Illumina libraries through a standard Illumina TruSeq protocol. Samples corresponded to sectioned heads from four young individuals the sex of which was undetermined (early nymphs, resulting in 4 replicates), two heads of female mid nymphs (mid nymphs, 2 replicates) and four late individuals, two females and two males (late nymphs, 4 replicates, as sex was not taken into account for the analyses in which they were used). We obtained an average of 10 million non-strand specific of 50-nt single end reads.

<u>1.5 Gene annotation</u>

Reads from all PE transcriptomes were assembled together using Trinity [13] and subsequently aligned to the genome using the Program to Assemble Spliced Alignments PASA pipeline [14]. After conceptual translation, high-quality transcripts were selected as a training set to build a hidden-Markov profile for *de novo* gene prediction in Augustus [15].

Reads were aligned to the genome using Spliced Transcripts Alignment to a Reference STAR with default parameters [16] and transcriptome assembled using Stringtie for each sample [17]. The assemblies derived from all samples were merged using Taco which ensures high accuracy fusion of transcriptomes [18]. This transcriptome assembly contained 31,913 gene loci,- including protein-coding and non-coding-, and 56,639 transcripts. In parallel, alignments from all samples were merged in order to filter and summarise splice-junctions using the Portcullis tool ([https://github.com/maplesond/portcullis]).

Consensus transcriptome assembly and splice-junctions were converted into hints and provided to the Augustus gene prediction tool which yielded 16,364 evidence-based protein-coding gene models. Isoforms and UTR regions were added by updating Augustus gene models against the PASA transcriptome databased for two subsequent rounds, resulting in 34,699 transcripts for the 16,364 genes. These models contain 4,308 non-redundant PFAM domain models in 11,030 genes as assigned using the PfamScan tool.

Taking the orthology (from Orthofinder [19]) from *C. dipterum* or *Strigamia maritima* to *Drosophila melanogaster* (taking the *D. melanogaster* gene to GO database from Ensembl BIOMART, and pulling across all the GO terms for each gene)*,* we generated a topGO gene to GO key, by copying across all GO terms represented in each orthogroup from *D. melanogaster* to *C. dipterum* or *S. maritima,* respectively. Functionally annotated mayfly (or *S. maritima*) proteome was utilised for subsequent analyses (e. g., Enrichment analysis for Gene Ontology using topGO package (v 2.36.0)[20])

1.6 Repeat masking and TEs

RepeatModeler, a *de novo* repeat identification program, version 1.0.11 [21] was run on the *C. dipterum* genome with default parameters. We identified 65 *de novo* transposable element (TE) sequences in the *C. dipterum* genome. The

consensus sequences reported by RepeatModeler which had not been properly classified (thus assigned to the Unknown class) were filtered using BLASTX from the BLAST+ 2.9.0 release against the nr database (downloaded using the update_blastdb.pl script on the 2019-07-30). Only elements whose hit title contained at least one element from a set of TE-related string patterns ('transpos', 'integras', 'retro', among others) were kept. The consensus sequences were added to the RepBase library [22] for Metazoa from the 2018.10.26 update and this new library was used to mask the genome with RepeatMasker [23] version 4.0.9. The divergence distribution was obtained by calculating the Kimura distance from each TE classified up to the family level to the consensus of its family. The larger number of differences between the identified copy and its consensus translates into a larger Kimura distance. The distribution was plotted (Supplementary Figure 1d) using the helper scripts from RepeatMasker. Unclassified sequences were not included in this plot.

Supplementary Note 2. Resources for comparative genomics

<u>2.1 Gene family reconstruction and orthology assignment</u>

To obtain phylogeny-based orthology relationships between different taxa, the predicted proteomes of 14 species (*Apis mellifera*[24]*, Cloeon dipterum, Daphnia pulex*[25]*, Drosophila melanogaster*[26,27]*, Ephemera danica*[28]*, Folsomia candida*[29]*, Homo sapiens, Ladona fulva*[28]*, Pediculus humanus*[30]*, Stegodyphus mimosarum*[31]*, Strigamia maritima*[32]*, Tigriopus californicus*[33]*, Tribolium castaneum*[34]*, Zootermopsis nevadensis*[35]) representing major arthropod lineages and outgroups were used as input for OrthoFinder 2 [19]. All versus all similarity searches were obtained using DIAMOND (0.9.15)[36] and an inflation parameter of 1.5 for the clustering. After this step, OrthoFinder 2 identified orthogroups, which are groups of proteins that share significant homology.

Then, proteins belonging to each orthogroup were aligned using MAFFT(v7.221)[37] (LINSI mode), trimmed with trimAl (v1.4)[38] (-gt 0.2), and RAxML-NG [39] was used to build the maximum likelihood phylogenetic tree (--model LG+G4 --seed 12345). The resulting trees were parsed with the OrthoFinder 2 [19] pipeline to discriminate between orthologues and paralogues within each orthogroup.

Gene gains and losses in major transition of arthropod evolution were inferred with a recently presented pipeline [40-42]. Similarity searches of protein coding genes were performed using DIAMOND (0.9.25)[36]. Clustering was performed with MCL v14-137 with a granularity parameter of 2 [43]. The patterns of gains and losses were reconstructed based on the taxonomic occupancy of the gene clusters. Briefly, novel genes are gene families present in at least one species of each of the two main lineages of clade (e.g., for Hexapoda, present in at least one paleopteran and one pterygotan) and absent outside the clade of interest. Core novel genes are genes present in every single lineage of a clade or absent only once, and are absent in taxa outside the clade of interest. (Supplementary Figure 1). The *C. dipterum* GO annotations were used to inspect the functions of these genes in Paleoptera; for the rest of the nodes, the functions of the genes were inferred by submitting *D. melanogaster* IDs to PantherGO [44].

Supplementary Note 3. Comparative transcriptomics

3.1 Transcriptomics clustering along life cycle stages (Mfuzz)

The Mfuzz software [45] was used to perform soft clustering of genes according to developmental and life history expression dynamics in *C. dipterum.* We selected eight developmental and post-embryonic stages: E.4 (4 dpf embryos or germ disc stage), E.6 (6 dpf embryos or segmented embryo), E.10 (revolution stage embryos), E.14 (pre-nymph), early nymph heads (2.5 mm nymphs), mid nymph heads (4 mm nymphs), late nymph heads (5 mm nymphs) and adult heads. We mapped RNA samples to the annotated genome using hisat2 v2.1.0 [46]. Then, we obtained counted reads for each of the samples (two replicates each condition, except for the E.6 sample, with only one replicate) with samtools utilities (<http://www.htslib.org/>) and htseq-count [47]. To be able to profile the eight RNA-seq datasets together (PE and SE libraries), we used "R1" datasets from the PE libraries. Datasets used were an average of the replicates for each time point to avoid experimental or sex bias. Datasets were normalised using "DESeq2" library and genes with low variability (variance < 3) across the datasets were removed (1475 genes filtered out) for the analysis. Additional pre-processing was performed: We filtered out genes with missing values (filter.NA(minimalSet, thres=0.5) and fill.NA(eset, mode="knnw")) and genes

whose standard deviation was 0 (filter.std(eset, min.std=0)). Finally, we standardised the samples using 'standardise' function from Mfuzz package. Direct estimate of the fuzzifier 'm' parameter was calculated using 'mestimate' function and resulted m=1.43. Minimum centroid distance was calculated using "Dmin" function to obtain the optimum number of clusters, which resulted in 30 (Supplementary Figure 2). 'Dmin' function calculates the minimum centroid distance as the minimum distance between two cluster centres produced by the c-means clusterings. We used the decrease of minimum centroid distances plotted versus a range of cluster number and a slower decrease of the minimum centroid distance for higher cluster number to determine the number of clusters. However, we assessed the suitability of 30 as the number of clusters by checking GO term enrichments obtained using topGO package [20].

Mfuzz soft clustering allows genes to be included in several clusters, giving each gene in each of the cluster 'Memberships' values. In other words, genes were assigned to clusters by highest scores of membership. Membership values therefore represent the correlation of expression of genes with the cluster, and are colour coded in Mfuzz plots with high values (high correlation) in magenta and low values (poor correlation) in greens (Supplementary Figure 2, Supplementary Data 9). Moreover, we determined the core genes of each of the clusters that represent genes whose membership value was higher than the alpha core that we set as 0.7, as suggested by the developers (Supplementary Data 9). We also determined that the overlap between core genes in each of the cluster was zero.

To assess the stability of the clusters representing the life history traits of the mayfly (cl 21, cl 18 and cl 30), we performed Mfuzz analyses varying 'm' parameter values to m=1.53 (which produced less robust clusters) and m= 1.33 (which generated 'hard' clustering, with genes whose Membership value was close to 1 and 0) and observed that these clusters maintained their internal structure, a high overlap of the genes contained in them and the GO term enrichment analyses showed very similar categories (Supplementary Figure 7).

Taking the functional annotation of *C. dipterum,* (see methods 1.5 annotation) we generated a topGO gene to GO key. We performed an Enrichment Analysis for Gene Ontology using topGO package (v 2.36.0) [20]

and used all genes considered in the Mfuzz experiment as a background. Uncorrected *P* values shown in plots corresponded to two-sided Fisher's exact tests as provided by topGO [20]. These analyses allowed us to assign GO terms that reflected main biological and molecular processes occurring for each of the clusters. For instance, cluster 2, 3, 8 or 21, which included genes with high expression during embryonic stages, showed enrichment in terms such RNA splicing, epithelium development, animal organ morphogenesis, etc. associated to morphogenetic processes happening at these developmental points. By contrast, clusters whose genes are expressed during nymphal stages, such as cluster 1, cluster 7, cluster 9 or cluster 18 exhibited enrichment in categories related to chitin, cuticle formation and perception of chemical stimulus (Supplementary Figure 2, Supplementary Data 9).

3.2 Differential gene expression analysis (DESeq2)

We mapped adult male and adult female head RNA samples to the annotated genome using hisat2 v2.1.0 [46]. Then, we obtained counted reads for each of the samples (two replicates each condition) with samtools utilities (<http://www.htslib.org/>) and htseq-count [47]. Differentially gene expression analysis was performed with default parameters of "DESeq2 v1.20.0" package in R [48] to detect whether there were differences in gene expression based on sex.

We obtained 3,243 genes that were differentially expressed between male and female heads (padj < 0.05), and of them, 1,406 genes were upregulated in female heads and 1,837 were upregulated in male heads (Supplementary Data 12).

3.3 Weighted Gene Correlation Network Analysis (WGCNA)

We used the cRPKM metric (corrected-for-mappability Reads Per Kilobasepair of uniquely mappable positions per Million mapped reads [49]) to perform these gene expression analyses. We selected the transcript with the largest number of exons for each of the protein-coding genes annotated in the *C. dipterum* genome. In the case of *D. melanogaster* and *S. maritima* we used 19 and 14 datasets from [50] (Supplementary Data 13) and calculated cRPKMs as described in [49]. Note that the recommended number of samples to perform WGCNA

analysis is 15 and we only used 14 samples for *S. maritima*. However, when analysing the modules obtained for this centipede species, we were able to assign different 'categories' based on either high gene expression in a main tissue and/or GO term enrichment to different modules representing meaningful biological processes or functions.

To characterise modules of co-expressed genes across our RNAseq datasets that include developmental stages and nymphal and adult tissues and organs, we utilised WGCNA [51]. We selected 26, 19 and 14 samples of *C. dipterum*, *D. melanogaster* and *S. maritima* (Supplementary Data 13). We performed the analyses using as datasets all the genes that showed variance across samples (coef. var ≥ 1). In total, the number of genes included in each dataset were, 13,720 for *C. dipterum,* 12,463 for *D. melanogaster* and 13,001 for *S. maritima* (Supplementary Data 13). To construct the weighted gene network (co-expression similarity), we used softpower (BETA) settings of 6 for *C. dipterum, D. melanogaster* and *S. maritima* datasets. Calculated 'adjacency' was utilised to construct the Topological Overlap Matrices (TOM) and their corresponding dissimilarities. After performing the hierarchical clustering with the 'hclust' function, we set a minimum module size of 30. Finally, we merged modules with similar expression profiles using as height cut 0.25 to obtain 22, 17 and 21 modules for *C. dipterum, D. melanogaster* and *S. maritima,* respectively. These modules contained genes that were assigned to them based in their membership values across the RNA samples used in the study. Each module was designated with a tissue or a biological category related to gene expression across tissues and GO enrichment (Supplementary Data 13 and Supplementary Data 1). To assess the robustness of the modules generated we took *C. dipterum* samples and performed replications of the analysis. We modified the default parameters used (BETA 6, VAR 1) to BETA 12, VAR 1; BETA 18, VAR 1; BETA 6, VAR 5 and BETA 6, VAR 20 and checked whether the gene content of the modules was modified. In all the replications performed most of the genes were maintained in their assigned modules (Supplementary Data 13). Finally, we analysed the overlap between orthologous groups for each pair of modules for each of the species in a pairwise manner.

To evaluate the significance, we performed hypergeometric tests. We translated the list of genes contained in each module into a list of 'orthologous gene families' (obtained from our orthologous families from OrthoFinder2 [19]). This way, two genes that belonged to the same family were both translated to their family's identifier. This allowed us to compare modules across species.

After this 'translation', we kept a set of unique gene families per module and then considered a hypergeometric test where the number of unique gene families (ugf) in one module was the sample size (SS), the number of ugf of the other module was the 'success in population' (SIP), their common ugf was the 'success in sample' (SIS) and the total available ugf (including those found in neither of the two modules tested) was considered the population (POP). In other words, we computed a probability of finding SIS, or more, common families in our sample of SS families (the one module), that also belonged to the other module which contained SIP families out of POP total available families.

```
a = set([module 'a' gene-family-ids])
b = set([module 'b' gene-family-ids])

gene_SS = len(a) # Sample Size
gene_SIP = len(b) # Success in Population
gene_SIS = len(a.intersection(b)) # success in sample
gene_POP = total number of gene family unique ids

hsv = scipy.stats.hypergeom.sf(gene_SIS-1,  gene_POP,  gene_SS, gene_SIP)

value_in_heatmap = -log(hsv, ,10)
```

Having obtained these values for each pair-wise comparison, we visualized them in a clustered heatmap. For this we used the clustermap method of the seaborn python library with the default parameters; hierarchical clustering with euclidean metric and average method (Unweighted average linkage clustering, or UPGMA). In order to stop the few extreme values from dominating the clustering (a couple of module comparisons reach a -log(pvalue) of 100 when most of the other significant comparisons are under 10), we clipped the values at 10 before clustering. Additionally, we set a minimum value of 3 for the visualization of the heatmap, therefore, the comparisons with pval>0.001 were not shown.

To further investigate the module preservation between species, we ran a specific WGCNA module preservation protocol, following the tutorial at "https://horvath.genetics.ucla.edu/html/CoexpressionNetwork/ModulePreservation/Tutorials/HumanChimp.pdf". To do this, we took the two WGCNA results for mayfly and *Drosophila*, then filtered the two sets to include genes that had a single orthologous gene between the two species only (n= 5,247 genes). Next, we renamed the *Drosophila* genes to their *C. dipterum* gene ID equivalent, and ran the module preservation (WGCNA function) and in-group proportion statistics (through the clusterRepro package, 10,000 permutations) to assess module conservation (Supplementary Figure 6b).

Supplementary Note 4. Chemosensory gene families

4.1 Identification and annotation of chemosensory gene complements

We created a dataset containing reference sequences for each chemosensory (CS) gene family (Gustatory Receptors (GR), Odorant Receptors (OR), Ionotropic and ionotropic glutamate receptors (IR/iGluR), CD36/Sensory neuron membrane proteins (CD36/SNMP), Odorant Binding Proteins (OBP) and Chemosensory Proteins (CSP)) from relative annotated insect genomes (Supplementary Figure 3[52-61] and Supplementary Data 10). In addition, we constructed specific Hidden Markov Models (HMM) profiles for each CS gene family based on their Pfam profiles (see Supplementary table 1 in [62]).

We used the sequence database and the HMM profiles in the program BITACORA [63] to (i) identify new CS members, or to curate the already annotated ones, among the pre-compiled gene models of these families (obtained with automatic methods), and (ii) to generate new models (of previously undetected copies) from the genomic sequences. Briefly, we performed various iterative rounds of BLASTP and HMMER searches against the automatically annotated proteins of *C. dipterum* and curated incorrect and incomplete (when possible) gene models. Also, we used TBLASTN against the genomic sequence to identify novel (not annotated by the automatic methods) regions encoding CS proteins. We generated a GTF file containing our curated annotation of *C. dipterum* CS genes.

4.1.1 Functional and structural classification of CS sequences

We classified the novel sequences in different categories based on structural and functional criteria [64]. First, we examined the presence of premature stop codons; these features could represent real non-functional copies (pseudogenes), errors in sequencing or genome assembly steps or inaccuracies in our automatic annotation step based on TBLASTN hits. All sequences encoding complete proteins (CPs) that were free of stop codons were included in the first category (CP set). Operationally, we considered a CP when its length was >80% of the corresponding average protein domain length. In addition, for the GR and OR families, we also required that the CP members contained a minimum of 5 of the 7 transmembrane domains (defined by the software TMHMM version 2.0c [65]; Phobius version 1.01[66]). For the CP IR/iGluR members, we required the presence of the ligand channel domains, namely, PF00060 (ligand-gated ion channel), present in all IR/iGluR subfamilies, i.e., kainate, AMPA, NMDA, conserved IRs (IR25a/IR8a), and divergent IRs [67]. The remaining sequences that were free of stop codons and did not pass the length filter criteria were classified as incomplete proteins (IP set). Finally, the CP and IP sequences exhibiting some in-frame stop codons (that could represent pseudogenes, among other features; Ψ) were incorporated into the Ψ data set. We renamed all proteins with the initials of the family name and a number starting from 100, except those with clear homology to *D. melanogaster* CS members which were named as in *Drosophila*. We added a “p” to the name of incomplete proteins to identify them.

We identified a total of 367 putative proteins across all CS gene families in the genome of *C. dipterum* (see Supplementary Data 10). CS gene families are characterized as fast evolving and highly divergent both across insects, therefore surprisingly, the 95.4% of the copies were already predicted in the automatic annotation, although some of them had incorrect gene models (i.e. extra exons badly assigned to the gene, missing exons…), while the remaining were identified from the genome sequence. The use of deep transcriptome libraries from several stages and tissues in genome-wide automated gene modelling provided these gene models for most of the CS sequences.

4.2 Phylogenetic reconstruction

We included in the phylogenetic analyses all CS genes identified in *C. dipterum*, the members of these families in the fruit fly *D. melanogaster*, and the GR, OR, IR and OBP proteins annotated in the *C. splendens* genome (Ioannidis, Simao et al. 2017). We also included the ORs annotated in *E. danica* genome [61] and the OBPs obtained from a preliminary sequence similarity-based search in *E. danica* genome assembly ([https://www.ncbi.nlm.nih.gov/assembly/GCA_000507165.2/]). The analysis of the OBPs and CSPs was performed with the mature proteins (after excluding the signal peptide identified using SignalP software; [68]). We used MAFFT (with '--auto' option) to build family-specific multiple sequence alignments (MSA) [69]. The phylogenetic analysis was performed with IQ-TREE version 1.6.5, estimating automatically the protein substitution model for each tree [70]. Node support was estimated from 1000 ultrafast bootstrap replicates [71]. The phylogenetic tree images were created using the iTOL webserver [72]. Trees were rooted using outgroups according to available phylogenetic information. Additionally, we used OrthoMCL (v2.0.9; [73]) to infer orthology among the gene family members to compare with the relationships observed in the phylogeny.

4.3 Gene Clustering Analysis

We used computer simulations to test whether the members of a CS gene family are physically clustered in the genome of *C. dipterum*. We assessed the clustering pattern of all annotated CS genes by computing the variance of the number of copies of a given family per scaffold (in 1,395 scaffolds). To build the null hypothesis of no clustering, we randomly chose from the total genome gene set a number of genes equal to the number of copies of the gene family we wanted to test, and calculated the variance of the number of genes per scaffold. We repeated this procedure 10,000 times until having an empirical distribution of this quantity based on all genes. The empirical *P*-value of being distributed across the genome just like any other gene, regardless of being a member of a CS family, was obtained by comparing the observed variance of CS genes per scaffold against this genome-wide distribution using the Empirical Cumulative Distribution Function (ecdf) in R (R Core Team 2016).

4.4 Odorant Binding Proteins and Chemosensory Proteins

We found the largest OBP repertoire known in insects, with a total of 191 fragments encoding members of this family, being 167 of them complete and harbouring the typical conserved cysteine pattern. Among the 24 partial OBP sequences, two are putative pseudogenes (sequences containing premature stop codons that truncate the sequence) and 22 incomplete OBPs that could be either also pseudogenized sequences or just incomplete annotations or assemblies. The *C. dipterum* OBP gene repertoire far exceeds the 109 OBP genes identified in the cockroach *Blatella germanica* [74], and contrast greatly with the four OBPs found in the genome of *Calopteryx splendens* [56], and the 39 putative genes of this family found in a preliminary search of the *E. danica* genome.

We also identified 16 CSP proteins with complete gene models and the typical four cysteine pattern of this family. The phylogenetic tree shows that this genome encodes three highly divergent members of this family (CdipCSP107, CdipCSP108 and CdipCSP113; Supplementary Figure 3b). We could not establish any orthology relationship between *D. melanogaster* and *C. dipterum* CSPs.

We determined that the members of the OBP family of *C. dipterum* are significantly clustered in the genome (*P*-value < 0.0001). This clustering pattern would be expected if new copies originate by tandem gene duplications and their genomic structure is maintained for long time periods by selection against rearrangements, as reported for this family in different insect species [75-77]. An alternative, not selective, explanation is a very recent burst of gene duplication in the lineage of *C. dipterum* (in the family or in the entire genome) that generated large tandem arrays of genes that still remain physically close in the genome. We observed several species-specific phylogenetic clades of these physically close copies in the OBP family tree with relatively short branch lengths, suggesting a very recent origin and, therefore supporting the second hypothesis; nevertheless, we also observe some basal lineages that group with *D. melanogaster* and *C. splendens* sequences, pointing to a much older origin and, therefore, suggesting some selective constraint against cluster rearrangements.

The OBP tree also highlights the presence in *C. dipterum* of an ortholog of the DmelOBP73a protein, here named as CdipOBP73a, which is also present in *C. splendens*, CsplOBP1. This orthogroup is highly conserved across insects, indicating a critical (but already unknown) function in this group [55]. Additional orthologies, such as that of the OBP43a group, were also detected, although bootstrap support was low.

4.5 Gustatory and Odorant Receptors

The genome annotation of chemoreceptors was more challenging as they are 4-fold longer than OBPs and CSPs and their expression is more restricted (most of them are specific to chemosensory appendages). We found that almost half of the sequences encoding chemoreceptors are partial fragments, including also some putative pseudogenes. Nonetheless, we identified 64 GR genes which would encode, at least, 56 receptors, 26 of them being complete. Regarding ORs, we found 50 sequences encoding at least 43 proteins, 29 with full-length models. Interestingly, and unlike most insects, in *C. dipterum* the number of OR and OBP proteins, both involved in olfaction, is very different, supporting the idea that OBPs could have other unknown functions.

The phylogenetic analysis of chemoreceptors uncovered the presence in *C. dipterum* of the highly conserved OR co-receptor, known as ORCO and of 49 specific OR (Supplementary Figure 3c and f). Although many OR copies in Ephemeroptera would have originated in this lineage, some of them would be already present in the common ancestor of *C. dipterum* and *E. danica*.

For the GR genes, we found a gene similar to the *Drosophila* receptors for sugar taste (here CdipGR124), a group of proteins conserved in all insects and also found in the crustacean *D. pulex* [78]. Two additional GR found in this study, CdipGR133 and CdipGR156, are similar to the GR characterized as the CO_2 receptors in *Drosophila*. Apart from these highly conserved sequences, we identified some phylogenetic-based candidates to be the fructose receptor or to participate in bitter compound detection, although the key nodes were largely unsupported. The remaining GRs grouped in species-specific clades and their functions are completely unknown. Noticeably several of these GRs are expressed in the pre-nymphal embryo and in nymphal heads, so these receptors probably also mediate gustation in *C. dipterum*.

4.6 Ionotropic Receptors

Of the 34 (23 and 11) IR/iGluR genes identified in *C. dipterum* (26 complete sequences), 11 are canonical iGluRs, while 23 likely encode ionotropic (putative chemosensory) receptors (Supplementary Figure 3). Particularly, we identified some IR members known to be conserved across insects, such as the co-receptor IR8a, which is duplicated in the mayfly (CdipIR8a_1 and CdipIR8a_2), IR25a and IR76b,IR93a, IR21a, IR40a, IR68a, the three first associated with thermo- and hygrosensory mechanisms [79]. Among the IR members identified in *C. splendens*, only IR75c, which respond to various acids (Prieto-Godino, Rytz et al. 2017) seems to be missing in *C. dipterum*. The phylogenetic analysis also uncovers a small burst of ancient CdipIRs similar in number to the one observed in *C. splendens* (Supplementary Figure 3e). These divergent IRs could be implicated in chemosensory functions but also in the response to other stimuli, such as temperature or humidity, as has been documented in *D. melanogaster* [79]. The diversity of tissues in which these receptors are expressed predicts their broad range of functions. We observed particular IR copies highly expressed in adult heads (IR40a and IR93a), gills (IR68a, IR107, IR8a_1, IR103 and IR114p) and early embryos (E4; IR109, IR112p, IR25a), while canonical iGluRs were found majorly expressed in whole heads and brains.

4.7 Sensory neuron membrane proteins

We identified 12 complete genes encoding CD36-SNMP proteins, and no evidence of partial fragments or pseudogenes in this family. Only SNMP, found to be expressed in specific *Drosophila* pheromone-responding sensory neurons, has been related with chemoreception, playing a key role in the sensory perception of an important fruit fly pheromone by facilitating the contact between the ligand and the membrane receptor [80]. The phylogenetic analysis indicated that the only copy of this family conserved in *C. dipterum* is SNMP2, which is represented by five divergent paralogs. Interestingly, four of these five genes are mostly expressed during embryonic stages, in particular at the segmented embryo stage (E6).

4.8 OBP spatial gene expression patterns (in situ hybridizations)

We selected some of the OBP genes that showed high expression in the gills RNA-seq datasets to investigate the spatial expression pattern within this organ. We designed and generated RNA probes against *OBP199, OBP260* and *OBP219* (Supplementary Data 17). After whole trunks were fixed in FA 4% at 4º C o.n., gills were separated from the body wall and gill's cuticle was carefully removed. Gills were incubated at 60º C with RNA probes after post-fixation in Methanol and Proteinase K treatment. Anti-digoxinenin-AP (*OBP260*, for 1 h 30 min; Roche) or anti-digoxinenin-POD (*OBP199, OBP219,* o.n. at 4º C; Roche) antibodies were used to detect our DIG-labelled probes. *OBP199* and *OBP219* gills were incubated with Tyramide Signal Amplification (TSA) 1:100 in borate buffer for 1 hour. After washing the TSA reaction, gills were stained with DAPI 1:10000 and Goat anti-HRP-Cy3 1:100 (Jackson Immunoresearch) at 4º C, o.n. Image acquisition was done with a Leica SPE confocal microscope. Images were processed with Fiji [81]. For *OBP260* in situ hybridization standard protocol was followed, NBT/BCIP was used as chromogenic reagent.

4.9 Gene clustering and heatmap plots

To generate heat maps representing gene expression of chemosensory gene families throughout multiple samples, we used previous normalised counts (see Mfuzz normalisation using DESeq2 library in 3.1 section: Transcriptomics clustering along life cycle stages (Mfuzz)) and applied the function to the datasets:

```
cal_z_score <- function(x){
  (x - mean(x)) / sd(x)
}
data_subset_norm <- t(apply(Chemos_matrix, 1, cal_z_score))
```

Finally, pheatmap package (v 1.0.12) was used to generate the heat maps through the 'pheatmap' function.

To perform the clustering of tissue transcriptomes (Figure 4g) we first selected the most variable genes by requiring: (i) a minimum coefficient of variation across the 26 samples of 3, (ii) a minimum expression of at least 10 (using the cRPKM metric [49]), and (iii) the expression was supported by at least

100 reads in at least one sample. These cut-offs resulted in a selection of 812 genes, whose log converted cRPKM values were used to perform a clustering in R using (1 - Spearman correlation coefficients) as distance (hclust(as.dist(1-cor(matrix,method="spearman",use ="na.or.complete")))). Heatmap of pairwise correlations was plotted using the heatmap.2 function in R.

Supplementary Note 5. Opsin complement

5.1 Identification and annotation of opsin genes

A total 1247 opsins from all the major metazoans groups were used as seed in the BLAST searches of the predicted protein sequences of *C. dipterum, E. danica* (Edan_2.0;[https://www.ncbi.nlm.nih.gov/assembly/GCA_000507165.2/]) *and L. fulva* (Lful_2.0; [https://www.ncbi.nlm.nih.gov/assembly/GCA_000376725.2/]). All the genes with e-value < 10^{-10} were retained for further analysis. To be considered an opsin it was required to have the retinal binding domain or that the first BLAST against Uniprot was an opsin (for short sequences). These sequences were then merged with those from [82-84]. To this set of sequences, additional mayfly LWS, UV and Blue opsin sequences from transcriptome assemblies for *Baetis sp. EP001* and *Epeorus sp. EP006* were obtained from [83,84] and for *Baetis sp. AD2013* from [85]. Sequences derived from the previously mentioned transcriptomes that were shorter than 100 amino acids were not included in subsequent analyses. We also searched a *Baetis rhodani* shotgun whole genome assembly [86]. The fragmentary nature of this assembly precluded confident annotation of opsin genes, but allowed us to find one candidate exon of a putative ortholog of *C. dipterum* UV-Ops1, that was too short to be included in the phylogenetic analyses (Supplementary Figure 5, Supplementary Data 18). To this dataset, we finally added the sequences for an additional 27 species covering seven orders retrieved from [87].

All mayfly LWS, UV and Blue opsin sequences were carefully inspected and curated to detect gene annotation errors such as fusions and fissions of gene models, missing or spurious exons. Furthermore, in the case of *E. danica*, we detected and corrected several cases of identical sequences derived from haplotypes of the same gene. These errors were corrected using available transcriptomic data and taking into account sequence and intron position

conservation with opsin genes from other species and other opsin paralogs within the same species (Supplementary Data 18). Cd-hit was used to remove the redundant sequences resulting in 687 insect opsins sequences. The alignment was performed using MAFFT[37] with default parameters and regions with more than 70% of gaps were removed using TRIMAL[88]. Phylogenetic reconstruction was performed using Ultrafast bootstrap with 1000 replicates, aLRT Bootstrap and aBayes [89,90] using IQ-Tree-1.6.7 [91] under LG+G4+F model. Furthermore, we produced a reduced version of the dataset where most lineage specific duplicates from Odonata were not included, since these proteins were not relevant for our study and their exclusion significantly reduced the computational cost of the analysis. This data encompassed 359 sequences and was subject to a Bayesian phylogenetic reconstruction using Phylobayes MPI under GTR-G4 model and iqtree-1.6.7 under LG+G4+F [92,93]. In all the phylogenetic analyses the trees were rooted using the melatonin receptor which represent the opsin closest outgroup [82].

5.2 Uv-Ops *in situ* hybridizations

Specific primers were designed to generate DIG-labelled probes against *UV-Ops2* and *UV-Ops4* (Supplementary Data 17). After o.n. fixation of the heads in FA 4% at 4º C, dissected retinas were bleached using Formamide Solution [94] for 16 hours. The following steps were as described for *OBP219* and *OBP199* genes. Briefly, we post-fixated the retinas and treated them with Proteinase K for 15 min. The hybridization was carried out at 60º C o.n. The next day, retinas were incubated with anti-digoxigenin-POD at 4º C o.n. Finally, retinas were incubated with 1:100 TSA in borate buffer. Leica SPE confocal microscope was used to acquire images that were processed with Fiji [81].

Supplementary Note 6. Phylostratigraphy

To classify genes by origin (phylostratigraphy) we used an expanded dataset of 28 species (*Acanthoscurria geniculata*[31], *Apis mellifera*[24], *Caenorhabditis elegans*[95], *Cloeon dipterum, Daphnia pulex*[25], *Drosophila melanogaster*[26,27], *Ephemera danica*[28], *Heliconius melpomene*[96], *Holacanthella duospinosa*[97], *Homo sapiens, Ixodes scapularis*[98], *Ladona fulva*[28], *Laodelphax striatellus*[99], *Lepidurus arcticus*[100], *Limulus polyphemus*[101], *Lingula anatina*[102], *Locusta*

migratoria[103], *Nematostella vectensis*[104], *Parhyale hawaiensis*[105], *Pediculus humanus*[30], *Penaeus vannamei*[106], *Ramazzottius varieornatus*[107], *Sinella curviseta*[108], *Stegodyphus mimosarum*[31], *Strigamia maritima*[32], *Tigriopus californicus*[33], *Tribolium castaneum*[34], *Zootermopsis nevadensis*[35]) aiming to sample more densely arthropod lineages and key taxonomic clades. OrthoFinder2 [109] with DIAMOND [36] as search engine and an inflation parameter of 2 were used to compute orthogroups. A higher inflation parameter was required to increase granularity aiming at capturing more defined orthogroups. Then, the orthogroups with sequences from *Cloeon* and *Drosophila* were selected, and each gene for each species was assigned an age based on the furthest species that had a member of the same orthogroup (e.g. present in *Cloeon* and *Nematostella* -> phylostratum Planulozoa). This resulted in 13 phylostrata for *Drosophila* and 13 for *Cloeon* (see Supplementary Data 6). To compute enrichment tests on gene age, the proportion of gene ages/phylostrata in a subset of interest (e.g. genes in WGCNA module “wings”) was compared to the background proportion of ages of the species of interest using a Fisher’s exact test fisher.test (alternative = "two.sided") in R. The list of p-values for each phylostratum were corrected for multiple-testing using p.adjust(method = "BH") in R, and only those enrichments with a adjusted p-value < 0.01 were classified as significant.

Supplementary Note 7. Evolutionary origin of wings

7.1 Functional test of shared genes

We selected eight genes of the 130 shared orthologs between the wing disc and wing pad modules based on the following criteria: availability of RNAi line in a stock centre, lack of off-target effects of such line, reported undetermined function in the wing in Flybase and presence of orthologs in other species to focus on genes that appeared near the origin of pterygote insects (absent in non-winged insects and present in winged insects or genes that arose by gene duplications at the base of pterygotes). Vienna Drosophila Research Centre (VDRC) lines (see Supplementary Data 15) were crossed to *yw; nub-Gal4;* + line to expressed the RNAi constructs specifically in the wing. Crosses were kept at 25º C for 48 hours and then switched to 29º C to maximize GAL4-

induced RNAi expression. Wings were dissected from adult females and mounted in Hoyer's/Lactic medium for image capture.

7.2 Tissue specific transcriptomics

To characterise similarities between tissue-specific transcriptomes, we calculated which genes where expressed in wing pads and one of the other tissues preferentially, according to cRPKM (Minimum fraction of the minimum expression of test group that has to separate both distributions; ($min_test >= $min_expr && ($min_test-$max_neg)/$min_test >= $min_fr)). We considered that the minimum expression (min_expr) of the test group was 20 and that the difference between the test group (wing pad and second tissue, min_fr) with the rest of the tissues was at least of 30 % (Supplementary Figure 6e, f). To assess the statistical significance, we randomized the 98 counts among the 11 tissues and scored how many times one tissue had a similar differential with the second highest sample comparable to what we observed for gills (i.e. 20 genes more than 10dpf embryos) and/or reached >=42 counts in 1,000,000 iterations. We did not observe any of these two conditions in the 1,000,000 iterations and we therefore propose a p<=10^-6. We also tested other tissues as 'main tissue' and obtained meaningful biological relationships. For instance, when Malpighian Tubules was the main tissue, 125 genes had the Gut as 'second tissue' out of 161 genes with high expression in Malpighian tubules and viceversa, when gut was chosen as 'main tissue' Malpighian tubule appeared as ´second tissue' with 125 out of 181 genes highly expressed in the Gut. In the same manner, 'Gills' second tissue was 'Wing pad' (42 genes) or 'Male head' second tissue was 'Brain' (32). This analysis resulted in 98 genes with wing pad-specific expression and from those, in 42 of the cases, the second tissue was the gills (Supplementary Data 16). We found that 30 of these 42 genes had *Drosophila* orthologs and from those, 40% (12/30) had a known role in wing development.

Supplementary Figures

Supplementary Figure 1

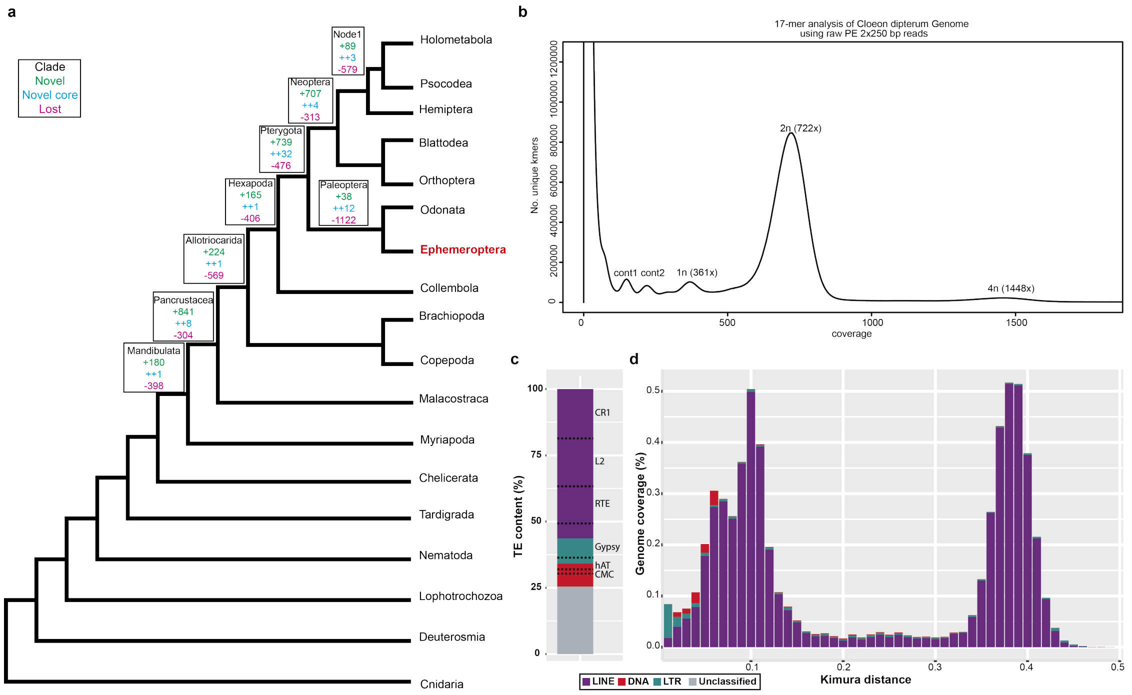

Supplementary Figure 1. **The genome of *C. dipterum*. a,** Simplified animal phylogeny showing number of gene gains and losses for specific lineages. In green, novel genes (those that are present in the common ancestor of the clade but not found in the outgroup), in blue, novel core genes (present in every representative species) and in red, lost genes in the last common ancestor of the clade. **b,** 17-mers analysis of the genome. A peak k-mer depth was observed at 722-fold k-mer coverage. A detailed look at the 17-mer distribution indicates the presence of at least two contaminant genomes (cont1 and cont2) of different molarity in our sample. **c**, TE content by order. The most abundant families in each order are indicated. **d,** TE divergence distribution for the TE copies that were classified at the family level (n=15,070 individual TE copies annotated using the 65 predicted consensus TEs).

Supplementary Figure 2

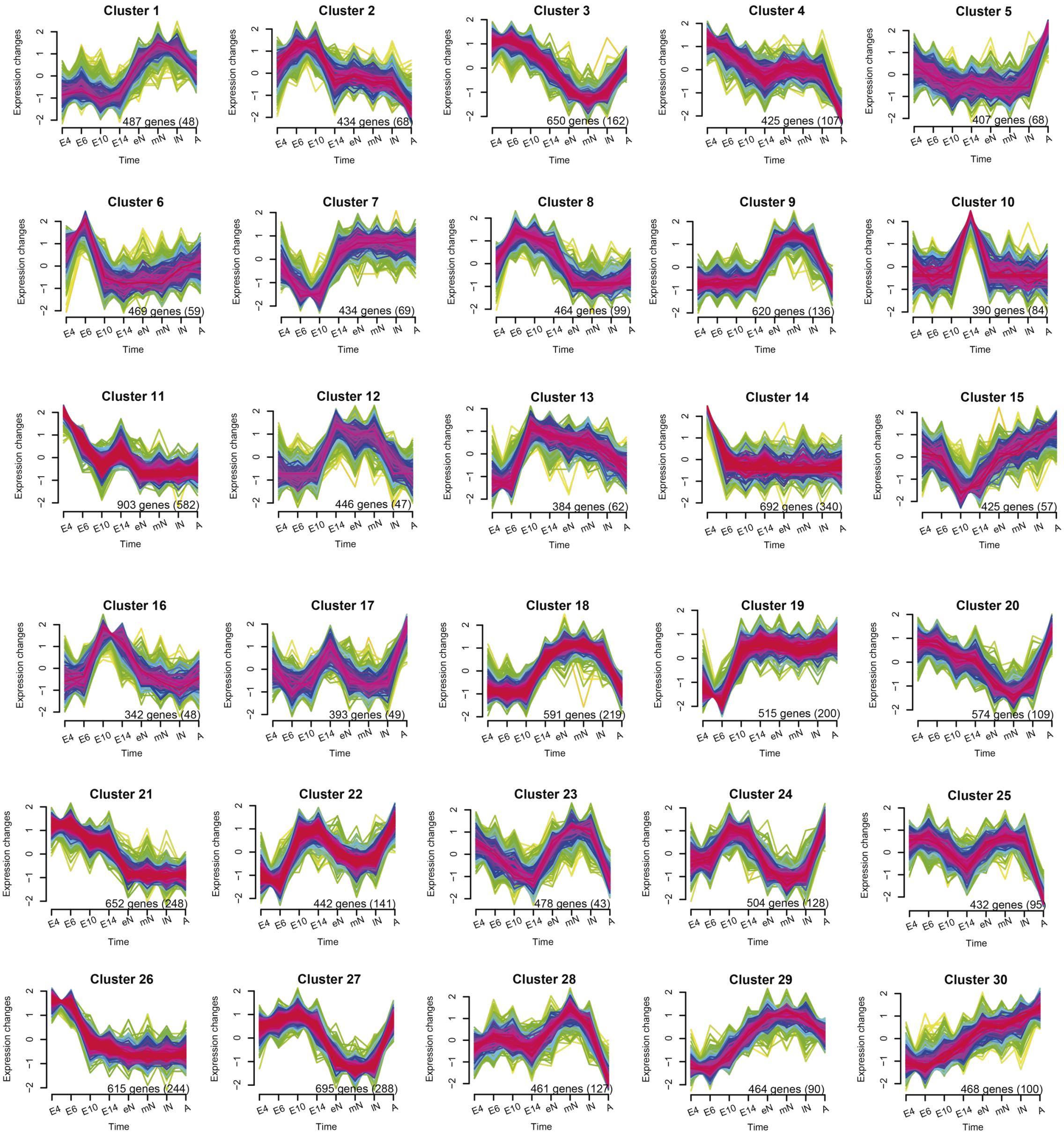

Supplementary Figure 2. Dynamics of gene expression along *C. dipterum* life cycle. Relative expression changes along RNA-seq samples, corresponding to different life cycle stages obtained with Mfuzz R package (E4: 4 days post fertilization (dpf) embryo, E6: 6 dpf embryo, E10: 10 dpf embryo, E14: 14 dpf embryo, eN: early nymph head, mN: mid nymph head, lN: late nymph head, A: adult head). Numbers in plot show the number of genes and the number of core genes (in brackets, with alpha score above 0.7) assigned to each cluster

Supplementary Figure 3

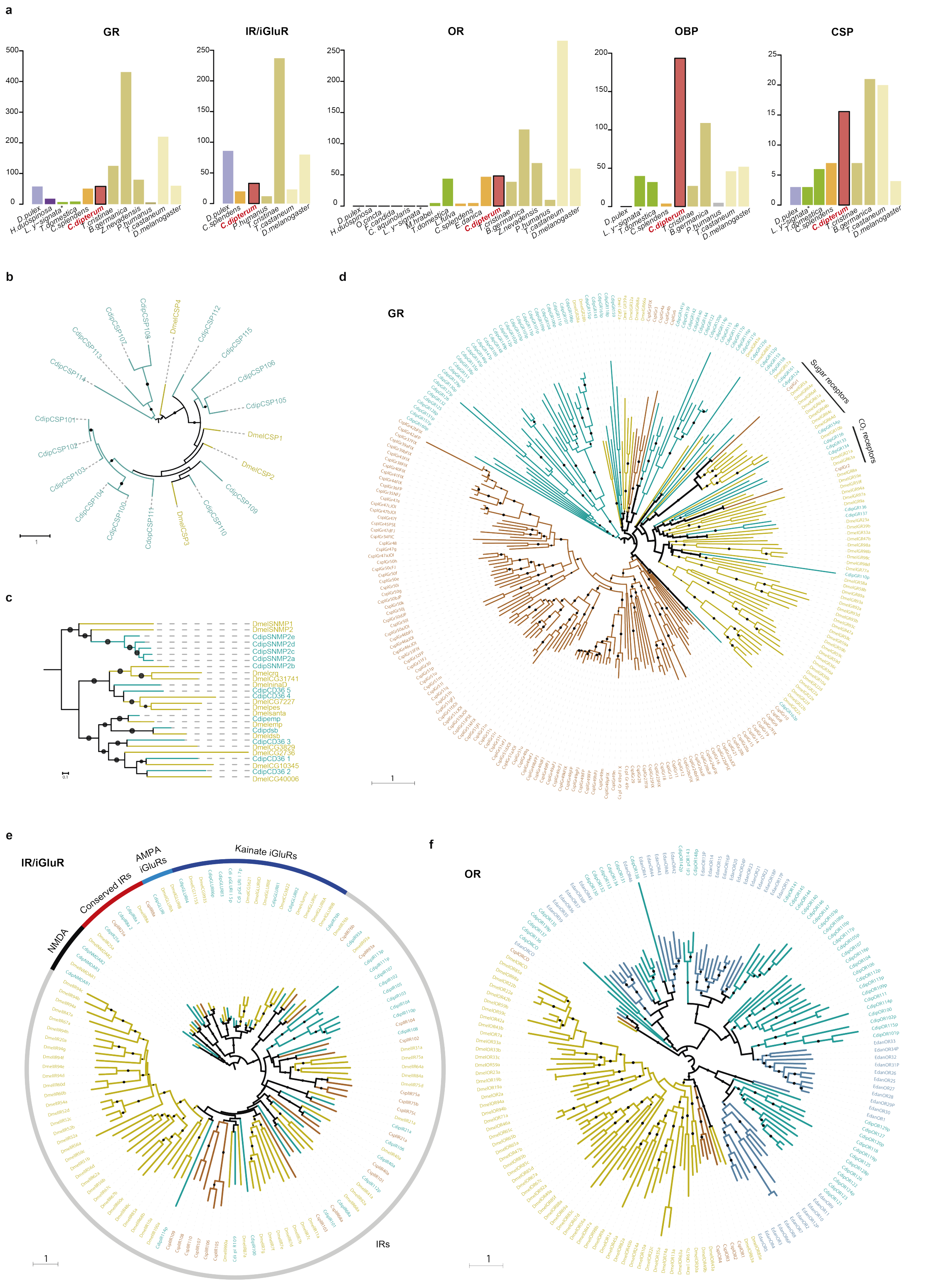

Supplementary Figure 3. Chemosensory gene families in *C. dipterum* genome. a, Bar plots exhibiting the CS gene complement in several pancrustacea species (*D. pulex,* Crustacea; *H. duospinosa, O. cincta, F. candida,* Collembola; *C. aquinolaris*, Diplura; *M. hrabei*, *L. y-signata*, Archaeognatha*; T. domestica,* Zygentoma; *L. fulva, C. splendens,* Odonata; *E.*

danica, C. dipterum, Ephemeroptera; *P. humanus*, Psocodea; *T. cristinae*, Phasmatodea; *B. germanica, Z. nevadensis,* Blattodea; *T. castaneum,* Coleoptera; *D. melanogaster,* Diptera). **b**, CSP gene family phylogeny. **c,** SNMP and CD36 gene families and phylogeny. **d,** GR phylogenetic tree. **e,** IR/iGluR phylogeny. **f,** OR gene complement and phylogeny. *C. dipterum* genes are shown in green, *D. melanogaster* shown in mustard, *E. danica* in blue and *C. splendens* genes are shown in brown.

Supplementary Figure 4

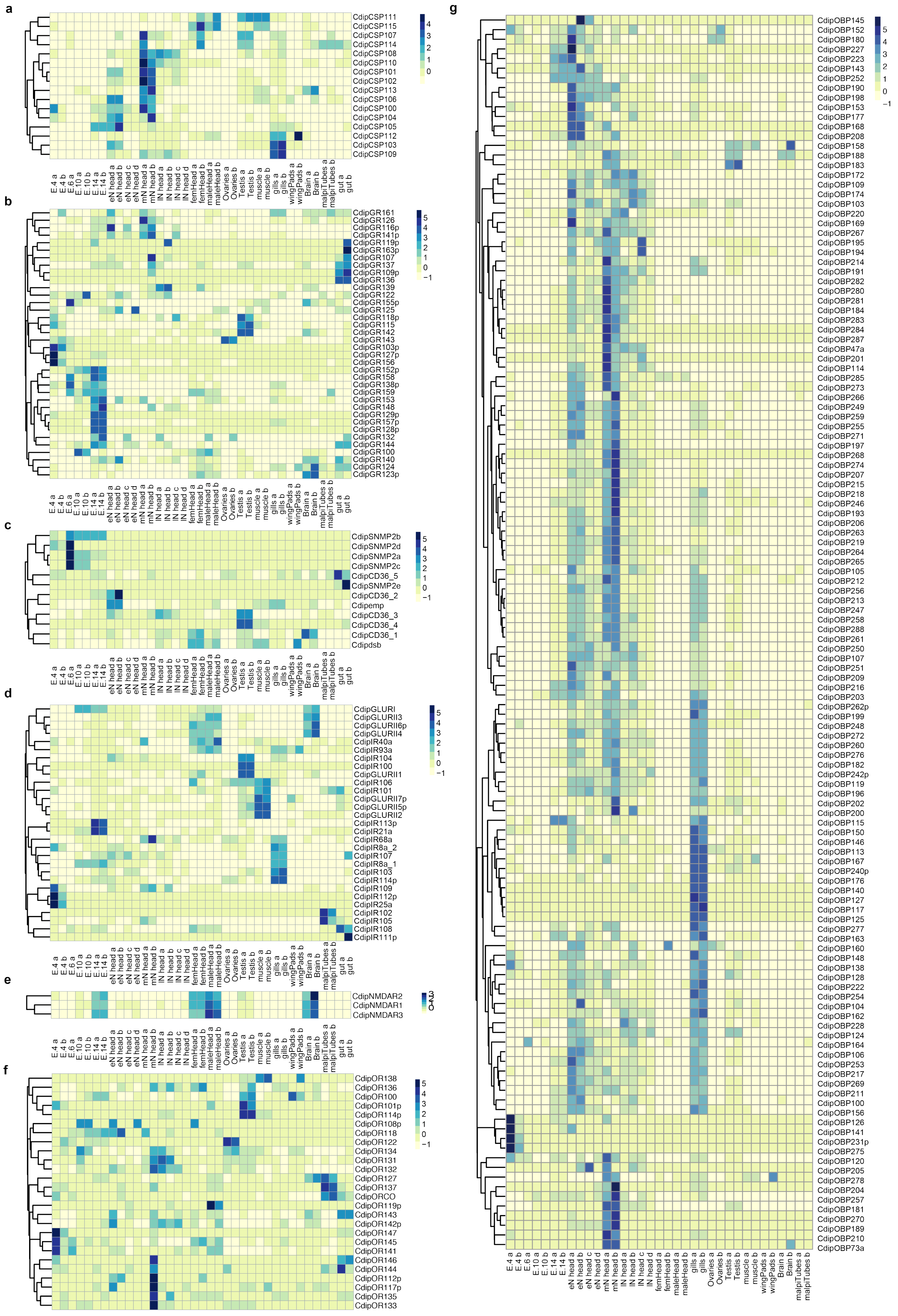

Supplementary Figure 4. Expression of chemosensory gene families across tissues and developmental stages. **a,** CSP gene family. **b,** GRs. **c,** SNMP and CD36 genes. **d,** IR/iGluR *C. dipterum* genes. **e,** NMDAR genes. **f,** OR genes. **g,** OBP genes. Blue corresponds to high expression; light yellow corresponds to low expression.

Supplementary Figure 5

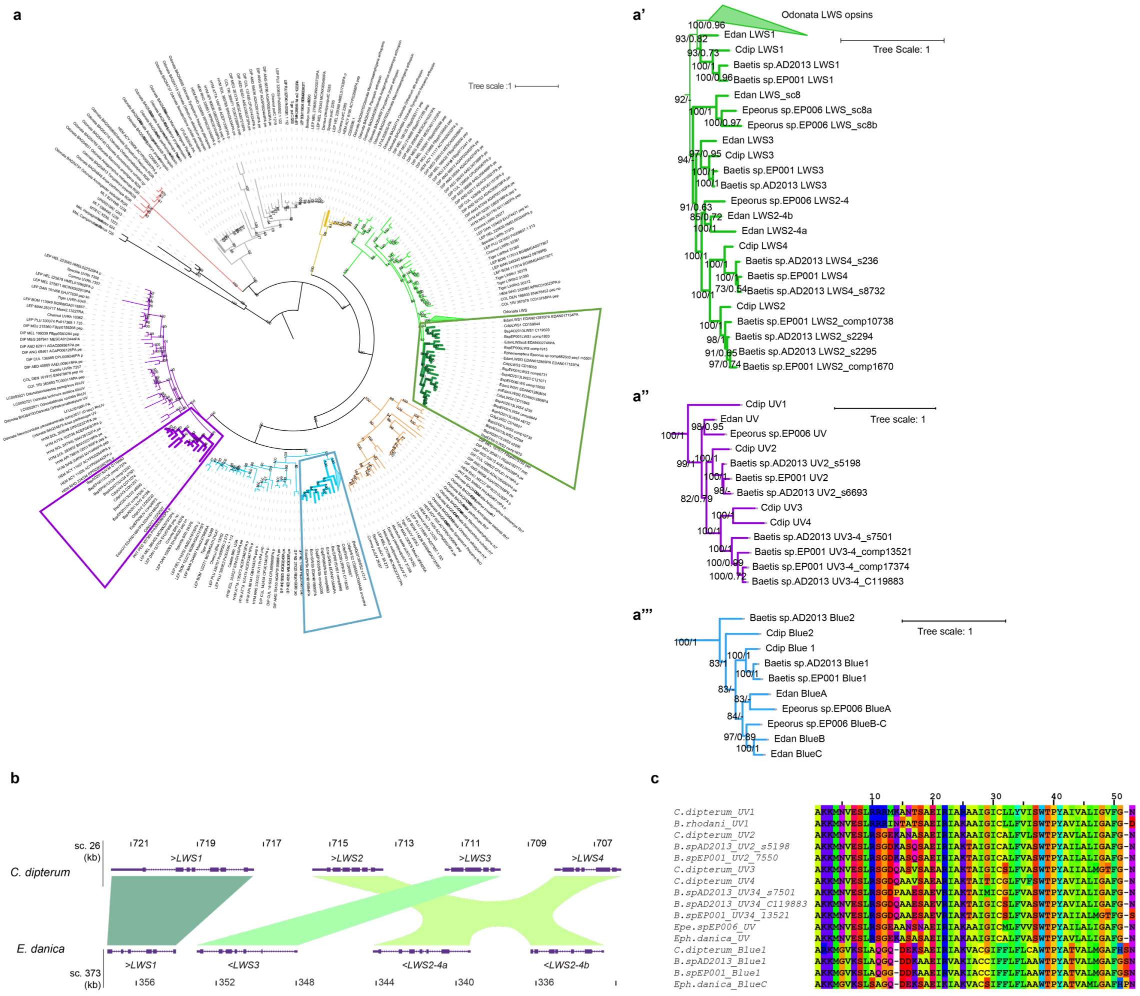

Supplementary Figure 5. Opsin complement in *C. dipterum*. a, ML-tree of opsin genes from different insects (DIP, Diptera; HYM, Hymenoptera; HEM, Hemiptera; COL, Coleoptera; Odonata and Ephemeroptera). Black branches represent melatonin receptor, which was chosen as outgroup; in red, RGR genes; in grey, pteropsins; arthropsin in mustard; in green, LWS opsin; in orange Rh7; in blue, Blue sensitive opsins and in violet, UV-sensitive opsins**. a',** Detail of Ephemeroptera LWS Opsin phylogeny. Numbers at each node represent boostrap and posterior probabilities values, respectively. **a'',** UV-sensitive Opsin. **a''',** Blue sensitive Opsins in Ephemeroptera. **b,** Ancestral expansion of LWS opsins in Ephemeroptera is located in a genomic cluster. Upper track shows *C. dipterum* genomic region and lower track shows *E. danica* region. **c,** Alignment of exon 6 of *C. dipterum* UV-Ops1 with its orthologous region from *B. rhodani* and homologous regions from other mayflies.

Supplementary Figure 6

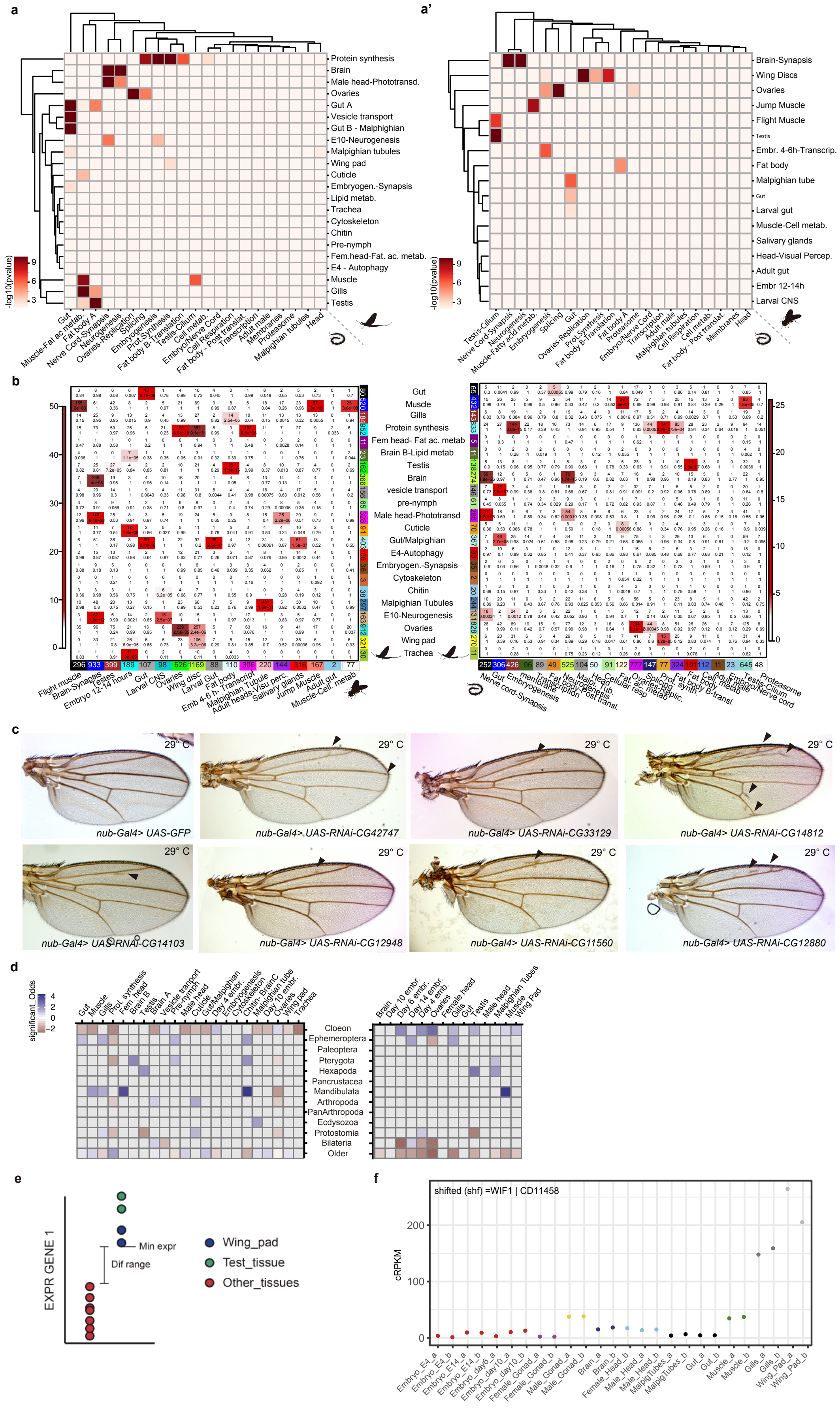

Supplementary Figure 6. Transcriptomic conservation of wings and other insect tissues. **a,** Orthologous gene overlap between modules from *C. dipterum* (vertical) and *S. maritima* (horizontal) obtained by weighted correlation network analysis (WGCNA) shown by levels of raw statistical significance. **a'**, Overlap of orthologues between modules from *D. melanogaster* (vertical) and *S. maritima* (horizontal) obtained by weighted correlation network analysis (WGCNA) shown by levels of raw statistical significance. **b,** Heatmaps showing module preservation between *C. dipterum* (horizontal) and *D. melanogaster* (vertical, left) and *S. maritima* (vertical, right). Numbers in colour boxes show the number of genes in each of the modules (Note that these analyses were done only with one-to-one orthologs). Heatmap colour code represents p-values, which are shown in each of the heatmap cells, together with the number of genes. **c,** Wing phenotypes observed when knocking down orthologous genes (*CG42747, CG33129, CG14812, CG14103, CG12948, CG11560* and *CG12880*) shared between *C. dipterum* and *D. melanogaster* wing and wing disc modules. Control wings: *nub-Gal4; UAS-GFP*. Extra vein territories are highlighted by black arrowheads. **d,** Heat maps showing enrichment test on gene age/phylostrata in WGCNA modules (left heat map) and tissue-specific RNA datasets (right heat map) relative to the background proportion of ages of the species of interest (p-value < 0.01). **e,** Scheme showing the methodology used to assign transcriptomic similarities between tissues. **f,** CD11458, which *D. melanogaster* orthologue is *shifted*, is highly expressed in wings and gills, in comparison to the rest of the tissue-specific RNA samples

Supplementary Figure 7

a

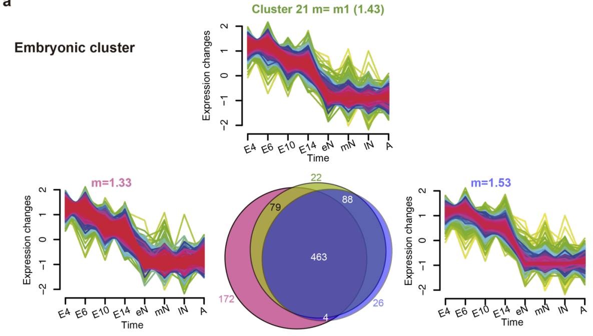

ID	GO term	m=1.43	m=1.33	m=1.53
GO:0010468	regulation of gene expression	3,90E-05	3,50E-10	2,90E-04
GO:0032774	RNA biosynthetic process	7,80E-05	5,00E-08	1,10E-04
GO:0014017	neuroblast fate commitment	8,70E-05	-	3,50E-05
GO:0015672	monovalent inorganic cation transport	9,30E-05	-	8,10E-04
GO:0006351	transcription, DNA-templated	1,30E-04	8,80E-08	1,80E-04
GO:0003676	nucleic acid binding	1,40E-06	3,50E-10	6,80E-06
GO:0003677	DNA binding	2,80E-06	7,40E-09	6,10E-06
GO:0043565	sequence-specific DNA binding	7,00E-06	1,70E-07	1,10E-06
GO:0000977	RNA polymerase II regulatory region sequ...	5,80E-05	2,80E-05	3,40E-05
GO:1990837	sequence-specific double-stranded DNA bi...	1,50E-04	3,50E-05	6,50E-05
GO:0005634	nucleus	7,30E-06	5,80E-10	3,80E-04
GO:0033180	proton-transporting V-type ATPase, V1 do...	2,80E-05	8,90E-04	0,00405
GO:0044427	chromosomal part	2,00E-04	8,90E-06	0,00168
GO:0005694	chromosome	2,90E-04	1,00E-05	6,90E-04
GO:0000785	chromatin	4,70E-04	1,60E-05	0,00401

b

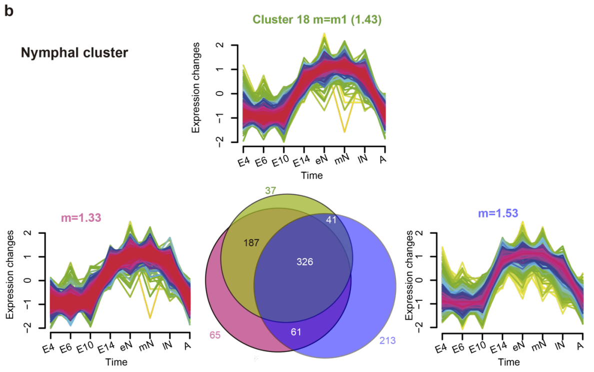

ID	GO term	m=1.43	m=1.33	m=1.53
GO:0009620	response to fungus	1,30E-08	6,50E-08	1,30E-04
GO:0007606	sensory perception of chemical stimulus	2,50E-08	2,50E-08	7,50E-05
GO:0040003	chitin-based cuticle development	2,30E-07	2,00E-09	6,90E-09
GO:0050830	defense response to Gram-positive bacter...	6,70E-07	2,00E-06	1,10E-06
GO:0045752	positive regulation of Toll signaling pa...	8,40E-07	1,90E-07	1,00E-07
GO:0005576	extracellular region	7,60E-26	5,30E-25	1,30E-18
GO:0044421	extracellular region part	4,00E-08	1,80E-07	5,60E-07
GO:0036379	myofilament	7,30E-08	5,50E-06	0,0293
GO:0031012	extracellular matrix	5,00E-06	5,50E-08	1,40E-06
GO:0005615	extracellular space	1,30E-05	8,20E-05	1,00E-04
GO:0005549	odorant binding	7,20E-16	1,30E-13	5,10E-08
GO:0008010	structural constituent of chitin-based l...	5,30E-11	2,90E-13	8,40E-11
GO:0042302	structural constituent of cuticle	1,70E-09	2,10E-11	2,70E-09
GO:0004252	serine-type endopeptidase activity	5,30E-07	1,80E-06	8,40E-07
GO:0070011	peptidase activity, acting on L-amino ac...	1,90E-06	1,20E-04	4,10E-04

c

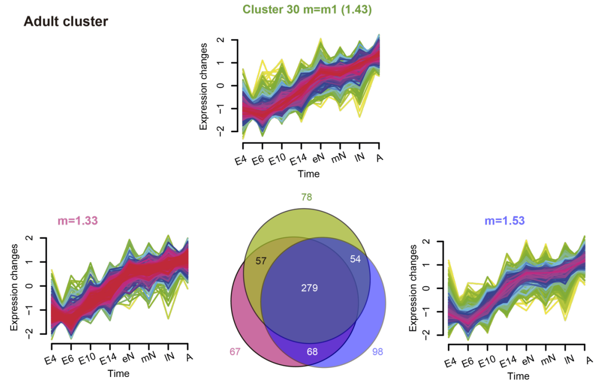

ID	GO term	m=1.43	m=1.33	m=1.53
GO:0009584	detection of visible light	3,20E-17	6,30E-15	1,70E-13
GO:0009583	detection of light stimulus	3,50E-14	2,60E-12	4,60E-11
GO:0050953	sensory perception of light stimulus	4,40E-14	2,90E-09	3,90E-09
GO:0007602	phototransduction	4,60E-14	4,30E-12	8,00E-11
GO:0007601	visual perception	2,30E-13	1,40E-08	2,10E-09
GO:0016028	rhabdomere	1,30E-12	2,40E-10	5,20E-09
GO:0016027	inaD signaling complex	4,50E-07	3,20E-07	3,70E-07
GO:0016021	integral component of membrane	2,80E-06	5,50E-07	1,30E-07
GO:0031224	intrinsic component of membrane	4,20E-06	8,20E-07	2,10E-07
GO:0005902	microvillus	4,30E-06	6,10E-05	0,001
GO:0009881	photoreceptor activity	1,20E-08	1,20E-05	3,00E-04
GO:0004888	transmembrane signaling receptor activit...	7,00E-05	1,40E-04	5,70E-05
GO:0008509	anion transmembrane transporter activity	1,10E-04	0,01575	9,40E-04
GO:0060089	molecular transducer activity	2,40E-04	9,60E-04	5,10E-04
GO:0004930	G protein-coupled receptor activity	2,60E-04	0,00286	0,0034

Supplementary Figure 7. Mfuzz analyses show cluster robustness. Relative expression changes along RNA-seq samples, corresponding to embryonic (**a**), nymphal (**b**) and adult (**c**) Mfuzz clusters maintain their structure with different values of the 'm' parameter (m= m1 (1.43 as in Figure 1, Figure 2 and Supplementary Figure 2), m= 1.33 and m= 1.53). Venn diagrams show gene overlap of clusters for each category. Tables show enriched GO terms for genes assigned to these clusters and calculated p-values as two-sided Fisher's exact tests. (E4: 4 days post fertilization (dpf) embryo, E6: 6 dpf embryo, E10: 10 dpf embryo, E14: 14 dpf embryo, eN: early nymph head, mN: mid nymph head, lN: late nymph head, A: adult head).

Supplementary References

1 Almudi, I. *et al.* Establishment of the mayfly Cloeon dipterum as a new model system to investigate insect evolution. *Evodevo* **10**, 6, doi:10.1186/s13227-019-0120-y (2019).

2 Marcais, G. & Kingsford, C. A fast, lock-free approach for efficient parallel counting of occurrences of k-mers. *Bioinformatics* **27**, 764-770, doi:10.1093/bioinformatics/btr011 (2011).

3 Liu, B. *et al.* Estimation of genomic characteristics by analyzing k-mer frequency in de novo genome projects. *arXiv:1308.2012* (2013).

4 Zimin, A. V. *et al.* Hybrid assembly of the large and highly repetitive genome of Aegilops tauschii, a progenitor of bread wheat, with the MaSuRCA mega-reads algorithm. *Genome Research* **27**, 787-792, doi:10.1101/gr.213405.116 (2017).

5 Zimin, A. V. *et al.* The MaSuRCA genome assembler. *Bioinformatics* **29**, 2669-2677, doi:10.1093/bioinformatics/btt476 (2013).

6 Simpson, J. T. Exploring genome characteristics and sequence quality without a reference. *Bioinformatics* **30**, 1228-1235, doi:10.1093/bioinformatics/btu023 (2014).

7 Wood, D. & Salzberg, S. Kraken: ultrafast metagenomic sequence classification using exact alignments. *Genome Biology* **15**, R46 (2014).

8 Marco-Sola, S., Sammeth, M., Guigo, R. & Ribeca, P. The GEM mapper: fast, accurate and versatile alignment by filtration. *Nat Methods*, doi:10.1038/nmeth.2221 (2012).

9 Li, H. Aligning sequence reads, clone sequences and assembly contigs with BWA-MEM. *arXiv: 1303.3997* (2013).

10 Zimin, A. V. *et al.* Hybrid assembly of the large and highly repetitive genome of Aegilops tauschii, a progenitor of bread wheat, with the mega-reads algorithm. *bioRxiv*, doi:10.1101/066100 (2016).

11 Parra, G., Bradnam, K. & Korf, I. CEGMA: a pipeline to accurately annotate core genes in eukaryotic genomes. *Bioinformatics* **23**, 1061-1067, doi:10.1093/bioinformatics/btm071 (2007).

12 Simão, F. A., Waterhouse, R. M., Ioannidis, P., Kriventseva, E. V. & Zdobnov, E. M. BUSCO: assessing genome assembly and annotation completeness with single-copy orthologs. *Bioinformatics* **31**, doi:10.1093/bioinformatics/btv351 (2015).

13 Grabherr, M. G. *et al.* Full-length transcriptome assembly from RNA-Seq data without a reference genome. *Nat Biotechnol* **29**, 644-652, doi:10.1038/nbt.1883 (2011).

14 Hass, J., Blaschke, S., Rammsayer, T. & Herrmann, J. M. A neurocomputational model for optimal temporal processing. *J Comput Neurosci* **25**, 449-464, doi:10.1007/s10827-008-0088-4 (2008).

15 Stanke, M., Tzvetkova, A. & Morgenstern, B. AUGUSTUS at EGASP: using EST, protein and genomic alignments for improved gene prediction in the human genome. *Genome Biol* **7 Suppl 1**, S11 11-18, doi:10.1186/gb-2006-7-s1-s11 (2006).

16 Dobin, A. *et al.* STAR: ultrafast universal RNA-seq aligner. *Bioinformatics* **29**, 15-21, doi:10.1093/bioinformatics/bts635 (2013).

17 Pertea, M. *et al.* StringTie enables improved reconstruction of a transcriptome from RNA-seq reads. *Nat Biotechnol* **33**, 290-295, doi:10.1038/nbt.3122 (2015).

18 Niknafs, Y. S., Pandian, B., Iyer, H. K., Chinnaiyan, A. M. & Iyer, M. K. TACO produces robust multisample transcriptome assemblies from RNA-seq. *Nat Methods* **14**, 68-70, doi:10.1038/nmeth.4078 (2017).

19 Emms, D. M. & Kelly, S. OrthoFinder: phylogenetic orthology inference for comparative genomics. *Genome Biology* **20**, 238, doi:10.1186/s13059-019-1832-y (2019).

20 Alexa, A., Rahnenfuhrer, J. & Lengauer, T. Improved scoring of functional groups from gene expression data by decorrelating GO graph structure. *Bioinformatics* **22**, 1600-1607, doi:10.1093/bioinformatics/btl140 (2006).

21 Smit, A. & Hubley, R. RepeatModeler Open-1.0. . http://www.repeatmasker.org/ (2008).

22 Bao, W., Kojima, K. K. & Kohany, O. Repbase Update, a database of repetitive elements in eukaryotic genomes. *Mob DNA* **6**, 11, doi:10.1186/s13100-015-0041-9 (2015).

23 Smit, A., Hubley, R. & Green, P. RepeatMasker Open-4.0. http://www.repeatmasker.org/ (2013).

24 Elsik, C. G. *et al.* Finding the missing honey bee genes: lessons learned from a genome upgrade. *BMC Genomics* **15**, 86, doi:10.1186/1471-2164-15-86 (2014).

25 Colbourne, J. K. *et al.* The Ecoresponsive Genome of Daphnia pulex. *Science* **331**, 555-561, doi:10.1126/science.1197761 (2011).

26 Clark, A. G. *et al.* Evolution of genes and genomes on the Drosophila phylogeny. *Nature* **450**, 203-218, doi:10.1038/nature06341 (2007).

27 Adams, M. D. *et al.* The Genome Sequence of Drosophila melanogaster. *Science* **287**, 2185-2195, doi:10.1126/science.287.5461.2185 (2000).

28 Thomas, G. W. C. *et al.* Gene content evolution in the arthropods. *Genome Biology* **21**, 15, doi:10.1186/s13059-019-1925-7 (2020).

29 Faddeeva-Vakhrusheva, A. *et al.* Coping with living in the soil: the genome of the parthenogenetic springtail Folsomia candida. *BMC Genomics* **18**, 493, doi:10.1186/s12864-017-3852-x (2017).

30 Kirkness, E. F. *et al.* Genome sequences of the human body louse and its primary endosymbiont provide insights into the permanent parasitic lifestyle. *Proceedings of the National Academy of Sciences* **107**, 12168-12173, doi:10.1073/pnas.1003379107 (2010).

31 Sanggaard, K. W. *et al.* Spider genomes provide insight into composition and evolution of venom and silk. *Nature Communications* **5**, 3765, doi:10.1038/ncomms4765 (2014).

32 Chipman, A. D. *et al.* The First Myriapod Genome Sequence Reveals Conservative Arthropod Gene Content and Genome Organisation in the Centipede Strigamia maritima. *PLOS Biology* **12**, e1002005, doi:10.1371/journal.pbio.1002005 (2014).

33 Barreto, F. S. *et al.* Genomic signatures of mitonuclear coevolution across populations of Tigriopus californicus. *Nature Ecology & Evolution* **2**, 1250-1257, doi:10.1038/s41559-018-0588-1 (2018).

34 Shelton, J. M. *et al.* Tools and pipelines for BioNano data: molecule assembly pipeline and FASTA super scaffolding tool. *BMC Genomics* **16**, 734, doi:10.1186/s12864-015-1911-8 (2015).

35 Terrapon, N. *et al.* Molecular traces of alternative social organization in a termite genome. *Nature Communications* **5**, 3636, doi:10.1038/ncomms4636 (2014).

36 Buchfink, B., Xie, C. & Huson, D. H. Fast and sensitive protein alignment using DIAMOND. *Nature Methods* **12**, 59, doi:10.1038/nmeth.3176 https://www.nature.com/articles/nmeth.3176 - supplementary-information (2014).

37 Katoh, K. & Standley, D. M. MAFFT multiple sequence alignment software version 7: improvements in performance and usability. *Molecular biology and evolution* **30**, 772-780, doi:10.1093/molbev/mst010 (2013).

38 Capella-Gutiérrez, S., Silla-Martínez, J. M. & Gabaldón, T. trimAl: a tool for automated alignment trimming in large-scale phylogenetic analyses. *Bioinformatics* **25**, 1972-1973, doi:10.1093/bioinformatics/btp348 (2009).

39 Kozlov, A. M., Darriba, D., Flouri, T., Morel, B. & Stamatakis, A. RAxML-NG: a fast, scalable and user-friendly tool for maximum likelihood phylogenetic inference. *Bioinformatics*, doi:10.1093/bioinformatics/btz305 (2019).

40 Bowles, A. M. C., Bechtold, U. & Paps, J. The Origin of Land Plants Is Rooted in Two Bursts of Genomic Novelty. *Current Biology* **30**, 530-536.e532, doi:https://doi.org/10.1016/j.cub.2019.11.090 (2020).

41 Guijarro-Clarke, C., Holland, P. W. H. & Paps, J. Widespread patterns of gene loss in the evolution of the animal kingdom. *Nature Ecology & Evolution*, doi:10.1038/s41559-020-1129-2 (2020).

42 Paps, J. & Holland, P. W. H. Reconstruction of the ancestral metazoan genome reveals an increase in genomic novelty. *Nature Communications* **9**, 1730, doi:10.1038/s41467-018-04136-5 (2018).

43 Enright, A. J., Van Dongen, S. & Ouzounis, C. A. An efficient algorithm for large-scale detection of protein families. *Nucleic acids research* **30**, 1575-1584, doi:10.1093/nar/30.7.1575 (2002).

44 Mi, H. *et al.* Protocol Update for large-scale genome and gene function analysis with the PANTHER classification system (v.14.0). *Nature Protocols* **14**, 703-721, doi:10.1038/s41596-019-0128-8 (2019).

45 Kumar, L. & E Futschik, M. Mfuzz: a software package for soft clustering of microarray data. *Bioinformation* **2**, 5-7, doi:10.6026/97320630002005 (2007).

46 Kim, D., Paggi, J. M., Park, C., Bennett, C. & Salzberg, S. L. Graph-based genome alignment and genotyping with HISAT2 and HISAT-genotype. *Nature Biotechnology* **37**, 907-915, doi:10.1038/s41587-019-0201-4 (2019).

47 Anders, S., Pyl, P. T. & Huber, W. HTSeq—a Python framework to work with high-throughput sequencing data. *Bioinformatics* **31**, 166-169, doi:10.1093/bioinformatics/btu638 (2014).

48 Love, M. I., Huber, W. & Anders, S. Moderated estimation of fold change and dispersion for RNA-seq data with DESeq2. *Genome Biol* **15**, 550, doi:10.1186/s13059-014-0550-8 (2014).

49 Labbé, R. M. *et al.* A Comparative Transcriptomic Analysis Reveals Conserved Features of Stem Cell Pluripotency in Planarians and Mammals. *STEM CELLS* **30**, 1734-1745, doi:10.1002/stem.1144 (2012).

50 Torres-Méndez, A. *et al.* A novel protein domain in an ancestral splicing factor drove the evolution of neural microexons. *Nature Ecology & Evolution* **3**, 691-701, doi:10.1038/s41559-019-0813-6 (2019).

51 Langfelder, P. & Horvath, S. WGCNA: an R package for weighted correlation network analysis. *BMC Bioinformatics* **9**, 559, doi:10.1186/1471-2105-9-559 (2008).

52 Harrison, M. C. *et al.* Hemimetabolous genomes reveal molecular basis of termite eusociality. *Nature Ecology & Evolution* **2**, 557-566, doi:10.1038/s41559-017-0459-1 (2018).

53 Benton, R., Vannice, K. S., Gomez-Diaz, C. & Vosshall, L. B. Variant ionotropic glutamate receptors as chemosensory receptors in Drosophila. *Cell* **136**, 149-162, doi:10.1016/j.cell.2008.12.001 (2009).

54 Vogt, R. G. *et al.* The insect SNMP gene family. *Insect Biochem Mol Biol* **39**, 448-456, doi:10.1016/j.ibmb.2009.03.007 (2009).

55 Vieira, F. G. & Rozas, J. Comparative genomics of the odorant-binding and chemosensory protein gene families across the Arthropoda: origin and evolutionary history of the chemosensory system. *Genome Biol Evol* **3**, 476-490, doi:10.1093/gbe/evr033 (2011).

56 Ioannidis, P. *et al.* Genomic Features of the Damselfly Calopteryx splendens Representing a Sister Clade to Most Insect Orders. *Genome Biol Evol* **9**, 415-430, doi:10.1093/gbe/evx006 (2017).

57 Missbach, C. *et al.* Evolution of insect olfactory receptors. *Elife* **3**, e02115, doi:10.7554/eLife.02115 (2014).

58 Kirkness, E. F. *et al.* Genome sequences of the human body louse and its primary endosymbiont provide insights into the permanent parasitic lifestyle. *Proc Natl Acad Sci U S A* **107**, 12168-12173, doi:10.1073/pnas.1003379107 (2010).

59 Terrapon, N. *et al.* Molecular traces of alternative social organization in a termite genome. *Nat Commun* **5**, 3636, doi:10.1038/ncomms4636 (2014).

60 Wu, C. *et al.* Analysis of the genome of the New Zealand giant collembolan (Holacanthella duospinosa) sheds light on hexapod evolution. *BMC Genomics* **18**, 795-795, doi:10.1186/s12864-017-4197-1 (2017).

61 Brand, P. *et al.* The origin of the odorant receptor gene family in insects. *eLife* **7**, e38340, doi:10.7554/eLife.38340 (2018).

62 Frias-Lopez, C. *et al.* Comparative analysis of tissue-specific transcriptomes in the funnel-web spider Macrothele calpeiana (Araneae, Hexathelidae). *PeerJ* **3**, e1064, doi:10.7717/peerj.1064 (2015).

63 Vizueta, J., Sánchez-Gracia, A. & Rozas, J. BITACORA: A comprehensive tool for the identification and annotation of gene families in genome assemblies. *bioRxiv*, 593889, doi:10.1101/593889 (2019).

64 Vizueta, J., Rozas, J. & Sánchez-Gracia, A. Comparative Genomics Reveals Thousands of Novel Chemosensory Genes and Massive Changes in Chemoreceptor Repertories across Chelicerates. *Genome biology and evolution* **10**, 1221-1236, doi:10.1093/gbe/evy081 (2018).

65 Krogh, A., Larsson, B., von Heijne, G. & Sonnhammer, E. L. Predicting transmembrane protein topology with a hidden Markov model: application to complete genomes. *J Mol Biol* **305**, 567-580, doi:10.1006/jmbi.2000.4315 (2001).

66 Kall, L., Krogh, A. & Sonnhammer, E. L. A combined transmembrane topology and signal peptide prediction method. *J Mol Biol* **338**, 1027-1036, doi:10.1016/j.jmb.2004.03.016 (2004).

67 Croset, V. *et al.* Ancient protostome origin of chemosensory ionotropic glutamate receptors and the evolution of insect taste and olfaction. *PLoS Genet* **6**, e1001064, doi:10.1371/journal.pgen.1001064 (2010).

68 Petersen, T. N., Brunak, S., von Heijne, G. & Nielsen, H. SignalP 4.0: discriminating signal peptides from transmembrane regions. *Nat Methods* **8**, 785-786, doi:10.1038/nmeth.1701 (2011).

69 Katoh, K. & Standley, D. M. MAFFT multiple sequence alignment software version 7: improvements in performance and usability. *Molecular biology and evolution* **30**, 772-780, doi:10.1093/molbev/mst010 (2013).

70 Nguyen, L. T., Schmidt, H. A., von Haeseler, A. & Minh, B. Q. IQ-TREE: a fast and effective stochastic algorithm for estimating maximum-likelihood phylogenies. *Molecular biology and evolution* **32**, 268-274, doi:10.1093/molbev/msu300 (2015).

71 Hoang, D. T. *et al.* MPBoot: fast phylogenetic maximum parsimony tree inference and bootstrap approximation. *BMC evolutionary biology* **18**, 11-11, doi:10.1186/s12862-018-1131-3 (2018).

72 Letunic, I. & Bork, P. Interactive Tree Of Life (iTOL): an online tool for phylogenetic tree display and annotation. *Bioinformatics* **23**, 127-128, doi:10.1093/bioinformatics/btl529 (2007).

73 Li, L., Stoeckert, C. J., Jr. & Roos, D. S. OrthoMCL: identification of ortholog groups for eukaryotic genomes. *Genome Res* **13**, 2178-2189, doi:10.1101/gr.1224503 (2003).

74 Robertson, H. M., Baits, R. L., Walden, K. K. O., Wada-Katsumata, A. & Schal, C. Enormous expansion of the chemosensory gene repertoire in the omnivorous German cockroach Blattella germanica. *Journal of Experimental Zoology Part B: Molecular and Developmental Evolution* **330**, 265-278, doi:10.1002/jez.b.22797 (2018).

75 Librado, P. & Rozas, J. Uncovering the functional constraints underlying the genomic organization of the odorant-binding protein genes. *Genome Biol Evol* **5**, 2096-2108, doi:10.1093/gbe/evt158 (2013).

76 Mei, T., Fu, W.-B., Li, B., He, Z.-B. & Chen, B. Comparative genomics of chemosensory protein genes (CSPs) in twenty-two mosquito species (Diptera: Culicidae): Identification, characterization, and evolution. *PloS one* **13**, e0190412-e0190412, doi:10.1371/journal.pone.0190412 (2018).

77 Sanchez-Gracia, A., Vieira, F. G. & Rozas, J. Molecular evolution of the major chemosensory gene families in insects. *Heredity (Edinb)* **103**, 208-216, doi:10.1038/hdy.2009.55 (2009).

78 Penalva-Arana, D. C., Lynch, M. & Robertson, H. M. The chemoreceptor genes of the waterflea Daphnia pulex: many Grs but no Ors. *BMC Evol Biol* **9**, 79, doi:10.1186/1471-2148-9-79 (2009).

79 Knecht, Z. A. *et al.* Distinct combinations of variant ionotropic glutamate receptors mediate thermosensation and hygrosensation in Drosophila. *eLife* **5**, e17879, doi:10.7554/eLife.17879 (2016).

80 Gomez-Diaz, C. *et al.* A CD36 ectodomain mediates insect pheromone detection via a putative tunnelling mechanism. *Nat Commun* **7**, 11866, doi:10.1038/ncomms11866 (2016).

81 Schindelin, J. *et al.* Fiji: an open-source platform for biological-image analysis. *Nature Methods* **9**, 676-682, doi:10.1038/nmeth.2019 (2012).

82 Feuda, R., Hamilton, S. C., McInerney, J. O. & Pisani, D. Metazoan opsin evolution reveals a simple route to animal vision. *Proceedings of the National Academy of Sciences* **109**, 18868-18872, doi:10.1073/pnas.1204609109 (2012).

83 Futahashi, R. *et al.* Extraordinary diversity of visual opsin genes in dragonflies. *Proceedings of the National Academy of Sciences* **112**, E1247-E1256, doi:10.1073/pnas.1424670112 (2015).

84 Suvorov, A. *et al.* Opsins have evolved under the permanent heterozygote model: insights from phylotranscriptomics of Odonata. *Mol Ecol* **26**, 1306-1322, doi:10.1111/mec.13884 (2017).

85 Misof, B. *et al.* Phylogenomics resolves the timing and pattern of insect evolution. *Science* **346**, 763-767, doi:10.1126/science.1257570 (2014).

86 Macdonald, H. C., Ormerod, S. J. & Bruford, M. W. Enhancing capacity for freshwater conservation at the genetic level: a demonstration using three stream macroinvertebrates. *Aquatic Conservation: Marine and Freshwater Ecosystems* **27**, 452-461, doi:10.1002/aqc.2691 (2017).

87 Feuda, R., Marletaz, F., Bentley, M. A. & Holland, P. W. Conservation, Duplication, and Divergence of Five Opsin Genes in Insect Evolution. *Genome Biol Evol* **8**, 579-587, doi:10.1093/gbe/evw015 (2016).

88 Capella-Gutiérrez, S., Silla-Martínez, J. M. & Gabaldón, T. trimAl: a tool for automated alignment trimming in large-scale phylogenetic analyses. *Bioinformatics (Oxford, England)* **25**, 1972-1973, doi:10.1093/bioinformatics/btp348 (2009).

89 Hoang, D. T., Chernomor, O., von Haeseler, A., Minh, B. Q. & Vinh, L. S. UFBoot2: Improving the Ultrafast Bootstrap Approximation. *Molecular biology and evolution* **35**, 518-522, doi:10.1093/molbev/msx281 (2017).

90 Anisimova, M., Gil, M., Dufayard, J.-F., Dessimoz, C. & Gascuel, O. Survey of branch support methods demonstrates accuracy, power, and robustness of fast likelihood-based approximation schemes. *Systematic biology* **60**, 685-699, doi:10.1093/sysbio/syr041 (2011).

91 Nguyen, L.-T., Schmidt, H. A., von Haeseler, A. & Minh, B. Q. IQ-TREE: A Fast and Effective Stochastic Algorithm for Estimating Maximum-Likelihood Phylogenies. *Molecular biology and evolution* **32**, 268-274, doi:10.1093/molbev/msu300 (2014).

92 Le, S. Q. & Gascuel, O. An Improved General Amino Acid Replacement Matrix. *Molecular biology and evolution* **25**, 1307-1320, doi:10.1093/molbev/msn067 (2008).

93 Lartillot, N., Rodrigue, N., Stubbs, D. & Richer, J. PhyloBayes MPI: Phylogenetic Reconstruction with Infinite Mixtures of Profiles in a Parallel Environment. *Systematic Biology* **62**, 611-615, doi:10.1093/sysbio/syt022 (2013).

94 King, R. S. & Newmark, P. A. In situ hybridization protocol for enhanced detection of gene expression in the planarian Schmidtea mediterranea. *BMC developmental biology* **13**, 8-8, doi:10.1186/1471-213X-13-8 (2013).

95 Genome Sequence of the Nematode C. elegans: A Platform for Investigating Biology. *Science* **282**, 2012-2018, doi:10.1126/science.282.5396.2012 (1998).

96 Dasmahapatra, K. K. *et al.* Butterfly genome reveals promiscuous exchange of mimicry adaptations among species. *Nature* **487**, 94-98, doi:10.1038/nature11041 (2012).

97 Wu, C. *et al.* Analysis of the genome of the New Zealand giant collembolan (Holacanthella duospinosa) sheds light on hexapod evolution. *BMC Genomics* **18**, 795, doi:10.1186/s12864-017-4197-1 (2017).

98 Gulia-Nuss, M. *et al.* Genomic insights into the Ixodes scapularis tick vector of Lyme disease. *Nature Communications* **7**, 10507, doi:10.1038/ncomms10507 (2016).

99 Zhu, J. *et al.* Genome sequence of the small brown planthopper, Laodelphax striatellus. *GigaScience* **6**, doi:10.1093/gigascience/gix109 (2017).

100 Savojardo, C., Luchetti, A., Martelli, P. L., Casadio, R. & Mantovani, B. Draft genomes and genomic divergence of two Lepidurus tadpole shrimp species (Crustacea, Branchiopoda, Notostraca). *Molecular Ecology Resources* **19**, 235-244, doi:10.1111/1755-0998.12952 (2019).

101 Battelle, B.-A. *et al.* Opsin Repertoire and Expression Patterns in Horseshoe Crabs: Evidence from the Genome of Limulus polyphemus (Arthropoda: Chelicerata). *Genome Biology and Evolution* **8**, 1571-1589, doi:10.1093/gbe/evw100 (2016).

102 Luo, Y.-J. *et al.* The Lingula genome provides insights into brachiopod evolution and the origin of phosphate biomineralization. *Nature Communications* **6**, 8301, doi:10.1038/ncomms9301 (2015).

103 Wang, X. *et al.* The locust genome provides insight into swarm formation and long-distance flight. *Nature Communications* **5**, 2957, doi:10.1038/ncomms3957 (2014).

104 Putnam, N. H. *et al.* Sea Anemone Genome Reveals Ancestral Eumetazoan Gene Repertoire and Genomic Organization. *Science* **317**, 86-94, doi:10.1126/science.1139158 (2007).

105 Kao, D. *et al.* The genome of the crustacean Parhyale hawaiensis, a model for animal development, regeneration, immunity and lignocellulose digestion. *eLife* **5**, e20062, doi:10.7554/eLife.20062 (2016).

106 Zhang, X. *et al.* Penaeid shrimp genome provides insights into benthic adaptation and frequent molting. *Nature Communications* **10**, 356, doi:10.1038/s41467-018-08197-4 (2019).

107 Hashimoto, T. *et al.* Extremotolerant tardigrade genome and improved radiotolerance of human cultured cells by tardigrade-unique protein. *Nature Communications* **7**, 12808, doi:10.1038/ncomms12808 (2016).

108 Zhang, F. *et al.* A High-quality Draft Genome Assembly of Sinella curviseta: A Soil Model Organism (Collembola). *Genome Biology and Evolution* **11**, 521-530, doi:10.1093/gbe/evz013 (2019).

109 Emms, D. M. & Kelly, S. OrthoFinder: phylogenetic orthology inference for comparative genomics. *bioRxiv*, 466201, doi:10.1101/466201 (2019).
