# Supplementary material for: Genomic adaptations to aquatic and aerial life in mayflies and the origin of insect wings
Source: Nat Commun. 2020 May 26;11:2631. doi: 10.1038/s41467-020-16284-8 (PMC7250882; doi:10.1038/s41467-020-16284-8)
Supplement: Supplementary file 3 — Reporting Summary [file 41467_2020_16284_MOESM3_ESM.pdf]

nature research

Corresponding author(s): Isabel Almudi, Fernando Casares

Last updated by author(s): Apr 20, 2020

Reporting Summary

Nature Research wishes to improve the reproducibility of the work that we publish. This form provides structure for consistency and transparency in reporting. For further information on Nature Research policies, see Authors & Referees and the Editorial Policy Checklist .

Statistics

For all statistical analyses, confirm that the following items are present in the figure legend, table legend, main text, or Methods section.

n/a	Confirmed	
☐	☒	The exact sample size (*n*) for each experimental group/condition, given as a discrete number and unit of measurement
☒	☐	A statement on whether measurements were taken from distinct samples or whether the same sample was measured repeatedly
☐	☒	The statistical test(s) used AND whether they are one- or two-sided<br>*Only common tests should be described solely by name; describe more complex techniques in the Methods section.*
☒	☐	A description of all covariates tested
☐	☒	A description of any assumptions or corrections, such as tests of normality and adjustment for multiple comparisons
☐	☒	A full description of the statistical parameters including central tendency (e.g. means) or other basic estimates (e.g. regression coefficient) AND variation (e.g. standard deviation) or associated estimates of uncertainty (e.g. confidence intervals)
☐	☒	For null hypothesis testing, the test statistic (e.g. *F*, *t*, *r*) with confidence intervals, effect sizes, degrees of freedom and *P* value noted<br>*Give P values as exact values whenever suitable.*
☐	☒	For Bayesian analysis, information on the choice of priors and Markov chain Monte Carlo settings
☒	☐	For hierarchical and complex designs, identification of the appropriate level for tests and full reporting of outcomes
☒	☐	Estimates of effect sizes (e.g. Cohen's *d*, Pearson's *r*), indicating how they were calculated

Our web collection on statistics for biologists contains articles on many of the points above.

Software and code

Policy information about availability of computer code

Data collection

Leica SPE confocal microscope was used to acquire images of ISH experiments. No software was used for data collection

Data analysis

The following packages and software were used:
1. Kraken (v0.10.5-beta)
2. gce (v1.0.0)
3. Jellyfish (v2.2.0)
4. iTOL web server (v5.5.1)
5. SGA preqc (v0.10.13)
6. MaSuRCA (v3.2.3)
7. SignalP (v4.1)
8. CEGMA (v2.5)
9. BUSCO (v3)
10. FASTX Toolkit (v0.0.13)
11. gem-mapper (build 1.81)
12. Trinity (v2.2.0)
13. PASA (v2.0.2)
14. AUGUSTUS (v2.5.5)
15. STAR (v2.7)
16. StringTie (v1.3.4)
17. TACO (v0.7.3)
18. RepeatModeler (v1-0-11)
19. RepeatMasker (open-4.0.5) (http://www.repeatmasker.org)
20. PhastCons (v1.3)

21. pheatmap (1.0.12)
22. Portcullis (v1.1.2)
23. OrthoFinder 2
24. DIAMOND (0.9.15)
25. MAFFT(v7.221)
26. trimAl (v1.4)
27. RAxML (v8.2.1)
28. Mfuzz (Program version: v2.40.0; R version: 3.2.4)
29. hisat2 (v2.1.0)
30. htseq-count (v0.11.1)
31. samtools (v1.6)
32. DESeq2 (v1.20.0 R version: 3.2.4)
33. Weighted Gene Correlation network analysis (WGCNA) (Program version: v1.62; R version 3.3.2)
34. UCSC Kent utilities (downloaded Oct-22-2013): axtChain, chainMergeSort, chainPreNet, chainNet, multiz-tba (version 2009-Jan-21)
35. BITACORA (v1.0)
36. HMMER (v3.1b)
37. TMHMM (v2.0c)
38. Phobius (v1.01)
39. IQ-TREE (v1.6.5)
40. OrthoMCL (v2.0.9)
41. TopGO (R package version 2.36.0, R version: 3.2.4)
42. heatmaps package, Bioconductor (v.1.4.0)
43. ImageJ Fiji 64
44. Photoshop CS5.1
45. Illustrator CS5.1
46. Python (v2.7)
47. Python (v3.5)
48. Blast (v2.2.29)
49. phylobayes/1.8.intel
50. Pfam database (v30.0)
51. kentUtils (v302.1)
52. ggplot2 (v3.0.0; R version: 3.5.0)
53. CASAVA (v1.8)
54. MinKNOW (v1.10.11)
55. Albacore 2.1.0
56. BWA MEM (v0.7.12)
57. RTA 1.18.66.3
58. BLAST+ 2.9.0
59. perl (v5.14.12)

For manuscripts utilizing custom algorithms or software that are central to the research but not yet described in published literature, software must be made available to editors/reviewers. We strongly encourage code deposition in a community repository (e.g. GitHub). See the Nature Research guidelines for submitting code & software for further information.

Data

Policy information about availability of data

All manuscripts must include a data availability statement. This statement should provide the following information, where applicable:

- Accession codes, unique identifiers, or web links for publicly available datasets
- A list of figures that have associated raw data
- A description of any restrictions on data availability

All data generated and analysed during this study are available in European Nucleotide Archive (ENA) public repository with the project accessions PRJEB34721 and PRJEB35103. The assembly accession is GCA_902829235 (sample ID ERS4386951, contig accession CADEPI010000001-CADEPI010001395). All other RNA-Seq datasets and genome assemblies used in the study are publicly available and listed in Supplementary Table 12 and Supplementary Table 4, respectively.

Field-specific reporting

Please select the one below that is the best fit for your research. If you are not sure, read the appropriate sections before making your selection.

☒ Life sciences ☐ Behavioural & social sciences ☐ Ecological, evolutionary & environmental sciences

For a reference copy of the document with all sections, see nature.com/documents/nr-reporting-summary-flat.pdf

Life sciences study design

All studies must disclose on these points even when the disclosure is negative.

Sample size	No statistical method was used to predetermine sample size. For each cross-species transcriptomic comparisons as many orthologous genes as possible were used. For each next generation sequencing experiment, for each of the biological replicate we used as many embryos or amount of nymphal or adult tissues as necessary to obtain the enough amount of RNA/DNA for library preparation and sequencing according to previous experience in the laboratory .
Data exclusions	No data were excluded from the analyses
Replication	The findings reported in this study correspond to computational analyses of next generation sequencing data. We also perform two main types of experiments, largely for validation purposes: (i) Drosophila crosses to knockdown genes specifically in the wing and (ii) in situ hybridization of specialized families. The phenotypes after knocking down genes specifically in the wing were observed in all the individuals imaged (10 per each gene in two independent experiments). We have performed the in situ hybridizations at least twice each, using several individuals (between 7-12 individuals. For OBP genes, n>20 gills in all cases, while Ops genes were detected in n>5 retinas per each gene) per probe, and all of them showed the same pattern in every independent assay.
Randomization	We did not have experimental groups that apply here. In our study we compared either (i) different tissues and developmental stages within a species, or (ii) matched samples for different species.
Blinding	Not relevant. The statistical significance between transcriptomic samples were obtained automatically by software used to perform the analyses and not by the researchers.

Reporting for specific materials, systems and methods

We require information from authors about some types of materials, experimental systems and methods used in many studies. Here, indicate whether each material, system or method listed is relevant to your study. If you are not sure if a list item applies to your research, read the appropriate section before selecting a response.

Materials & experimental systems

n/a	Involved in the study
☐	☒ Antibodies
☒	☐ Eukaryotic cell lines
☒	☐ Palaeontology
☐	☒ Animals and other organisms
☒	☐ Human research participants
☒	☐ Clinical data

Methods

n/a	Involved in the study
☒	☐ ChIP-seq
☒	☐ Flow cytometry
☒	☐ MRI-based neuroimaging

Antibodies

Antibodies used	Goat anti-HRP-Cy3 1:100 (Jackson Immunoresearch, code 123-165-021), Anti-Digoxigenin-AP, Fab fragments 1:2000 (11093274910 Roche), Sheep Anti-Digoxigenin-POD, Fab fragments 1:150 (11207733910 Roche)
Validation	- Goat anti-HRP-Cy3 commercial antibody has been broadly used to detect neurons in different insect species<br>Jan, L. Y. & Jan, Y. N. Antibodies to horseradish peroxidase as specific neuronal markers in Drosophila and in grasshopper embryos. Proceedings of the National Academy of Sciences of the United States of America 79, 2700-2704, doi:10.1073/pnas.79.8.2700 (1982)<br>- Anti-Digoxigenin-AP and Anti-Digoxigenin-POD are high-quality commercial antibodies validated by the manufacturer (Roche):<br>https://www.sigmaaldrich.com/catalog/product/roche/11207733910?lang=es&region=ES<br>https://www.sigmaaldrich.com/catalog/product/roche/11093274910?lang=en&region=GB

Animals and other organisms

Policy information about studies involving animals; ARRIVE guidelines recommended for reporting animal research

Laboratory animals	Cloeon dipterum samples were collected from individuals kept in the laboratory for several generations (Almudi et al 2019). Genomic DNA was extracted from adult males. RNA-seq samples were obtained from the same strain at different developmental points (embryos (4 days post fertilisation (dpf), 6 dpf, 10 dpf, 14 dpf), male and female early nymphs, mid nymphs and late nymphs and male and female adults). Detailed information is provided in Supplementary Table 6.<br>Drosophila strains were obtained from VDCR Stock center. Adult female and male individuals were analysed separately. Genotypes are detailed in Supplementary table 13.

Wild animals	The study does not involve wild animals
Field-collected samples	This sutdy does not involve animals captured from the field
Ethics oversight	Not applicable.<br>The only two species used in this study are two invertebrate models, Drosophila melanogaster and Cloeon dipterum

Note that full information on the approval of the study protocol must also be provided in the manuscript.
