# Supplementary material for: Genomic adaptations to aquatic and aerial life in mayflies and the origin of insect wings
Source: Nat Commun. 2020 May 26;11:2631. doi: 10.1038/s41467-020-16284-8 (PMC7250882; doi:10.1038/s41467-020-16284-8)
Supplement: Supplementary file 4 — Description of Additional Supplementary Files [file 41467_2020_16284_MOESM4_ESM.pdf]

Description of Additional Supplementary Files

File Name: Supplementary Data 1
Description: WGCNA modules

File Name: Supplementary Data 2
Description: Sequencing table statistics

File Name: Supplementary Data 3
Description: Kraken contaminats in mayfly genome assembly

File Name: Supplementary Data 4
Description: Contaminants

File Name: Supplementary Data 5
Description: Orthofinder species

File Name: Supplementary Data 6
Description: Orthofinder stats orthologues

File Name: Supplementary Data 7
Description: RNA samples sequenced

File Name: Supplementary Data 8
Description: Mfuzz stages

File Name: Supplementary Data 9
Description: Mfuzz data

File Name: Supplementary Data 10
Description: Chemosensory complement in insects

File Name: Supplementary Data 11
Description: tau indexes

File Name: Supplementary Data 12
Description: DESeq results female vs male heads

File Name: Supplementary Data 13
Description: WGCNA samples, genes and enriched GO terms per species

File Name: Supplementary Data 14
Description: Shared genes between *C. dipterum* and *D. melanogaster* wing pad and wing disc WGCNA modules

File Name: Supplementary Data 15
Description: RNAi lines used in *D. melanogaster*

File Name: Supplementary Data 16
Description: Supplementary Table 15: Genes highly expressed in C. dipterum wings and gills

File Name: Supplementary Data 17
Description: Supplementary Table 16: Primers used to design RNA probes for in situ hybridization

File Name: Supplementary Data 18
Description: Supplementary Table 17: Opsins identified in Ephemeroptera
