# Supplementary material for: Genomic adaptations to aquatic and aerial life in mayflies and the origin of insect wings
Source: Nat Commun. 2020 May 26;11:2631. doi: 10.1038/s41467-020-16284-8 (PMC7250882; doi:10.1038/s41467-020-16284-8)

WGCNA module annotation

1. *C. dipterum*, *D. melanogaster* and *S. maritima* module annotations
Module colour and annotated names……………………………………………………page 2

2. *C. dipterum* module RNA-seq expression and GO terms………………………………..page 3

3. *D. melanogaster* module RNA-seq expression and GO terms………………………page 14

4. *S. maritima* module RNA-seq expression and GO terms……………………………...page 23

C. dipterum WGCNA modules annotation		*D. melanogaster* WGCNA modules annotation		*S.maritima* WGCNA modules annotation	
Module Colour	Module annotated name	Module Colour	Module annotated name	Module Colour	Module annotated name
grey60	Vesicle transport	darkgrey	Gut	lightcyan	Head
greenyellow	Brain	darkturquoise	Larval CNS	saddlebrown	Adult male
sienna3	Cytoskeleton	salmon	Jump Muscle	greenyellow	Neurogenesis
lightgreen	Pre-nymph	red	Salivary glands	red	Fat body
yellow	Wing	blue	Brain- Synapsis	darkgreen	membrane
steelblue	Malpighian tube	greenyellow	Wing Disc	skyblue	Embryo/Nerve cord
brown	Gills	cyan	12-14 hpf embryo	orange	Protein synthesis
orange	Cuticle	black	Flight muscle	black	Nerve cord- Synapsis
blue	Muscle	magenta	4-6 h embryo- Transcription	midnightblue	Splicing
skyblue	Chitin	purple	Adult heads- visual perception	blue	Gut
yellowgreen	Trachea	green	Ovaries	grey60	Malpighian tubes
black	Gut	lightcyan	Fat body	purple	Fat body B- Translation
paleturquoise	Gut/Malpighian	white	Muscle- Cell metabolism	white	Proteasome
cyan	Protein synthesis	brown	Testis	lightgreen	Cellular respiration
tan	10 dpf embryo- Neurogenesis	pink	Malpighian tubule	magenta	Ovaries-Replication
darkolivegreen	Brain B- Lipid metabolism	skyblue	Adult gut	lightyellow	Fatty acid metabolism
magenta	Male Head- Phototransduction	grey60	Larval gut	royalblue	Cellular metabolism
turquoise	Ovaries			darkorange	Fat body- Post-translation modification
darkmagenta	Female Head- Fatty acid metabolism			brown	Embryogenesis
red	4 dpf embryo- Autophagy			turquoise	Testis-Cilium
saddlebrown	Embryogenesis- Synapsis			darkgrey	Transcription
green	Testis				

C. dipterum modules

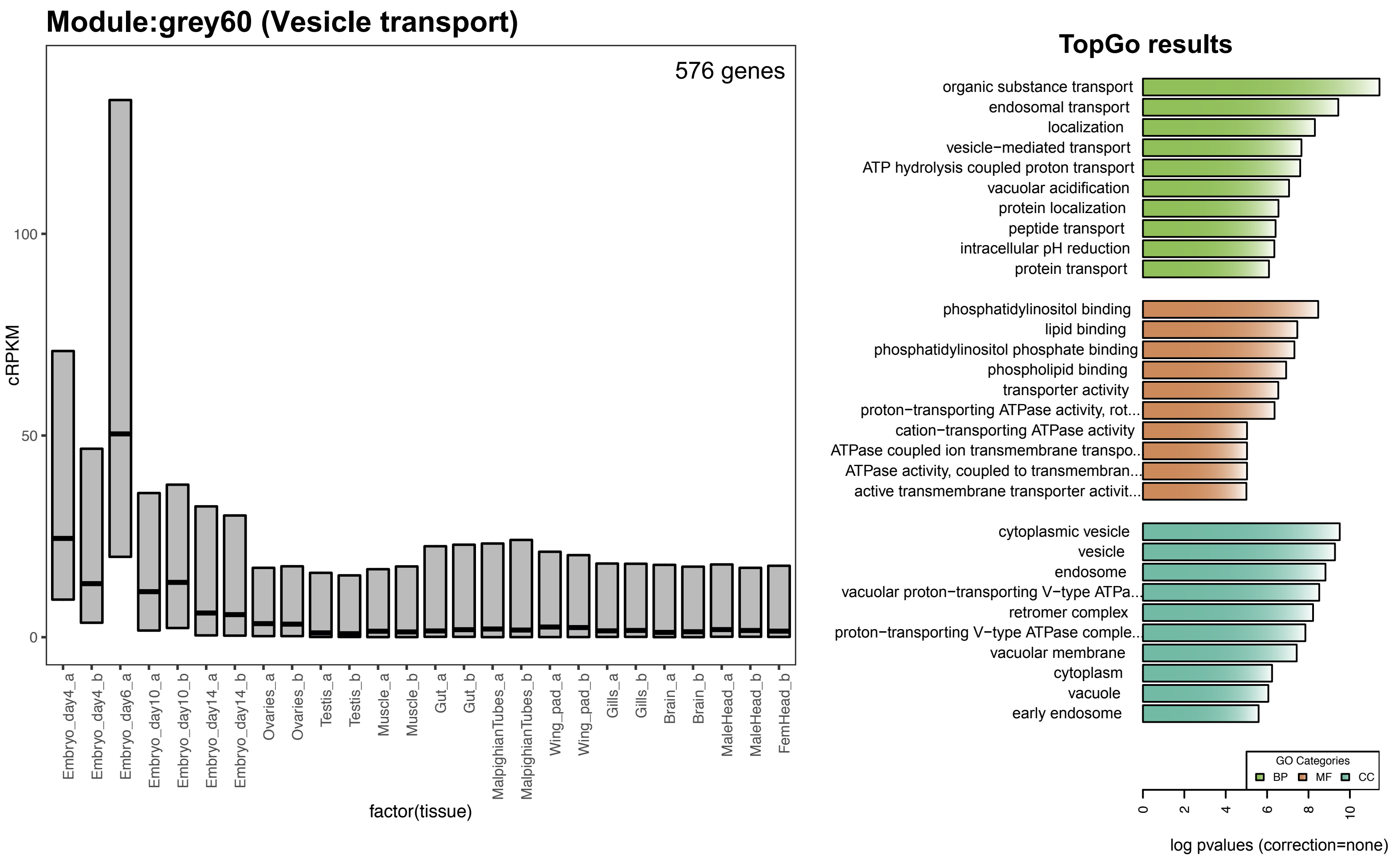

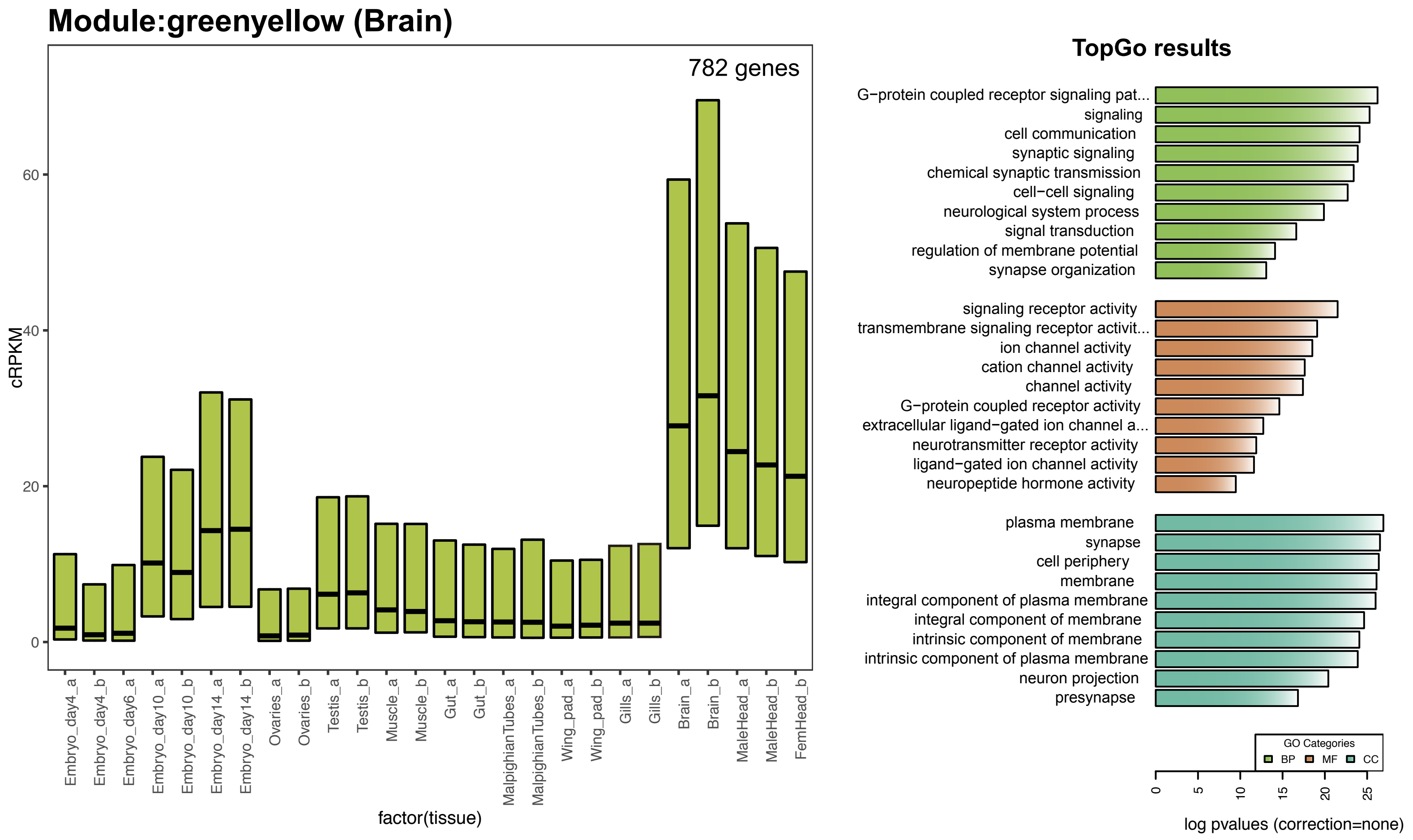

C. dipterum modules

Module:sienna3 (Cytoskeleton)

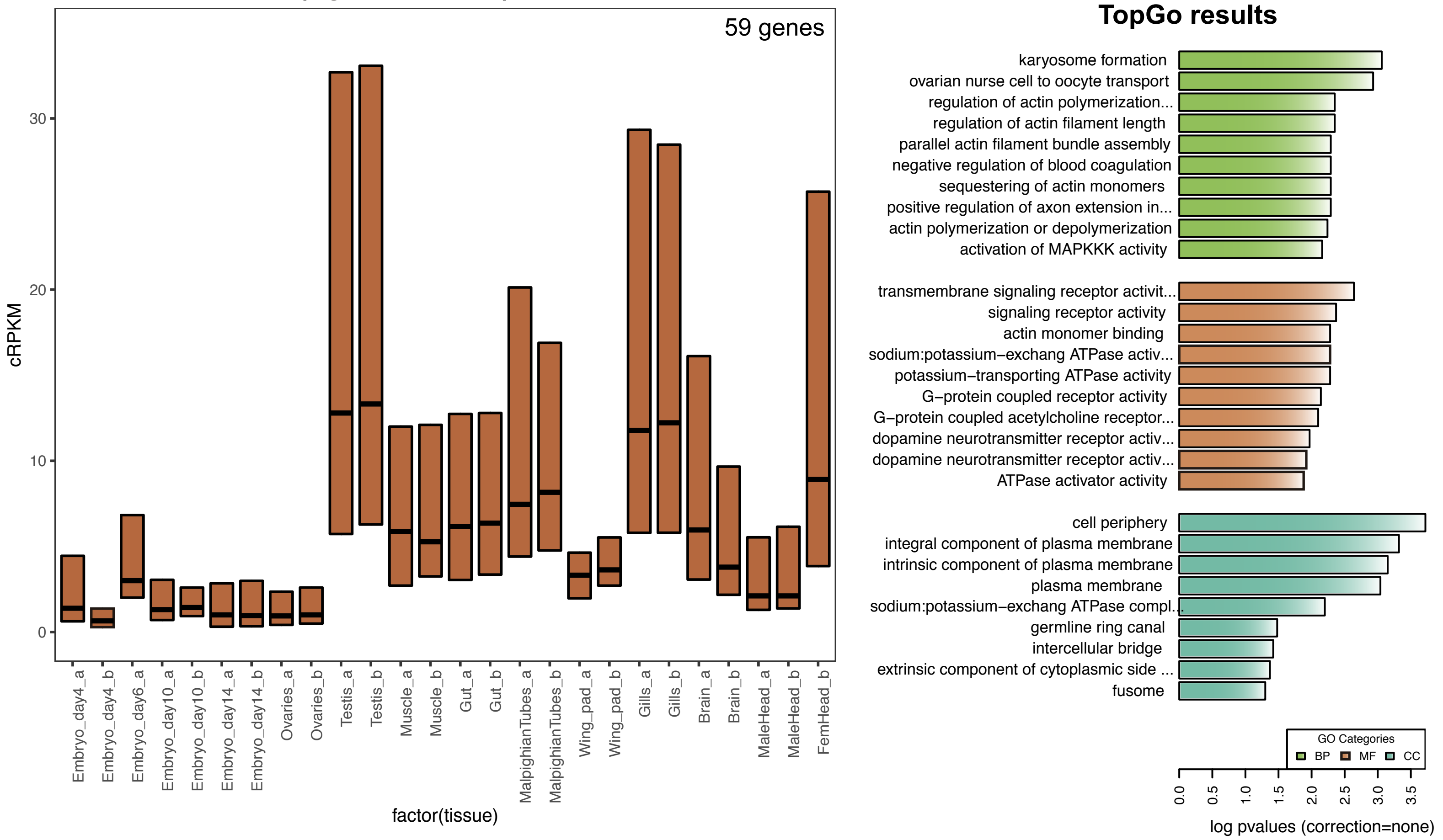

Module:lightgreen (Pre-nymph)

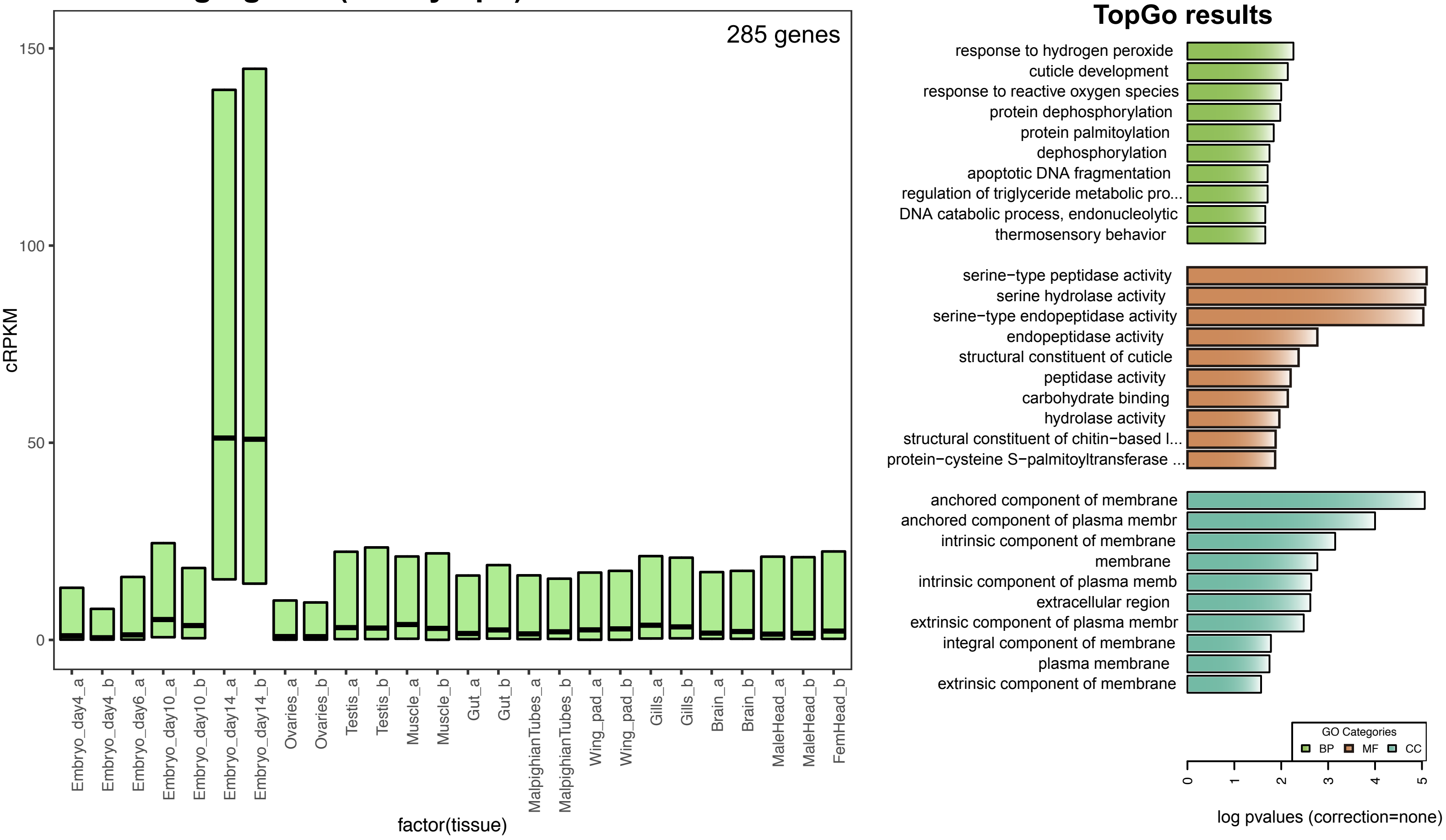

C. dipterum modules

Module:yellow (Wing)

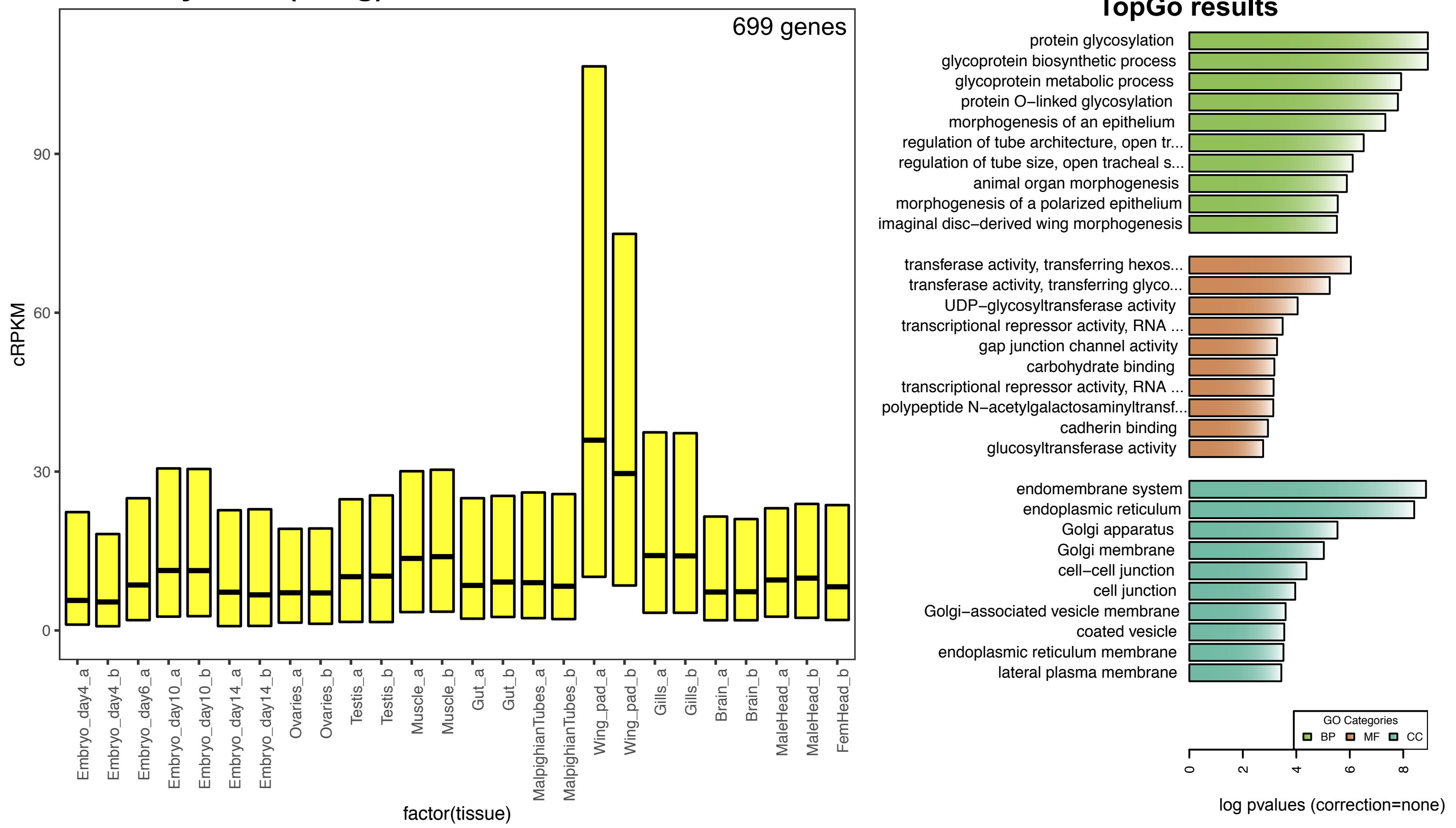

Module:steelblue (Malpighian tube)

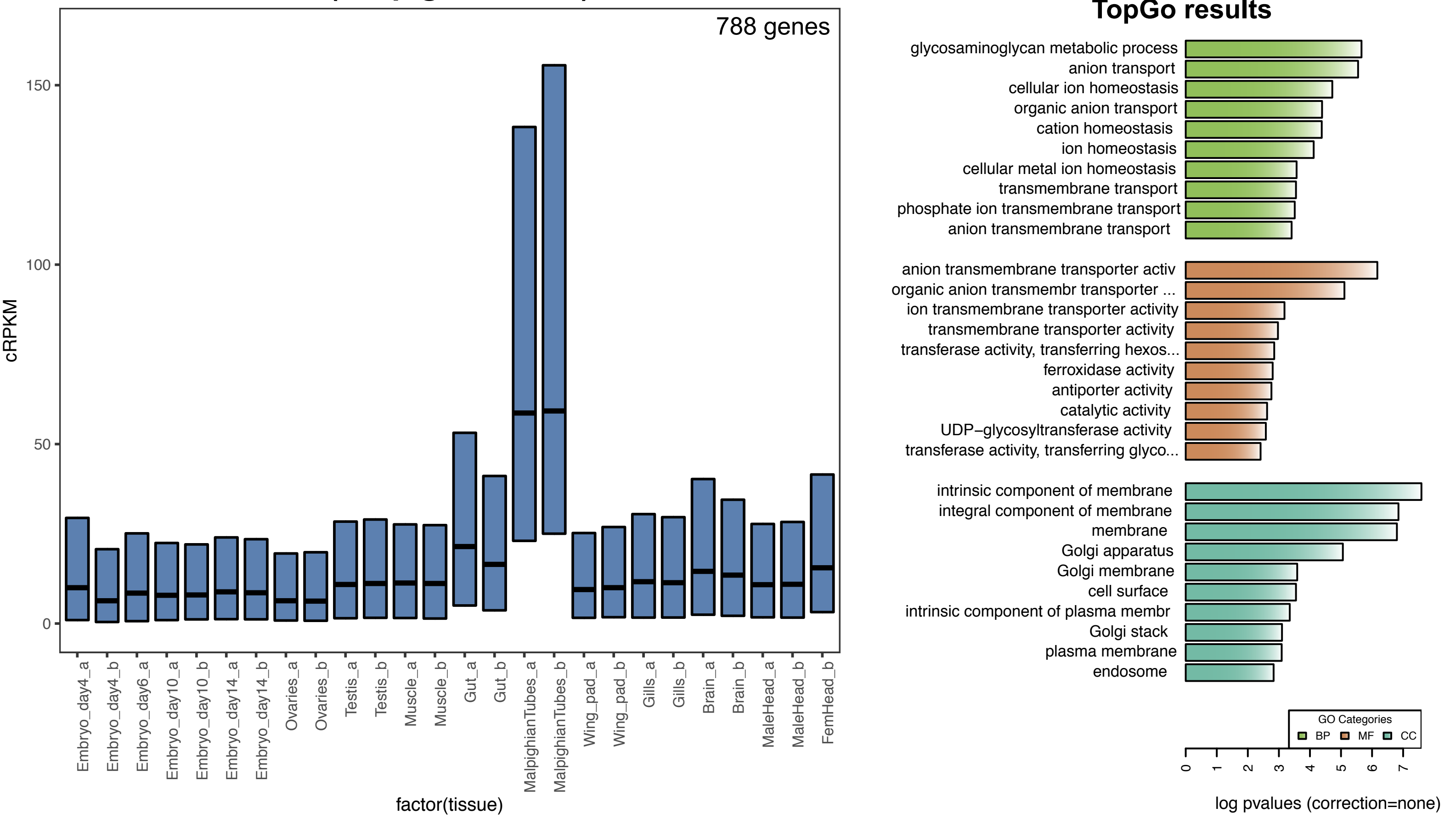

C. dipterum modules

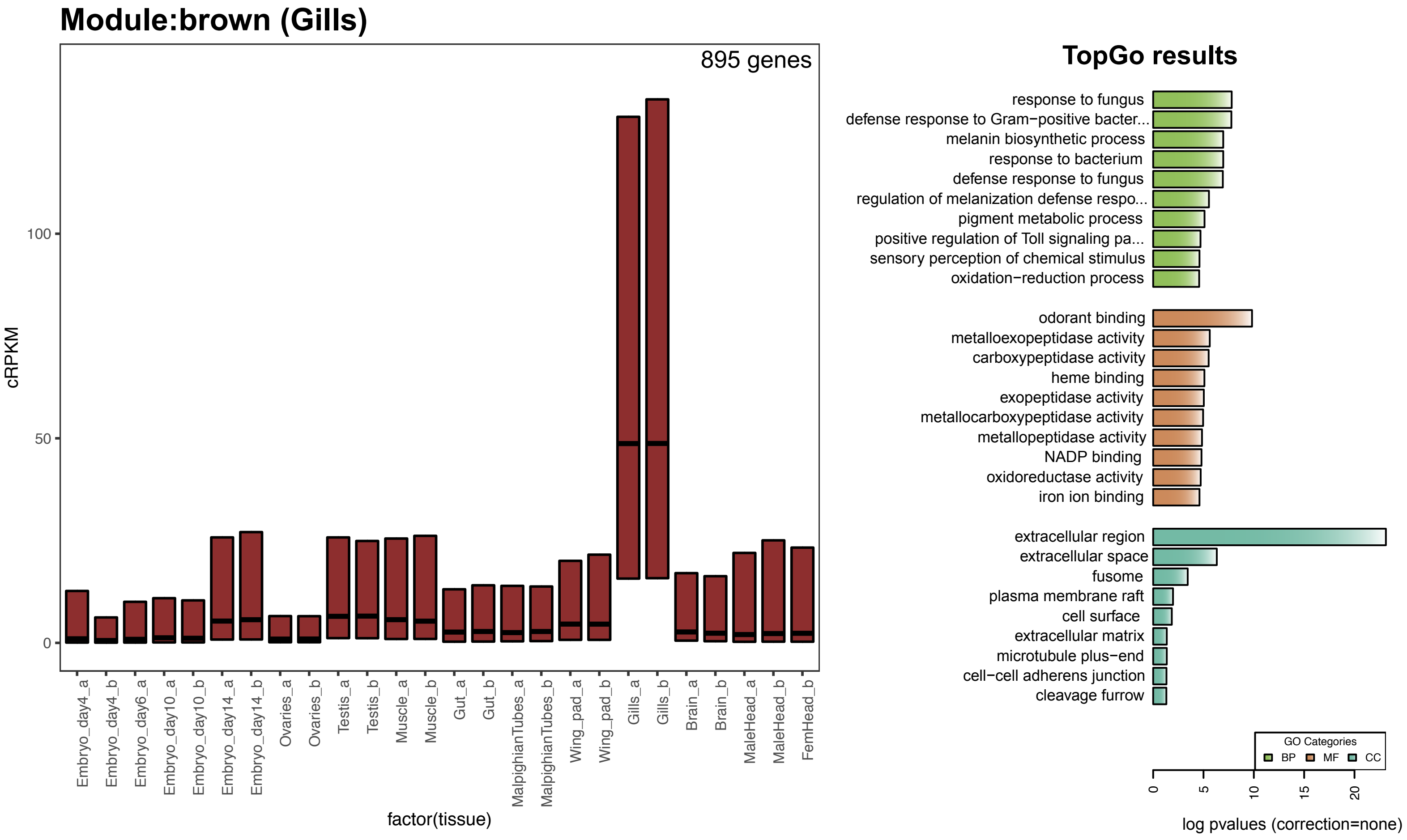

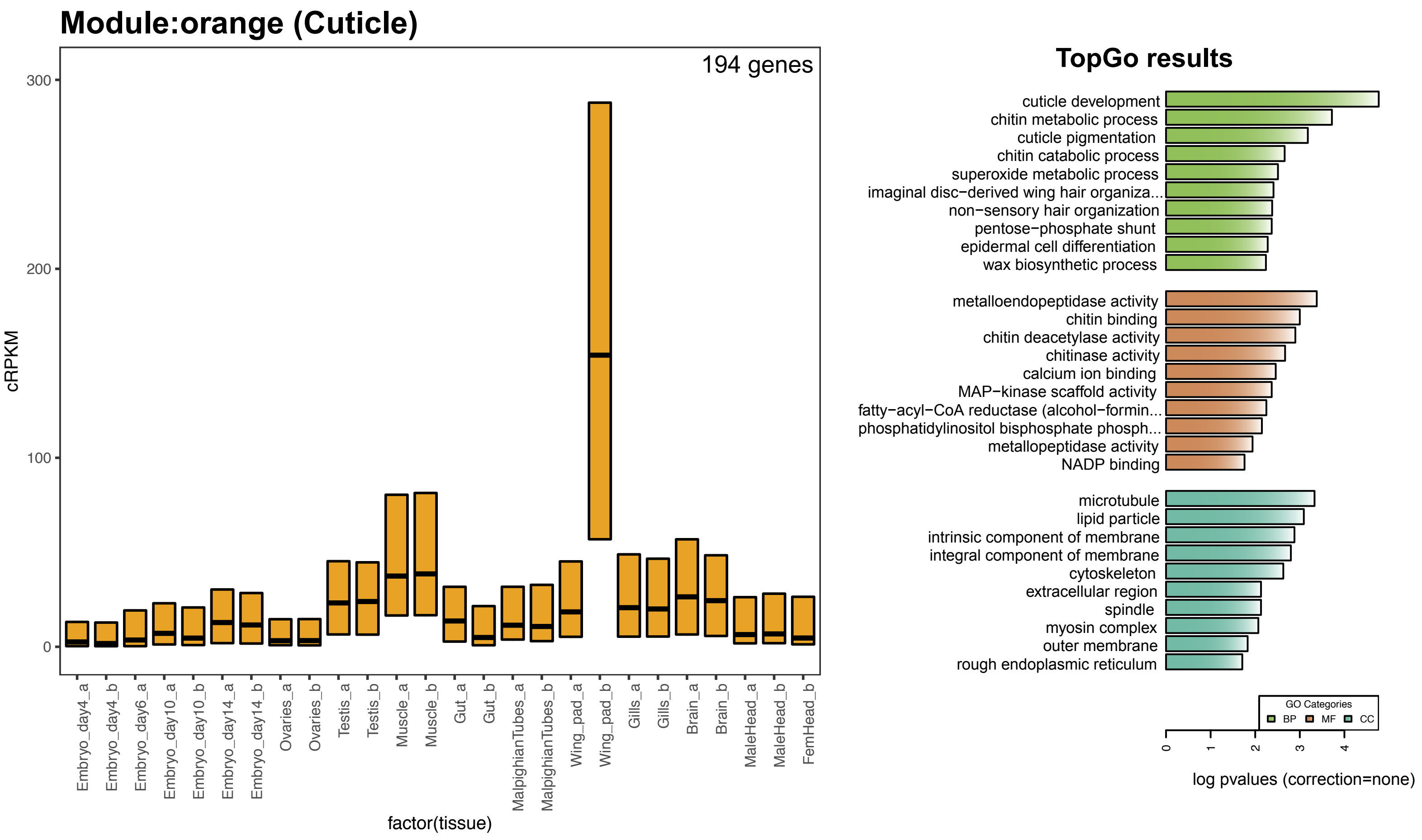

C. dipterum modules

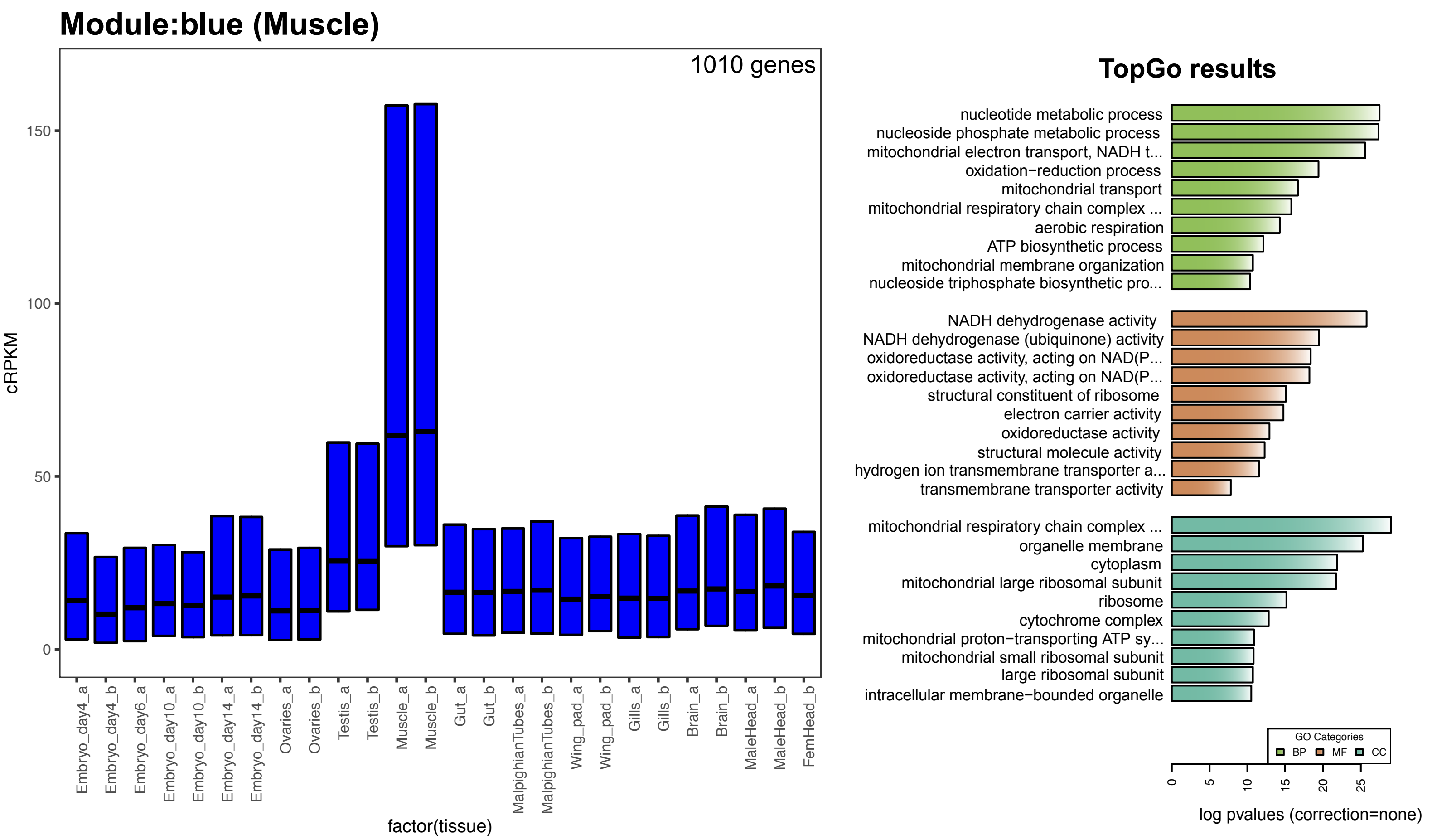

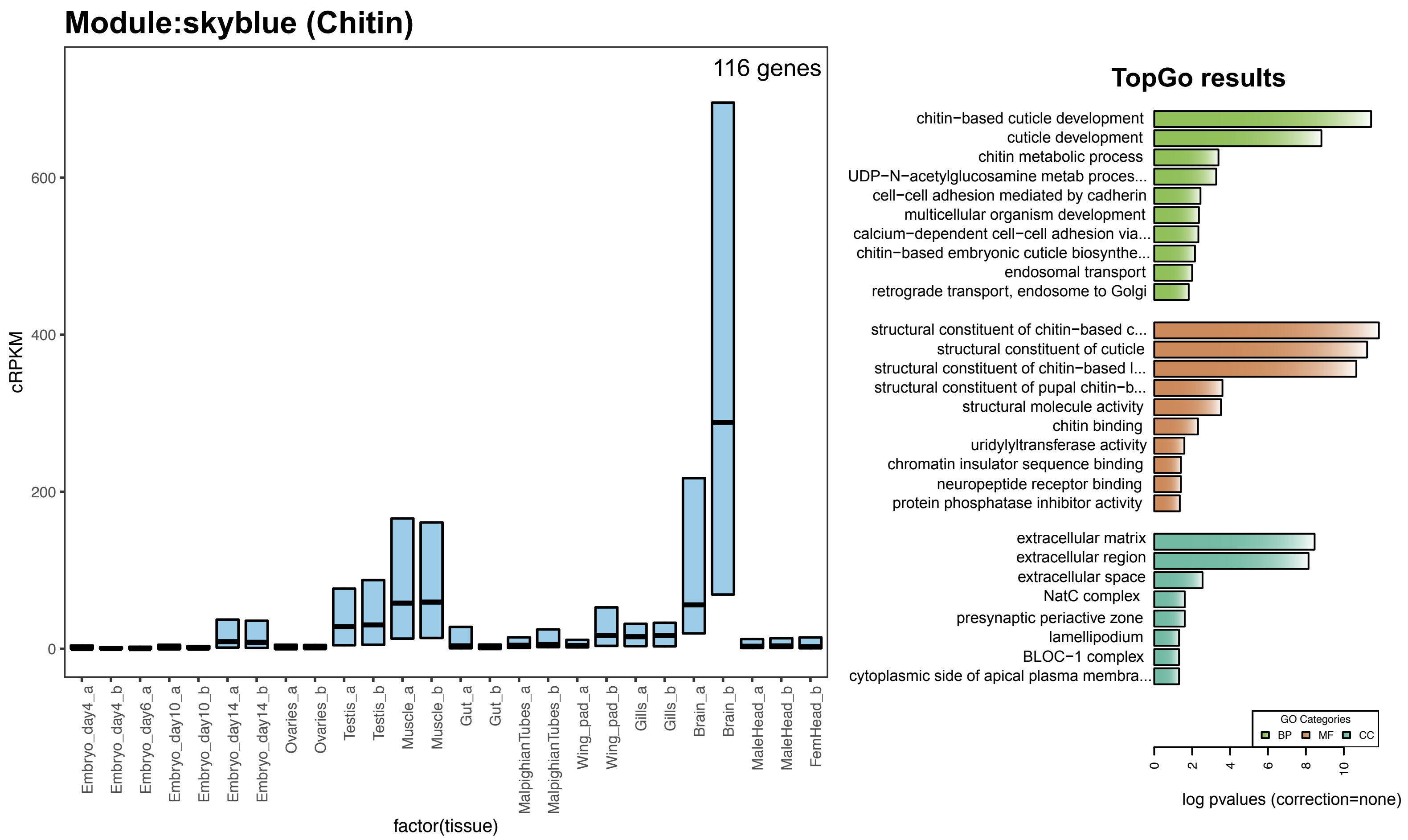

C. dipterum modules

Module:yellowgreen (Trachea)

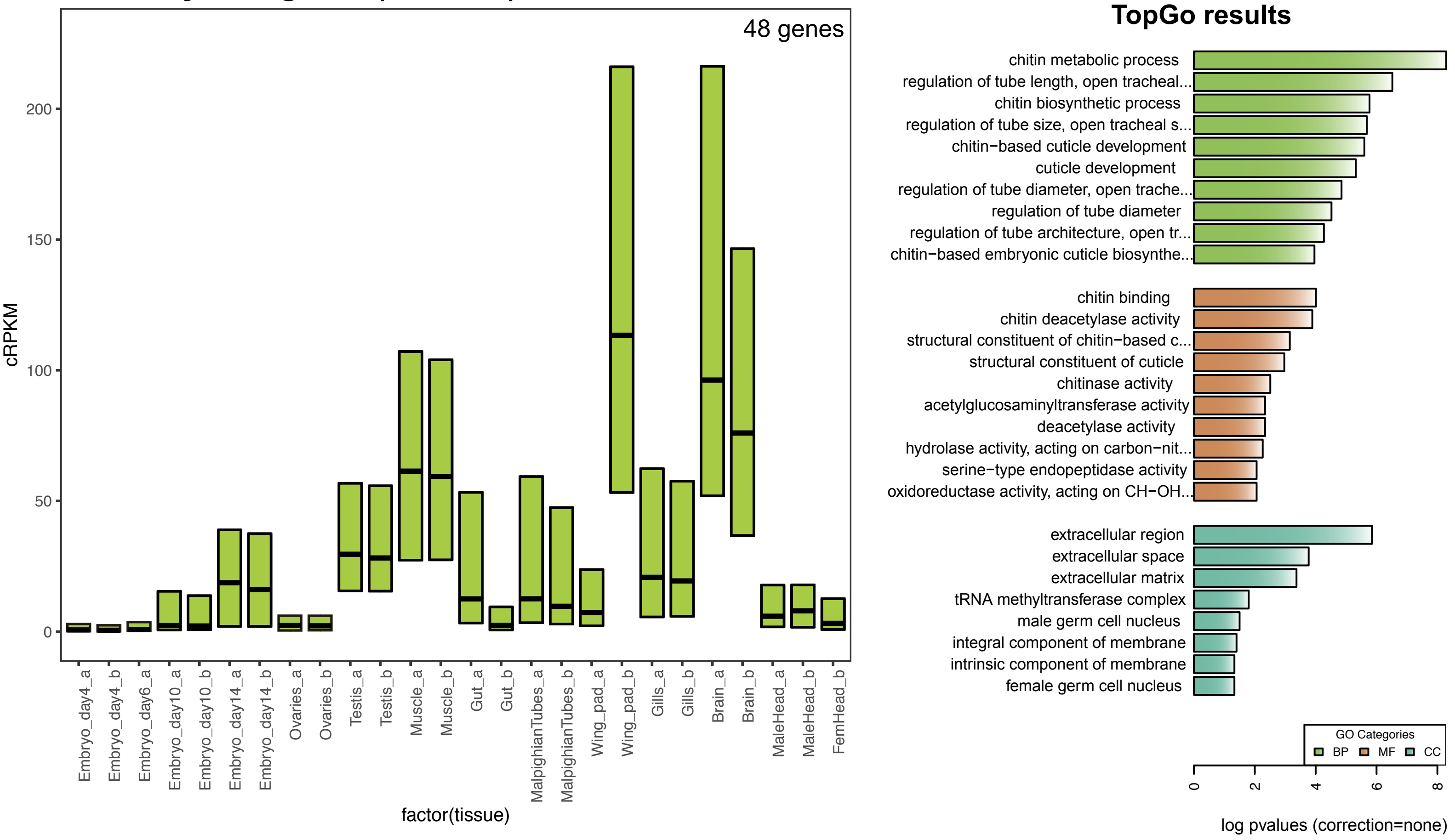

Module:black (Gut)

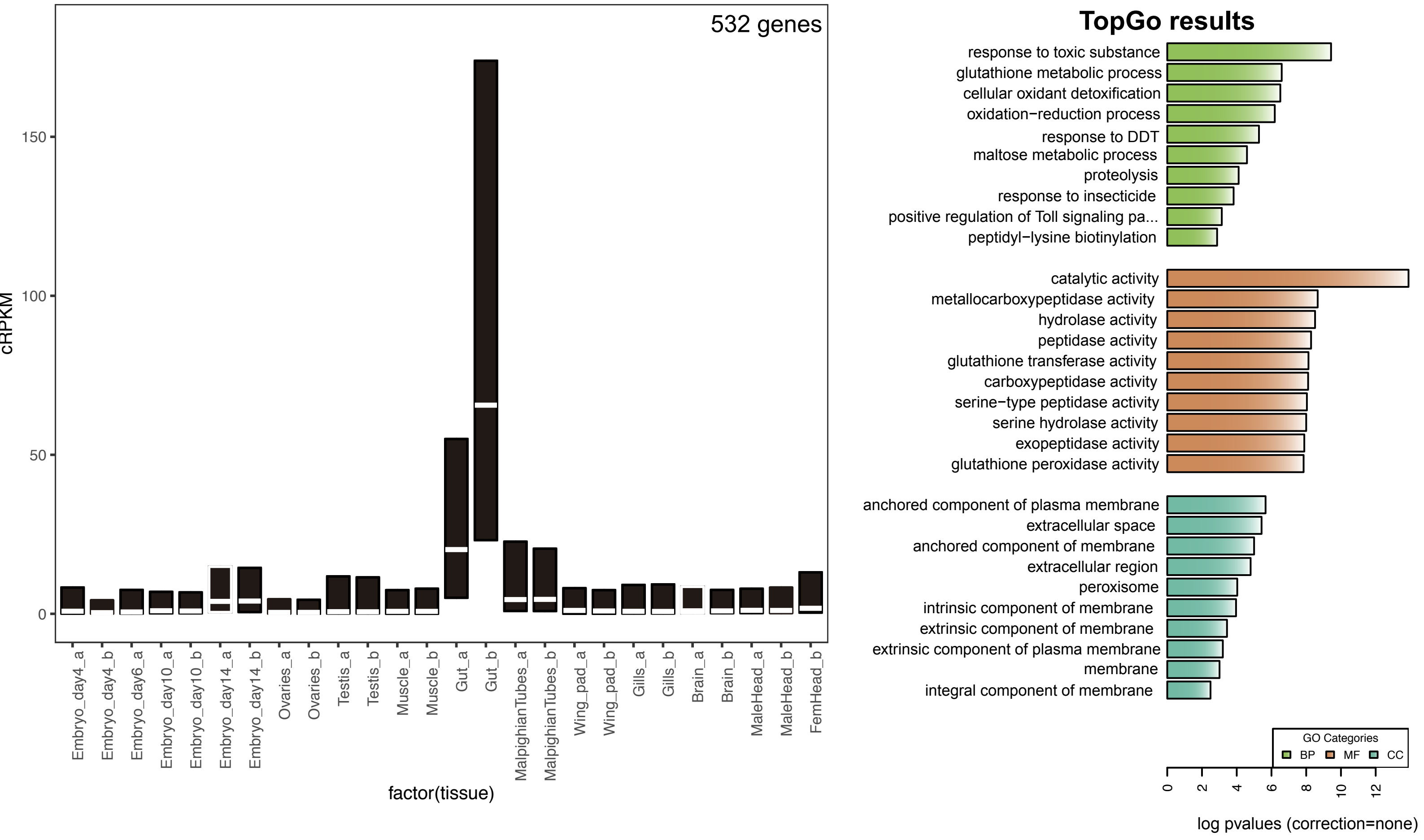

C. dipterum modules

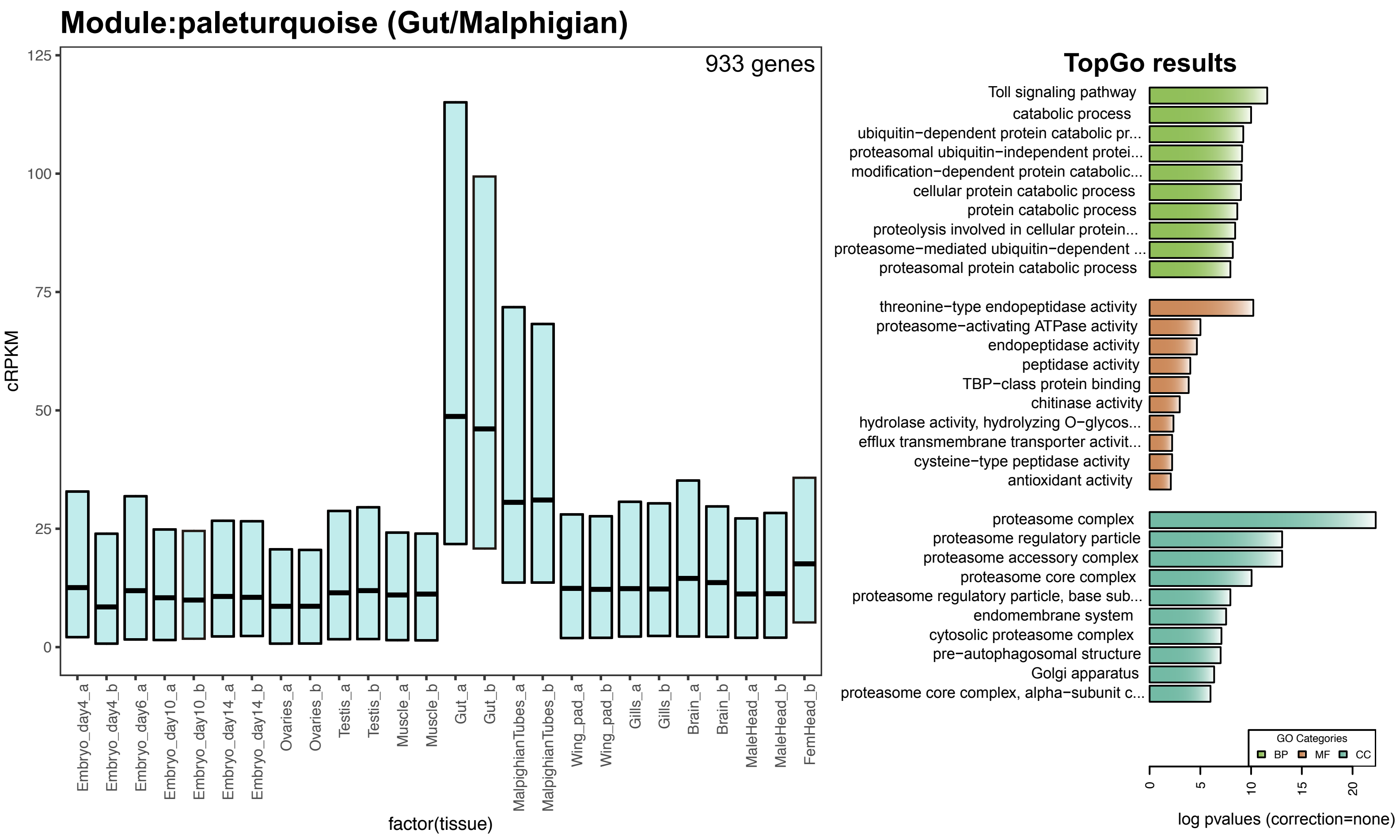

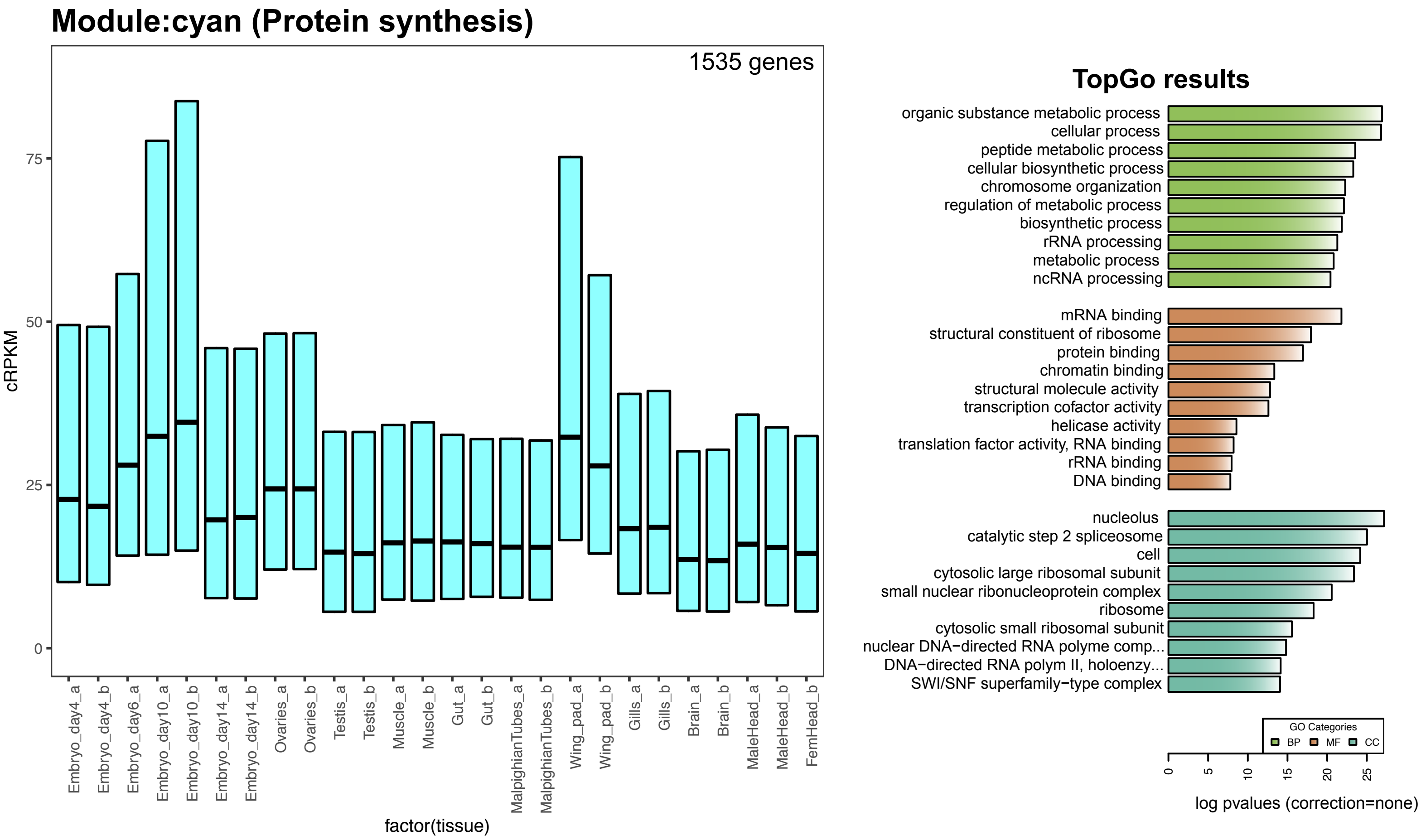

C. dipterum modules

Module:tan (Day 10 embryo- Neurogenesis)

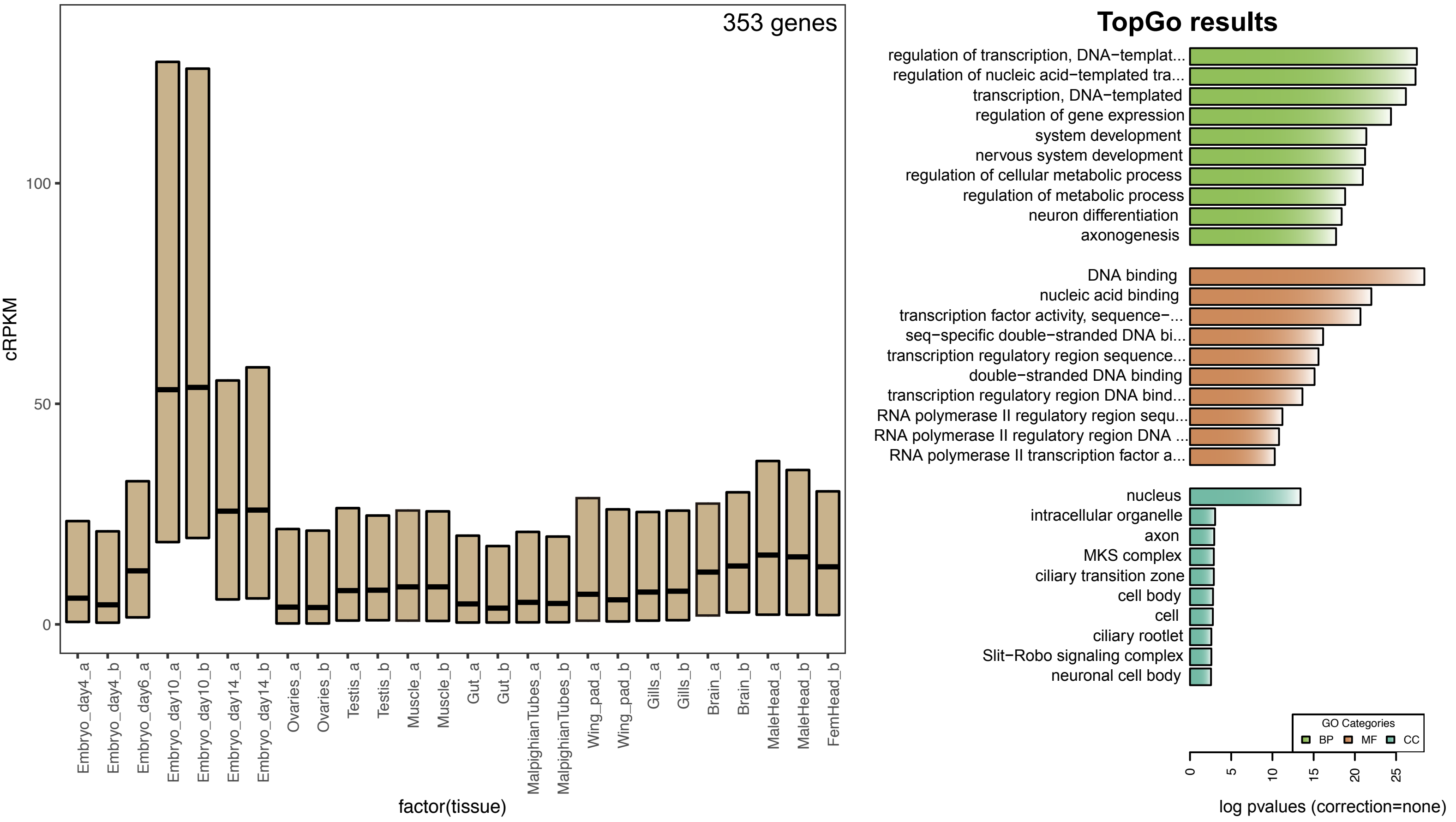

Module:darkolivegreen (Brain B- Lipid metabolism)

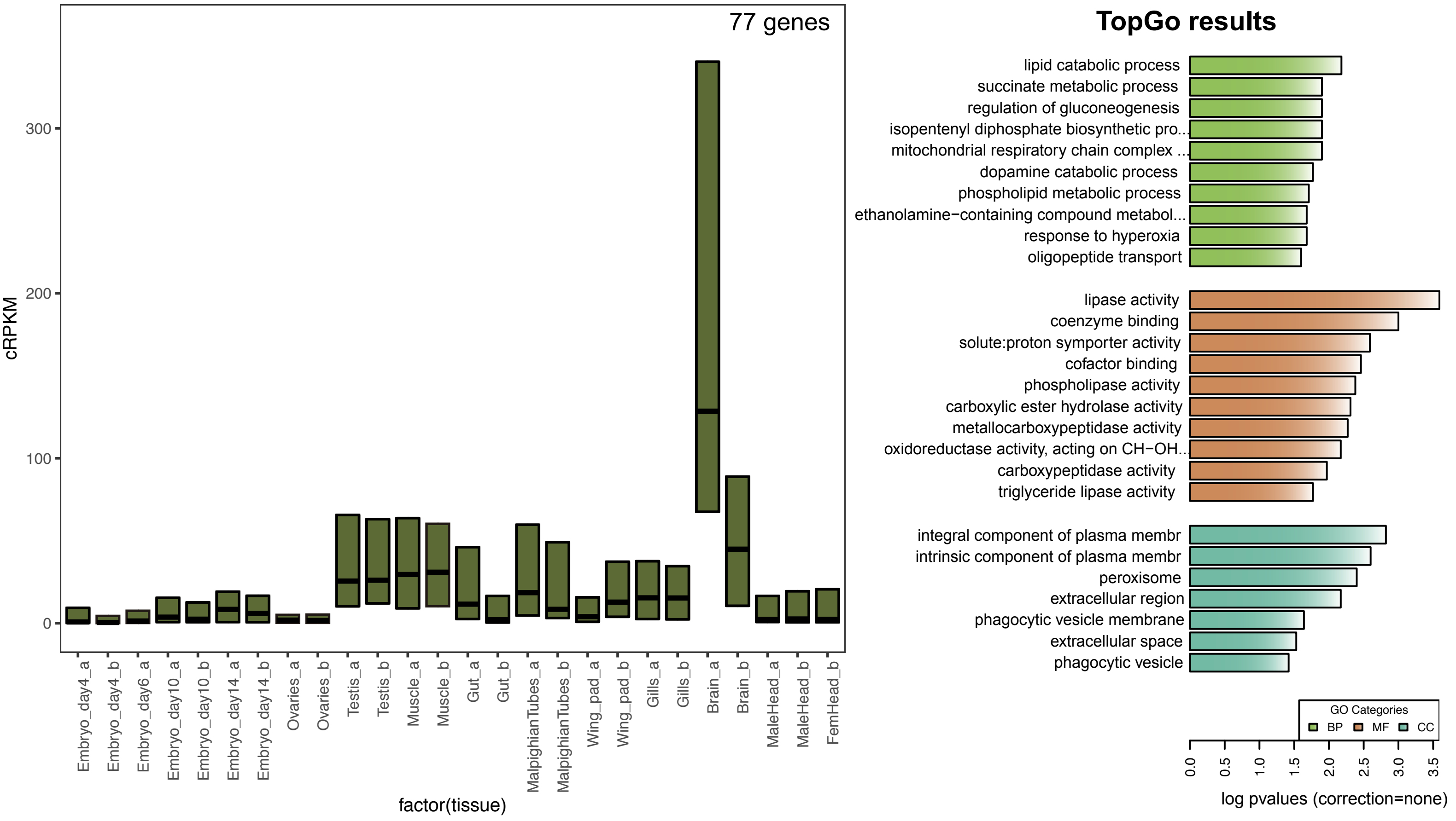

C. dipterum modules

Module:magenta (Male Head- Phototransduction)

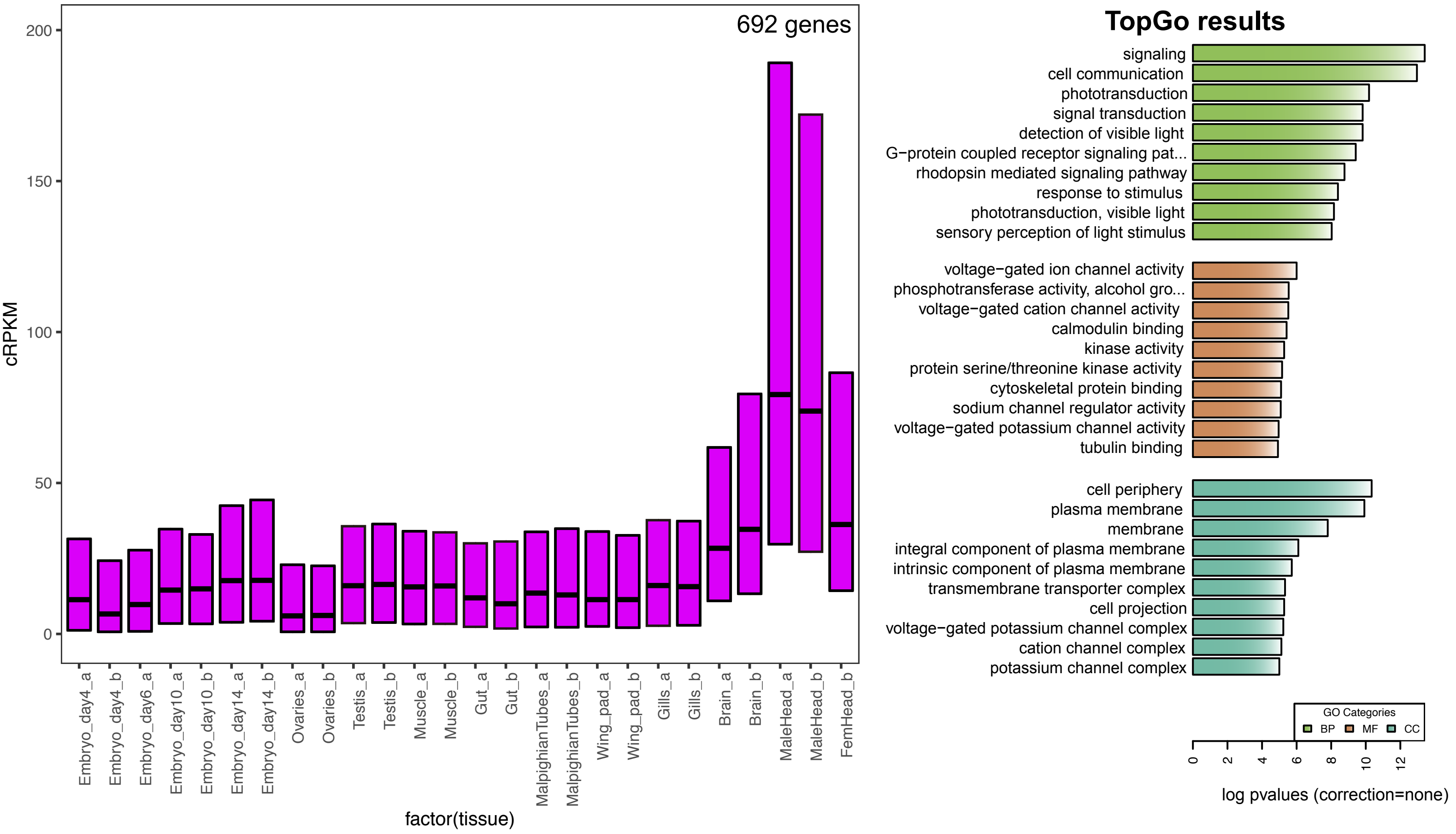

Module:turquoise (Ovaries)

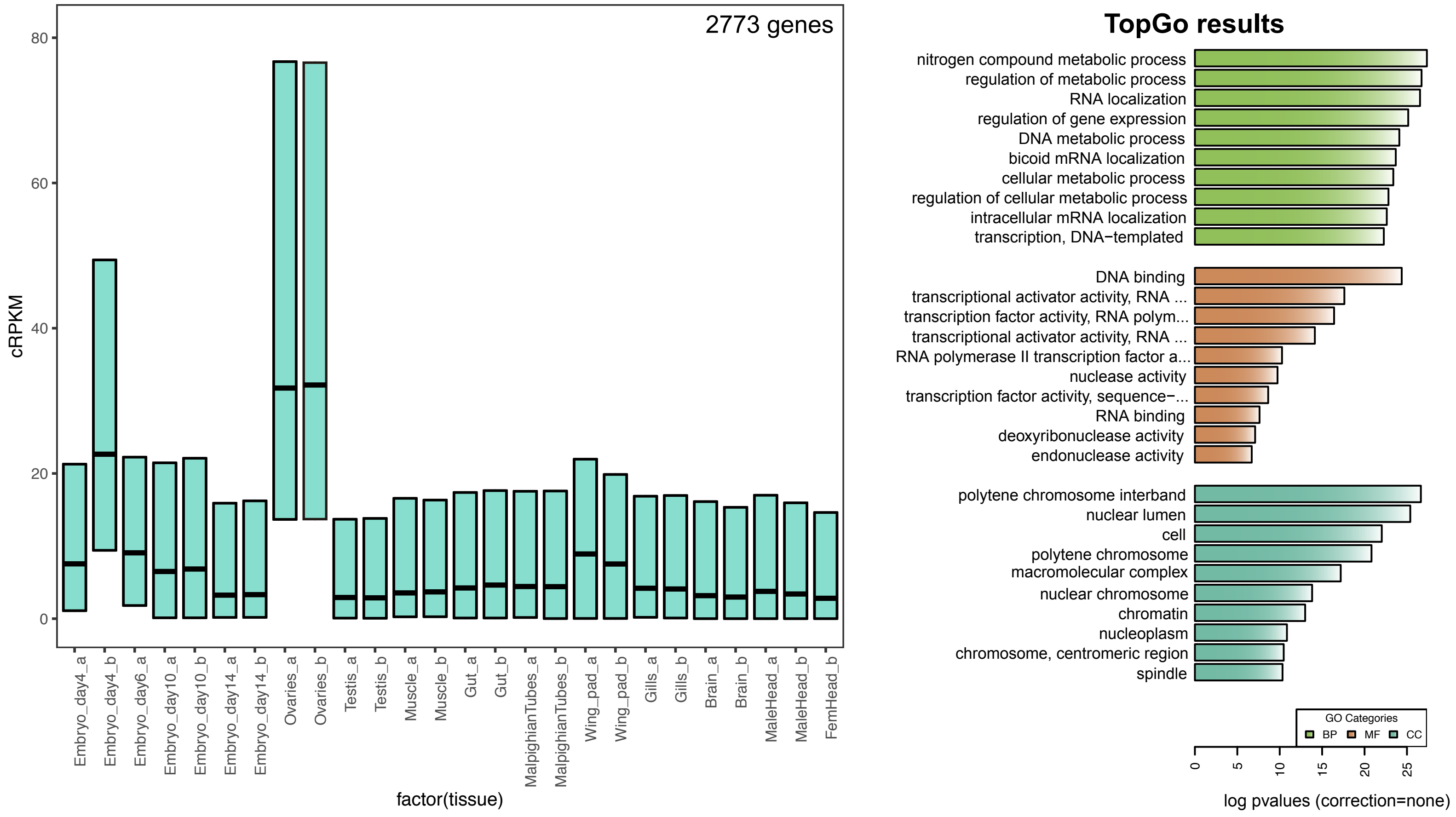

C. dipterum modules

Module:darkmagenta (Female Head- Fatty acid metab)

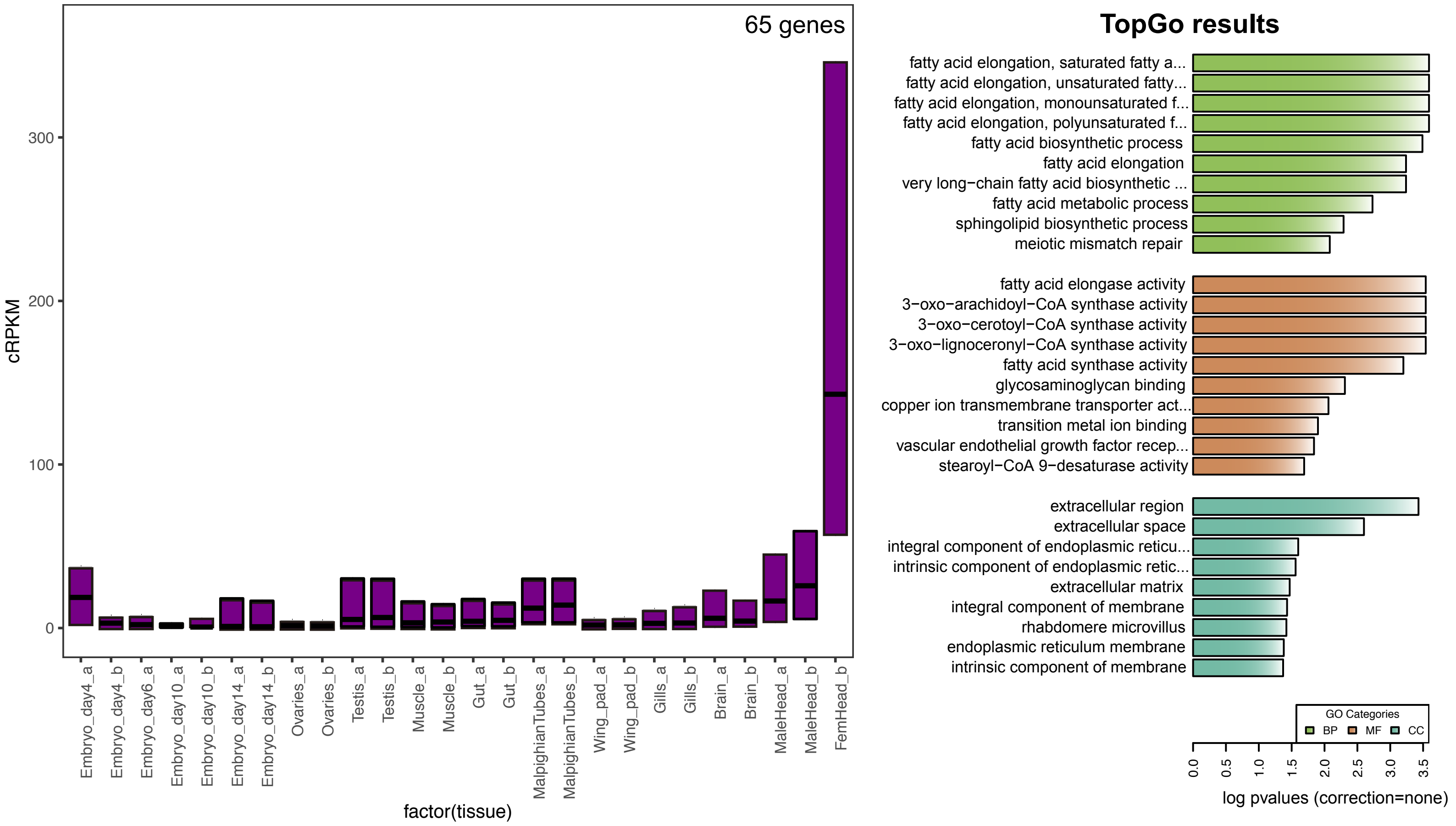

Module:red (Embryo day 4- Autophagy)

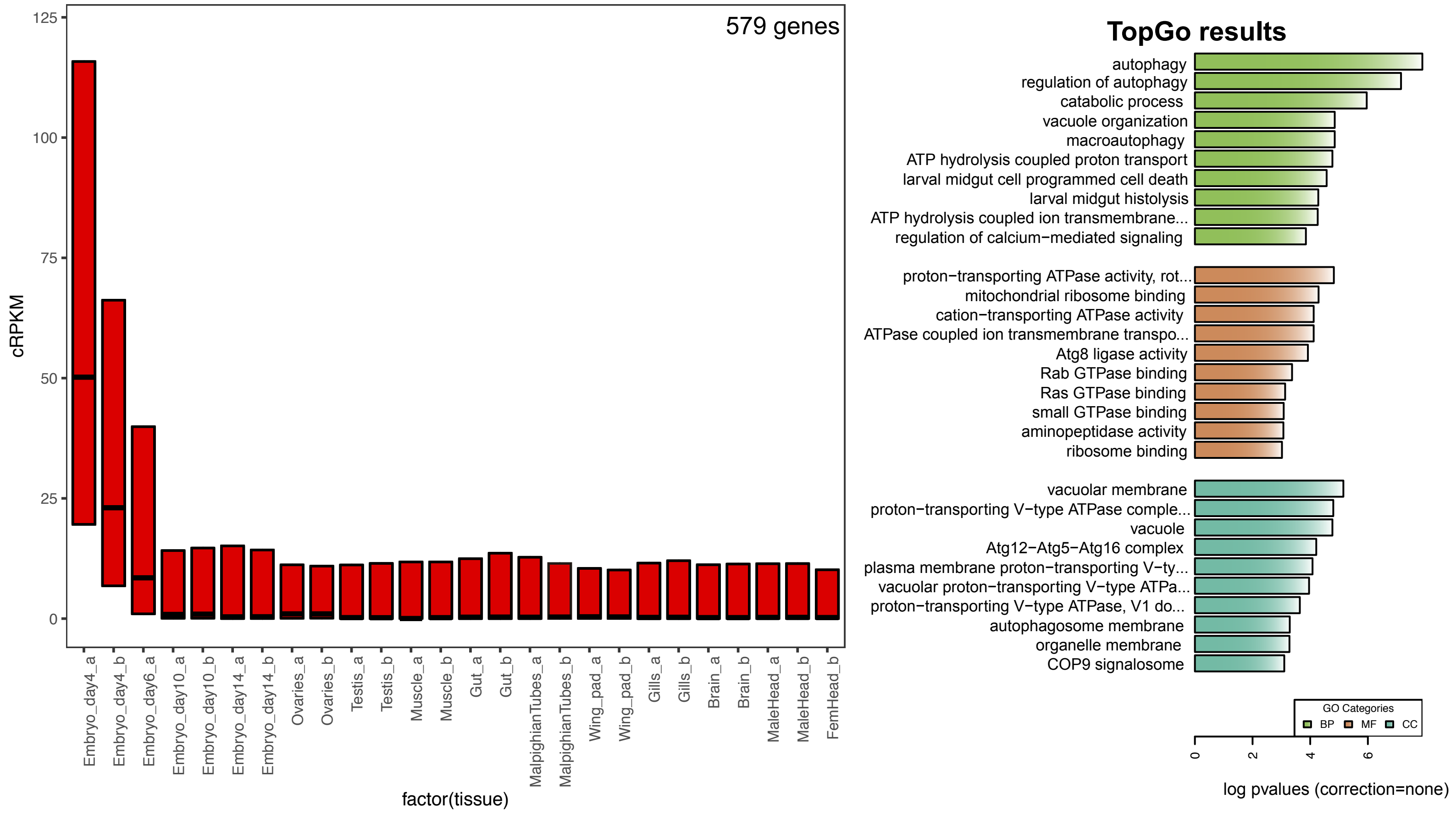

C. dipterum modules

Module:saddlebrown (Embryogenesis- Synapsis)

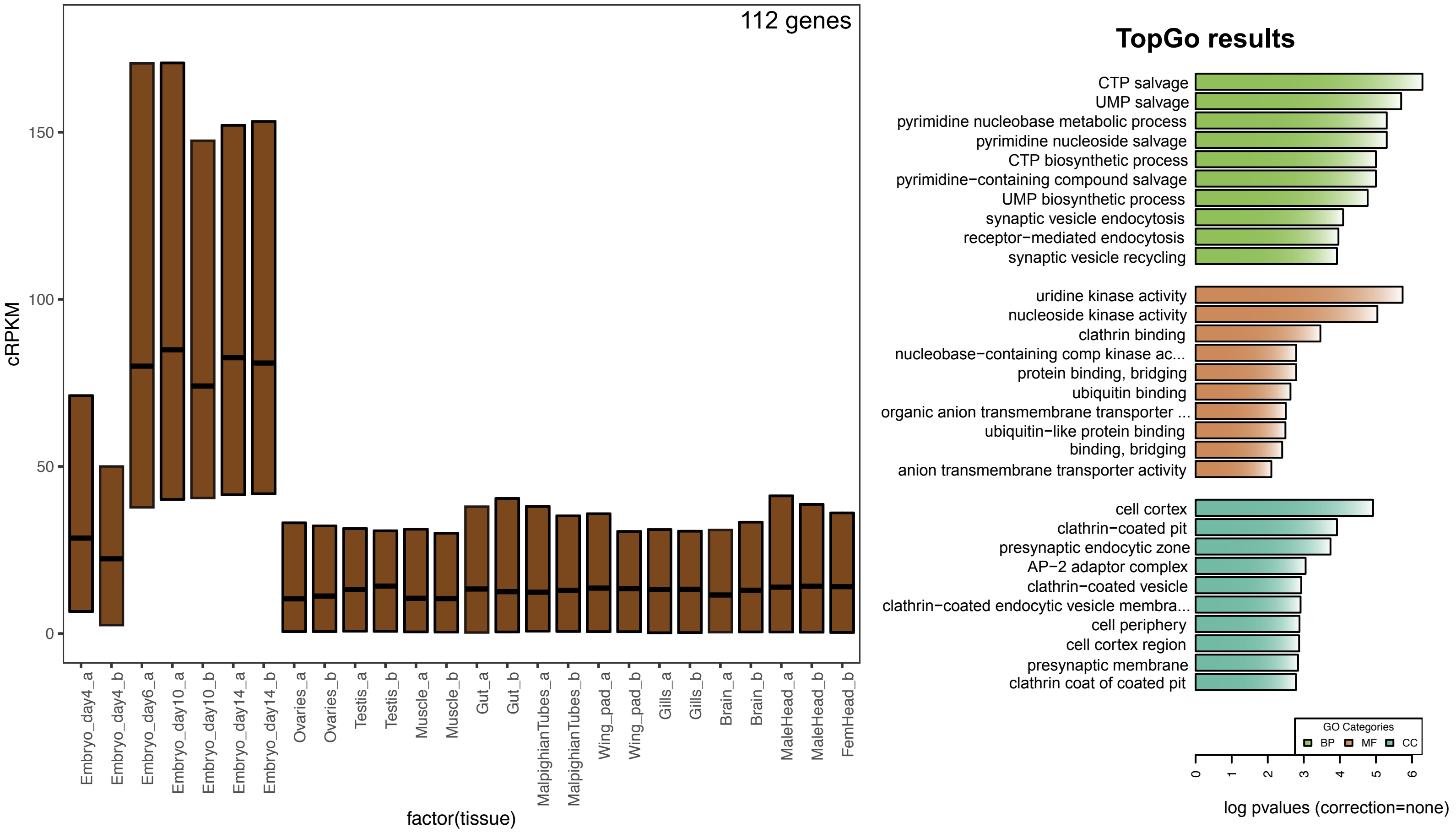

Module:green (Testis)

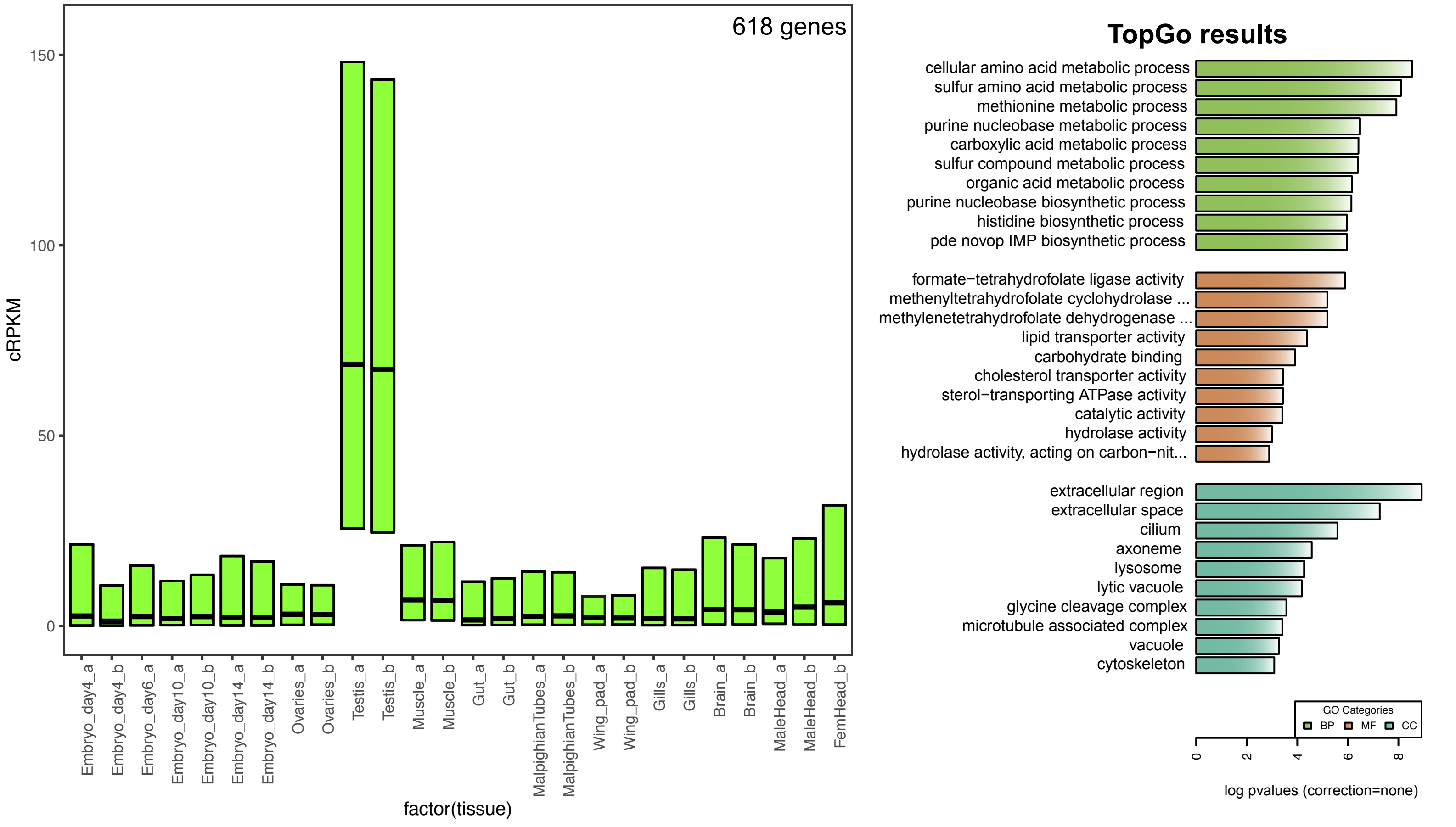

D. melanogaster modules

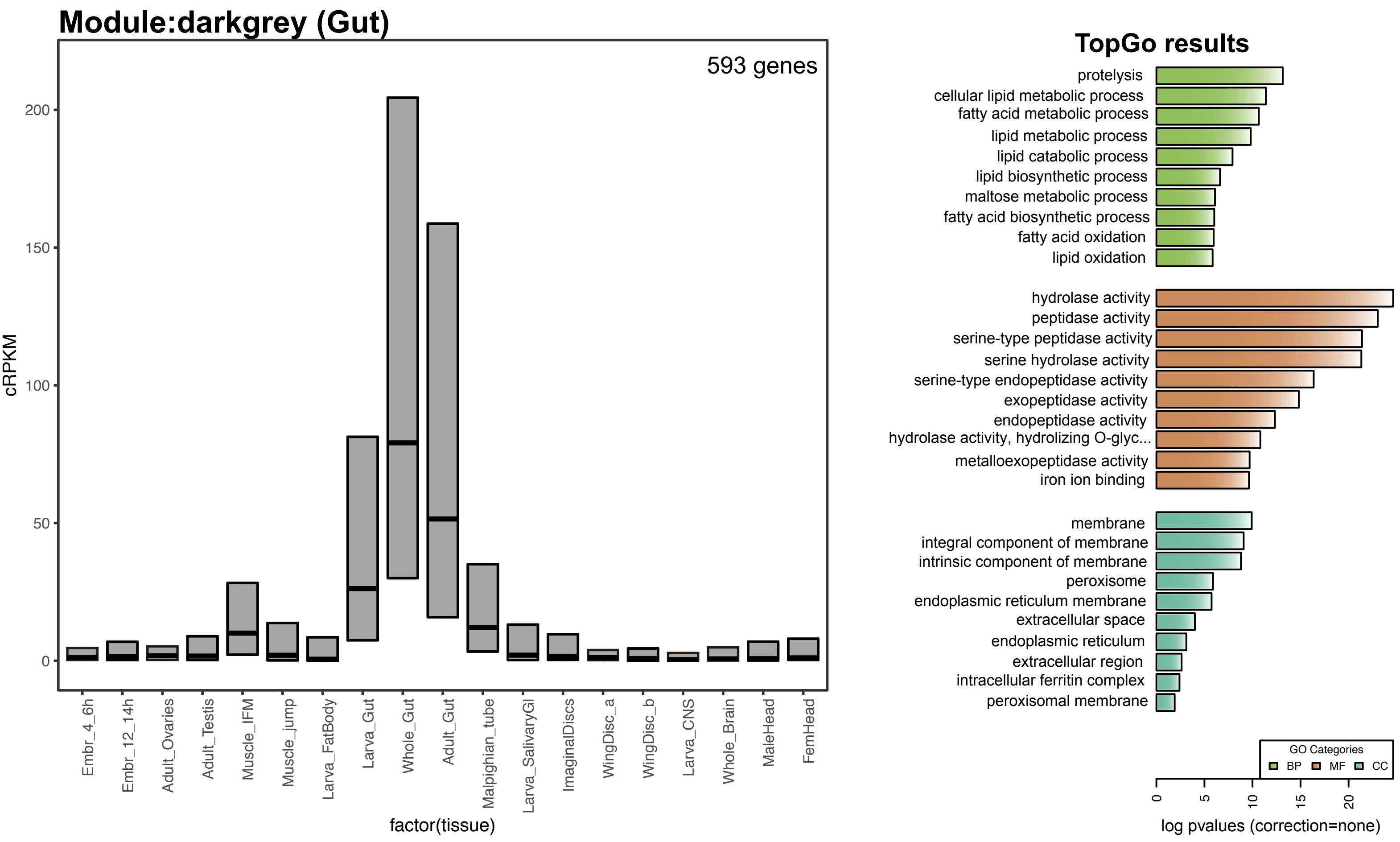

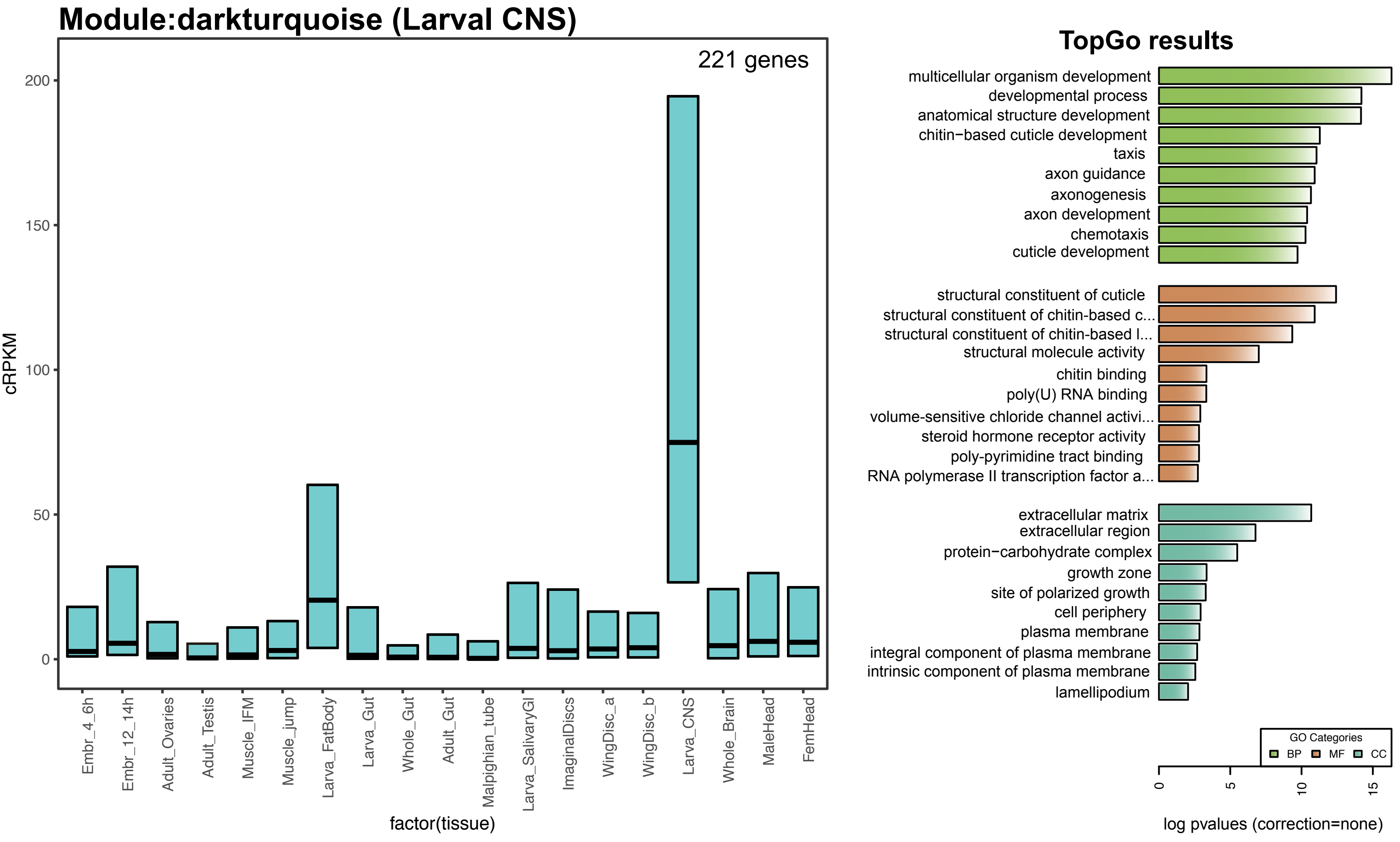

D. melanogaster modules

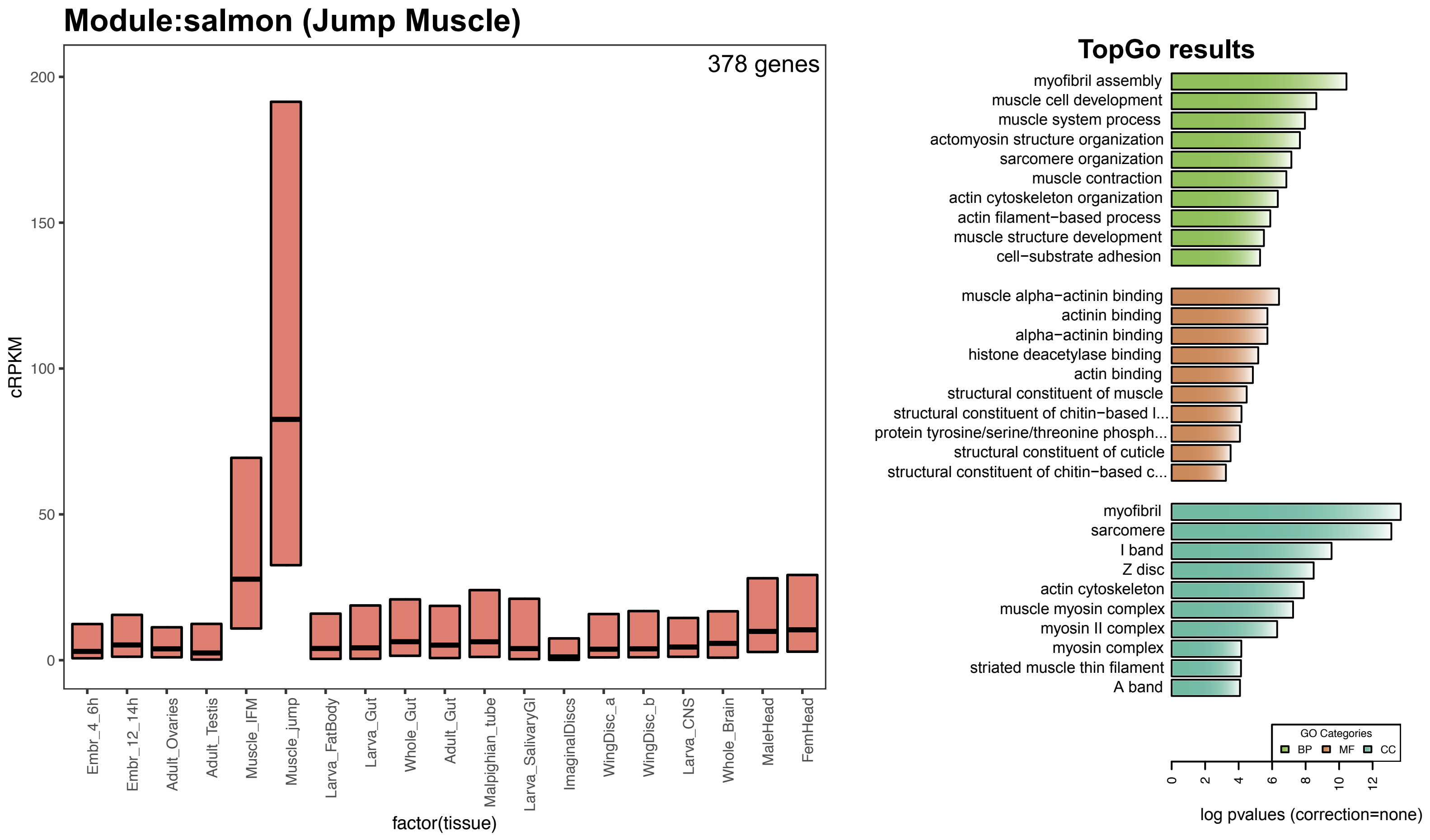

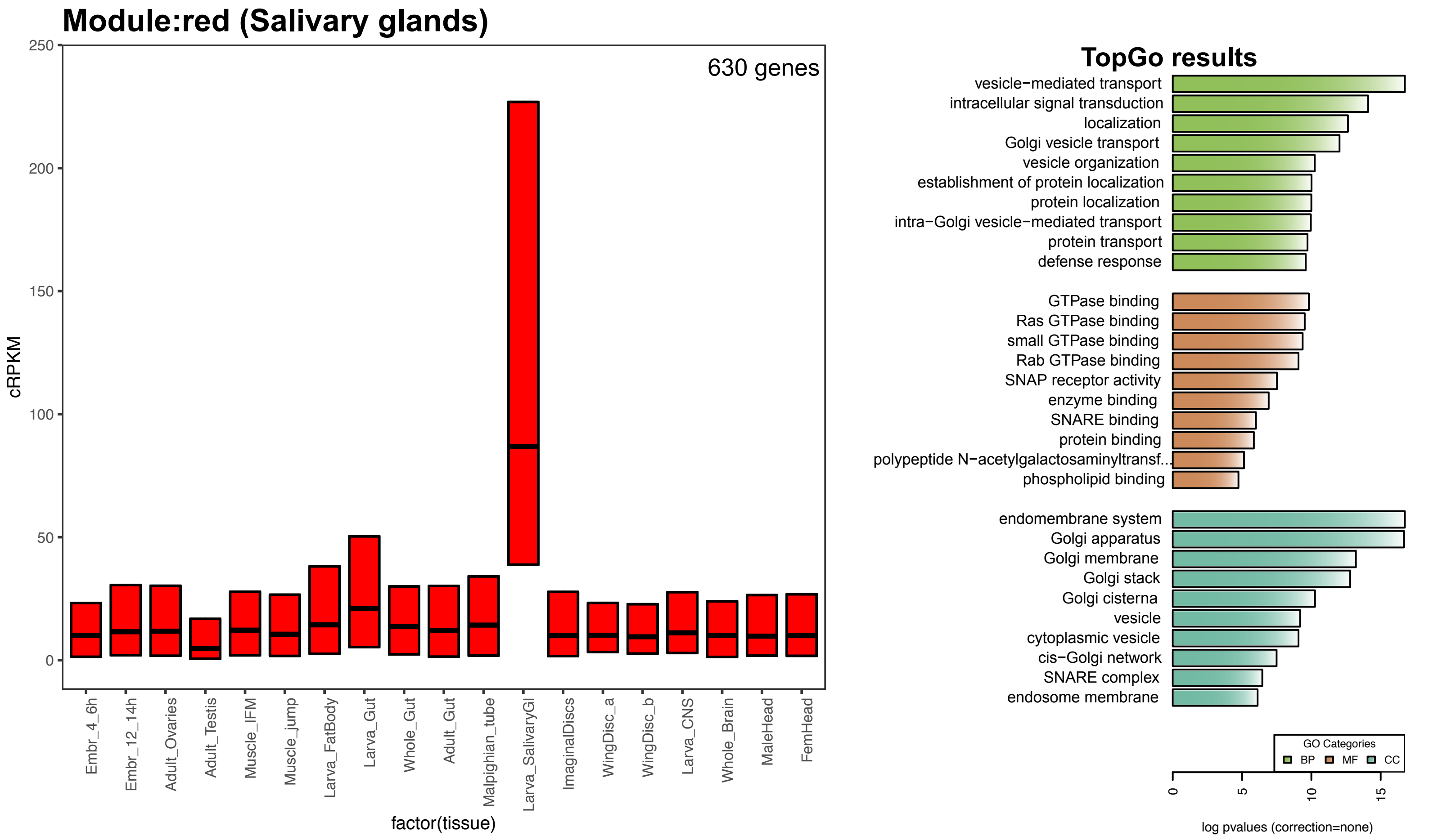

D. melanogaster modules

Module:blue (Brain - Synapsis)

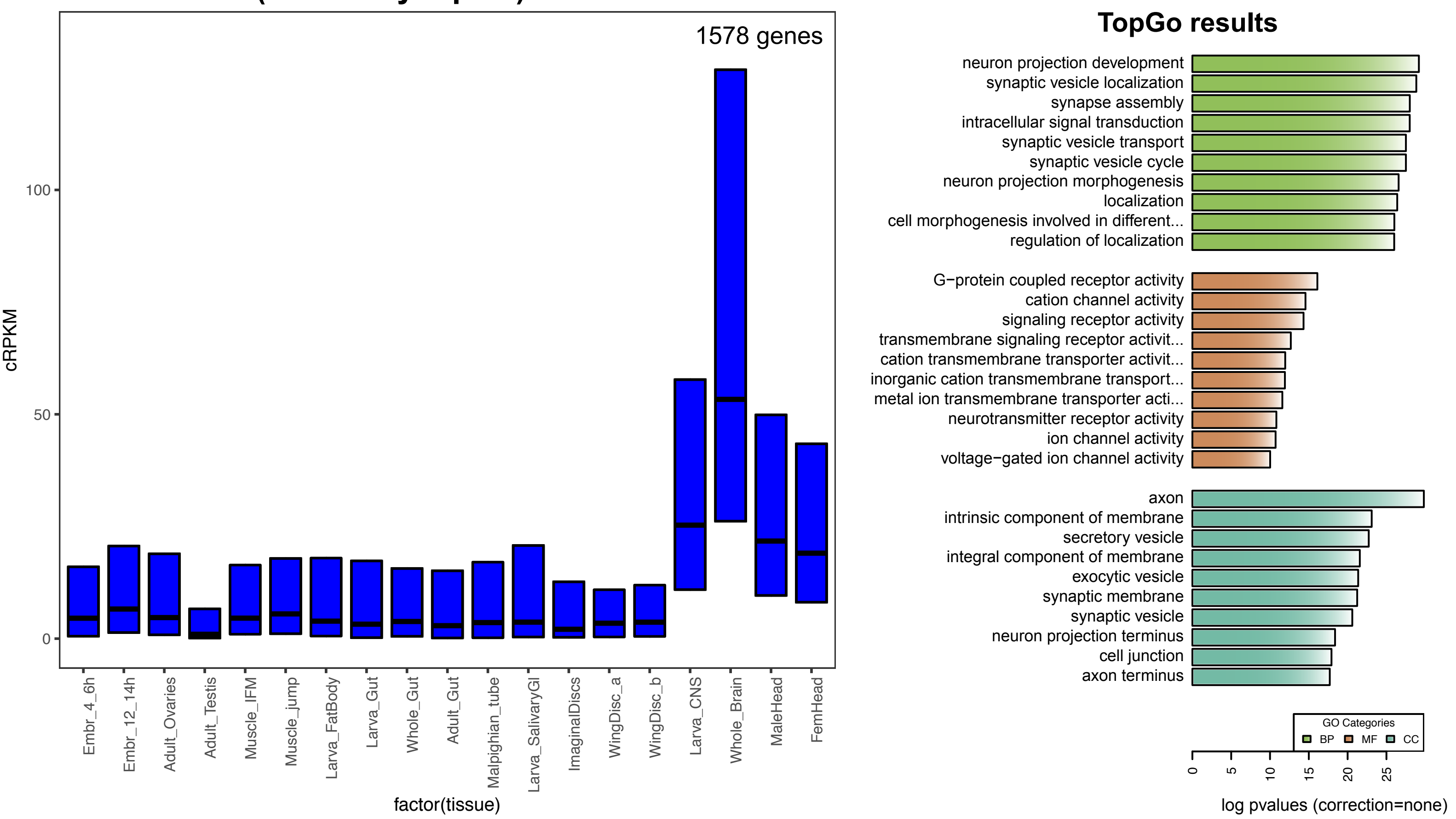

Module:greenyellow (Wing disc)

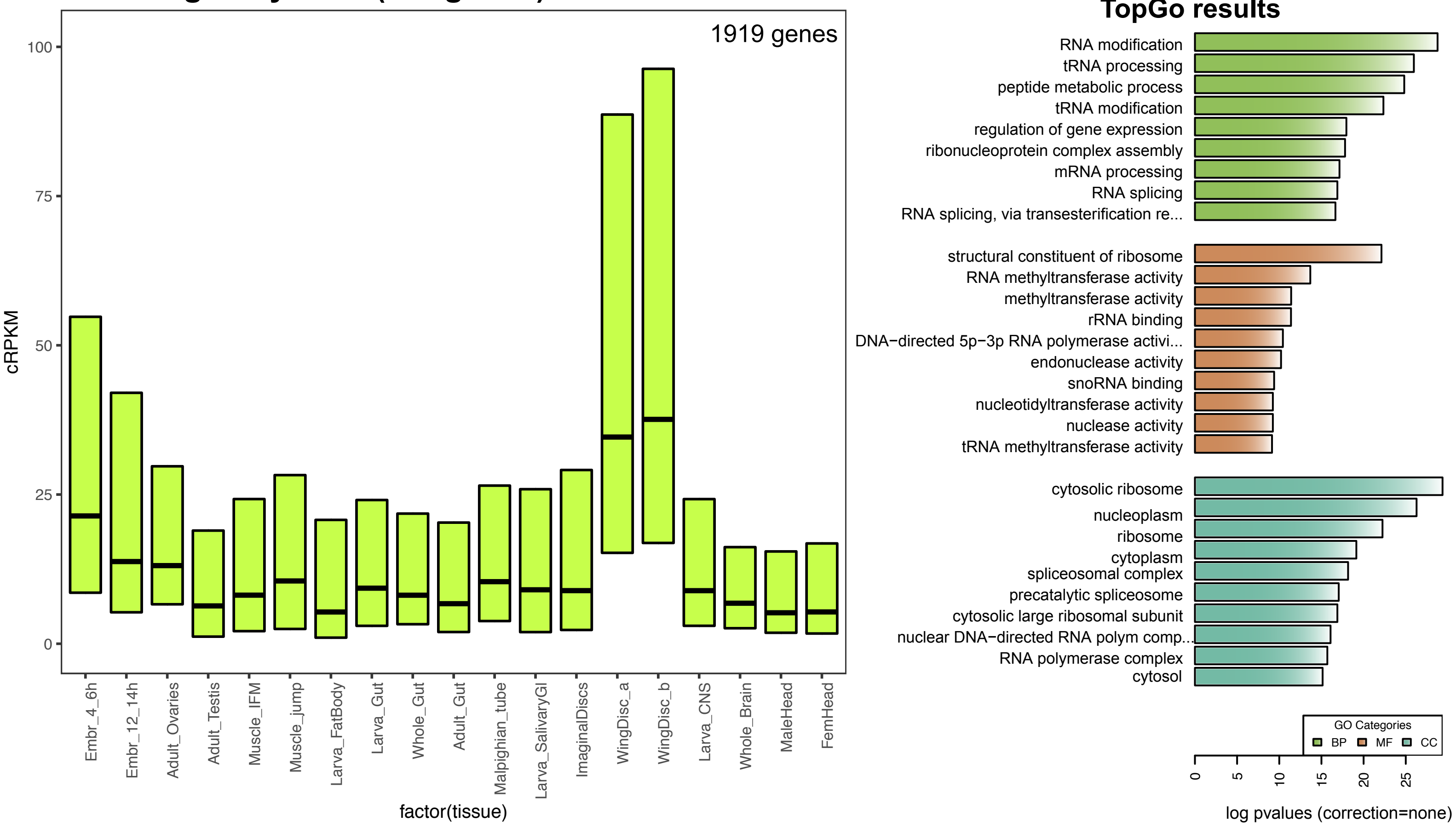

D. melanogaster modules

Module:cyan (Embryo 12-14 hours)

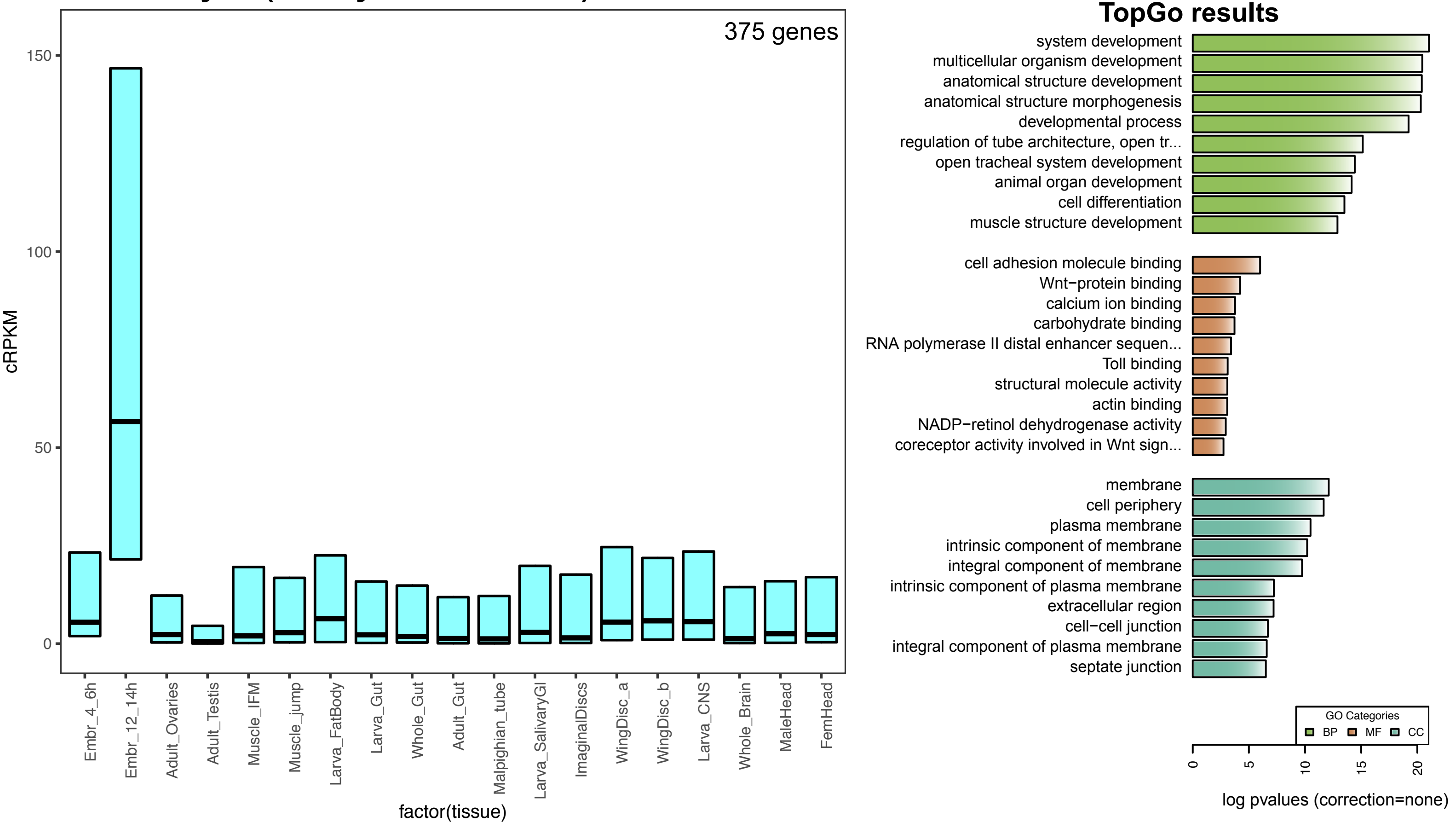

Module:black (Flight Muscle)

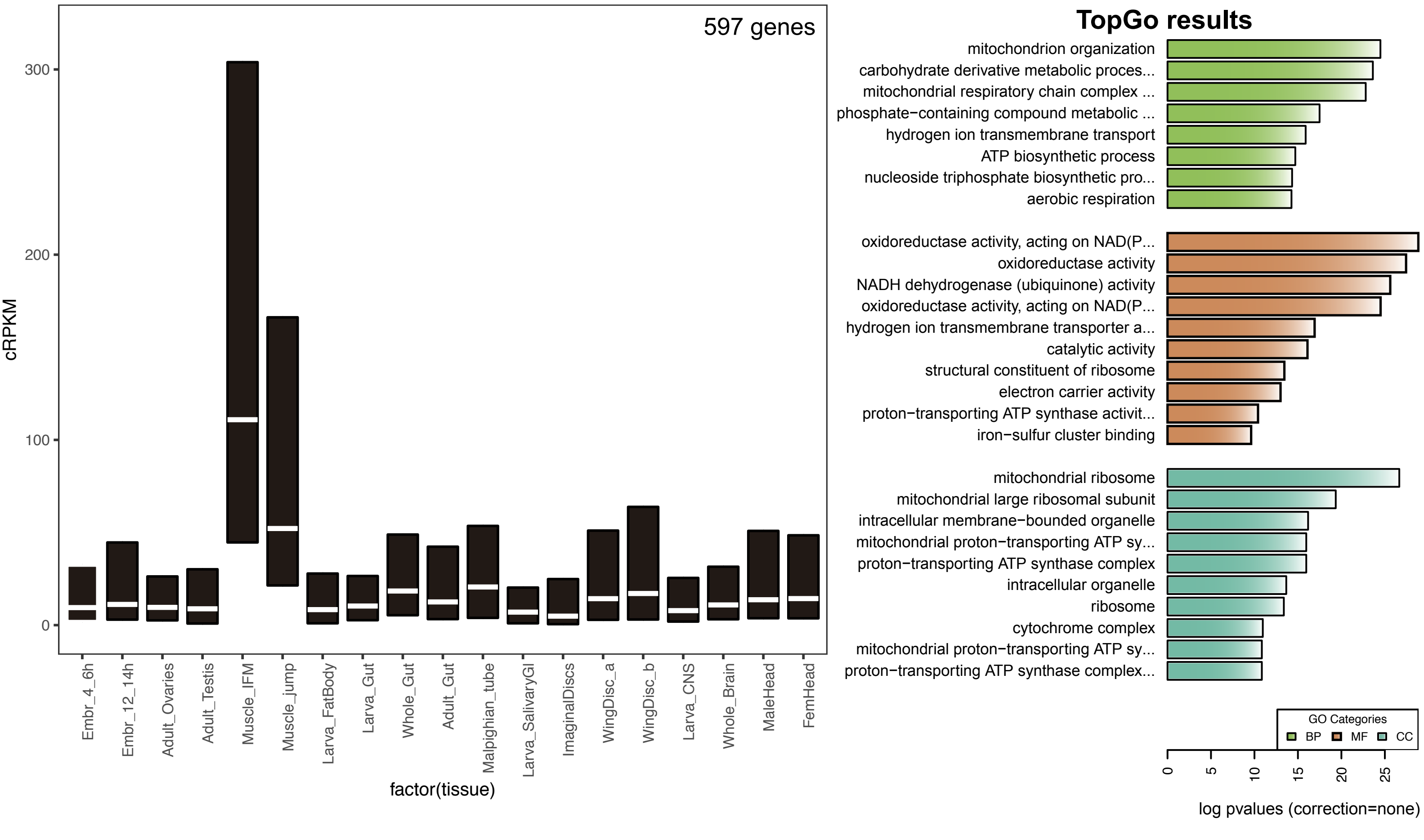

D. melanogaster modules

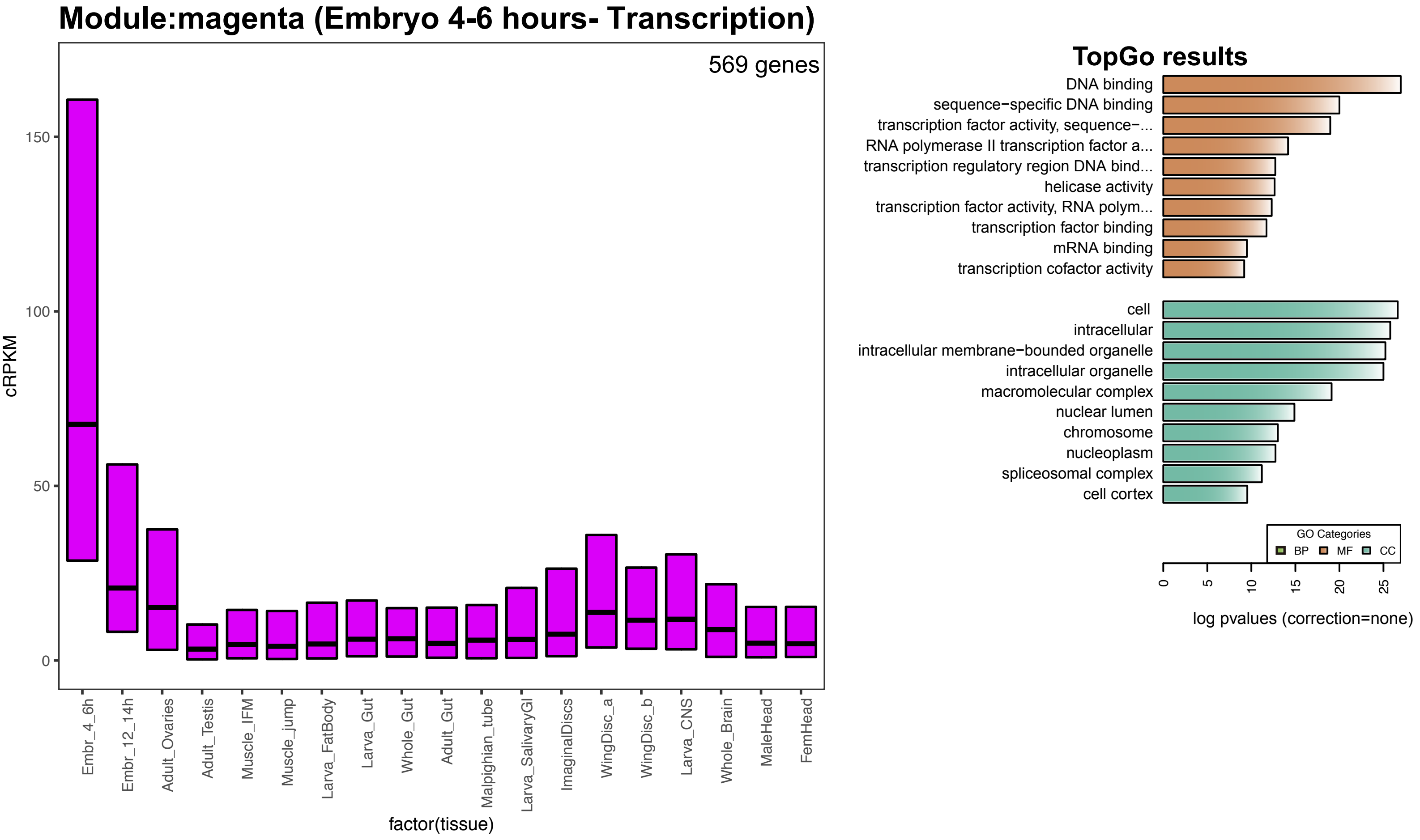

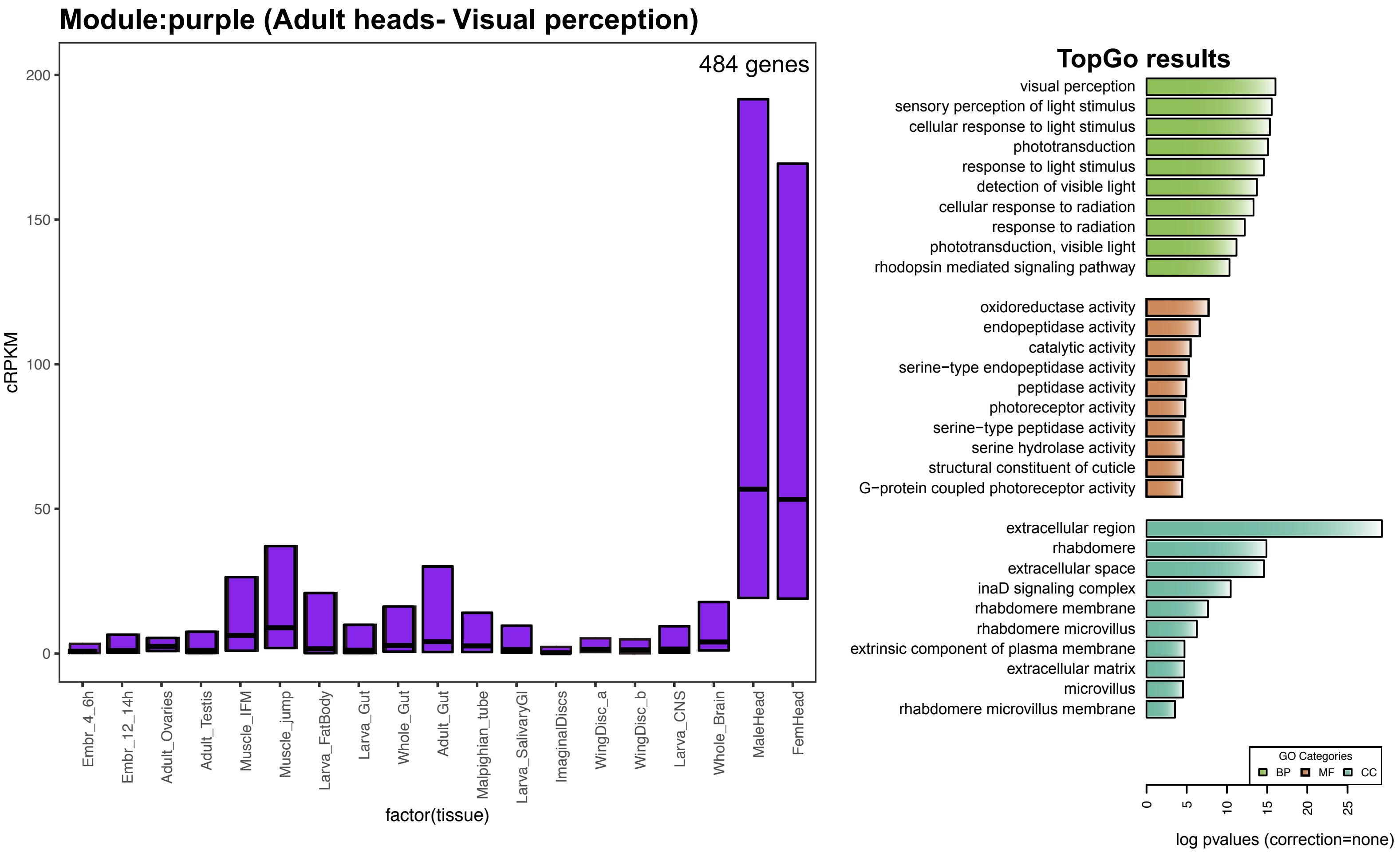

D. melanogaster modules

Module:white (Muscle-Cellular metabolism)

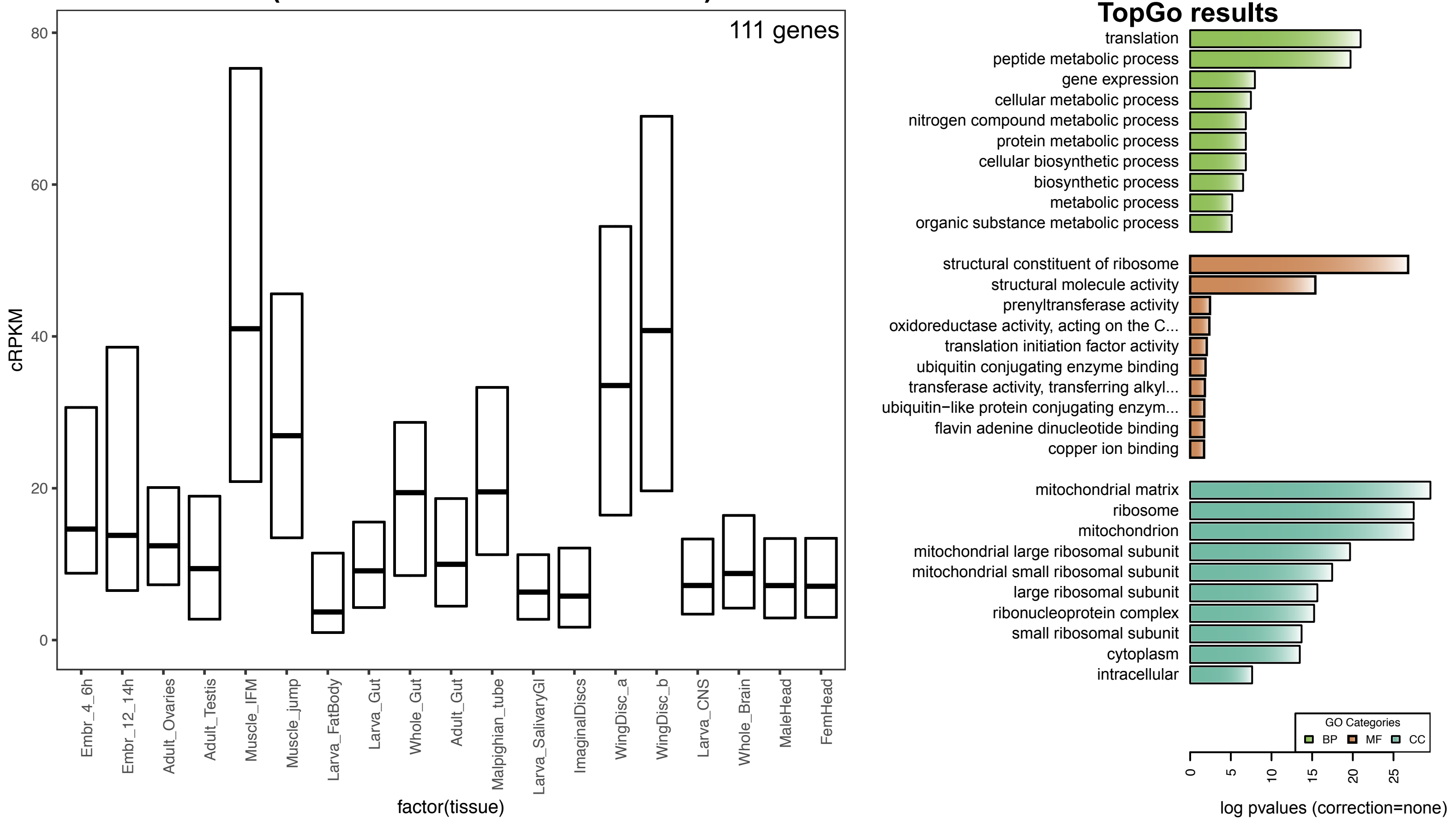

Module:brown (Testes)

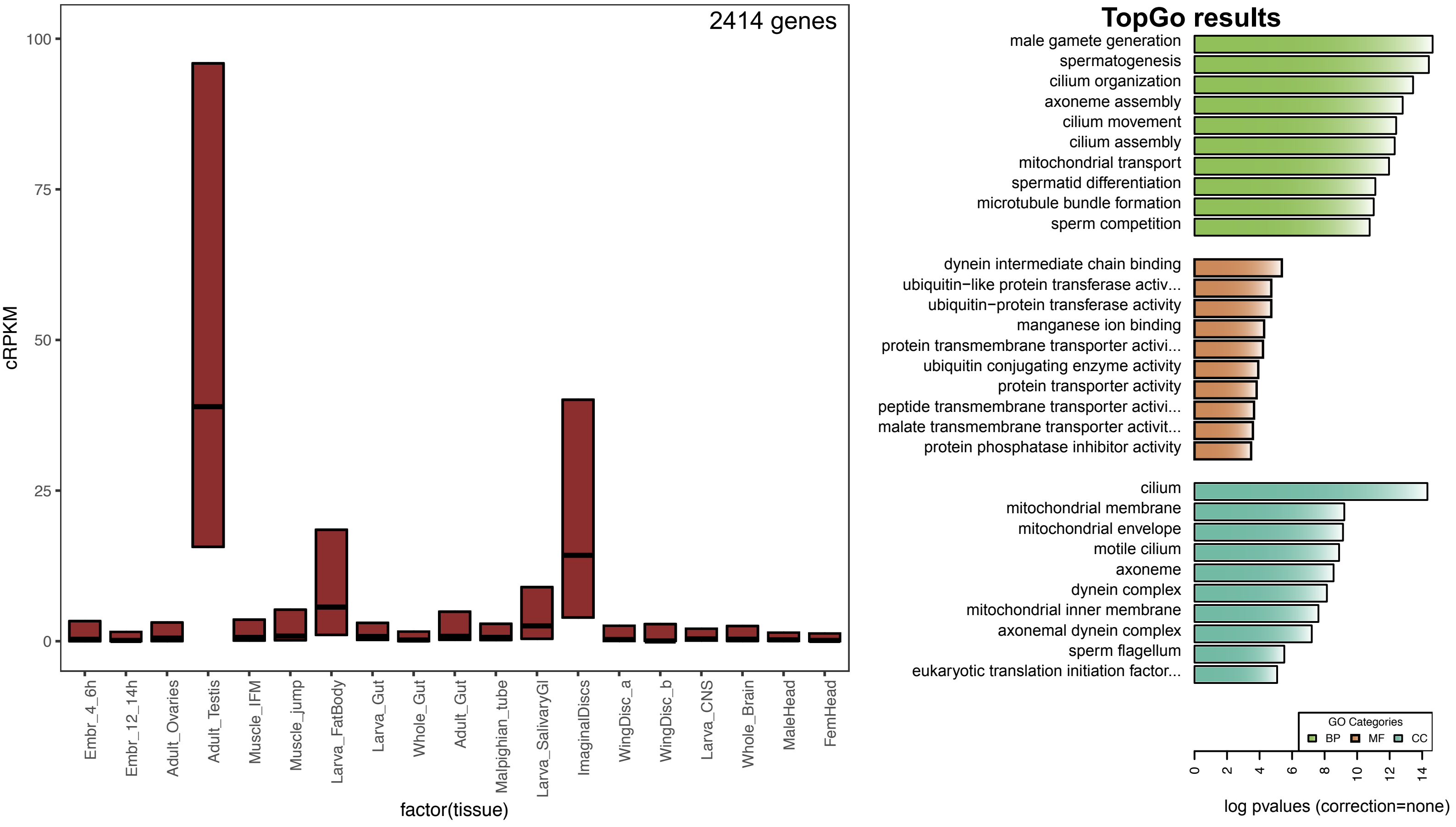

D. melanogaster modules

Module:pink (Malpighian tubules)

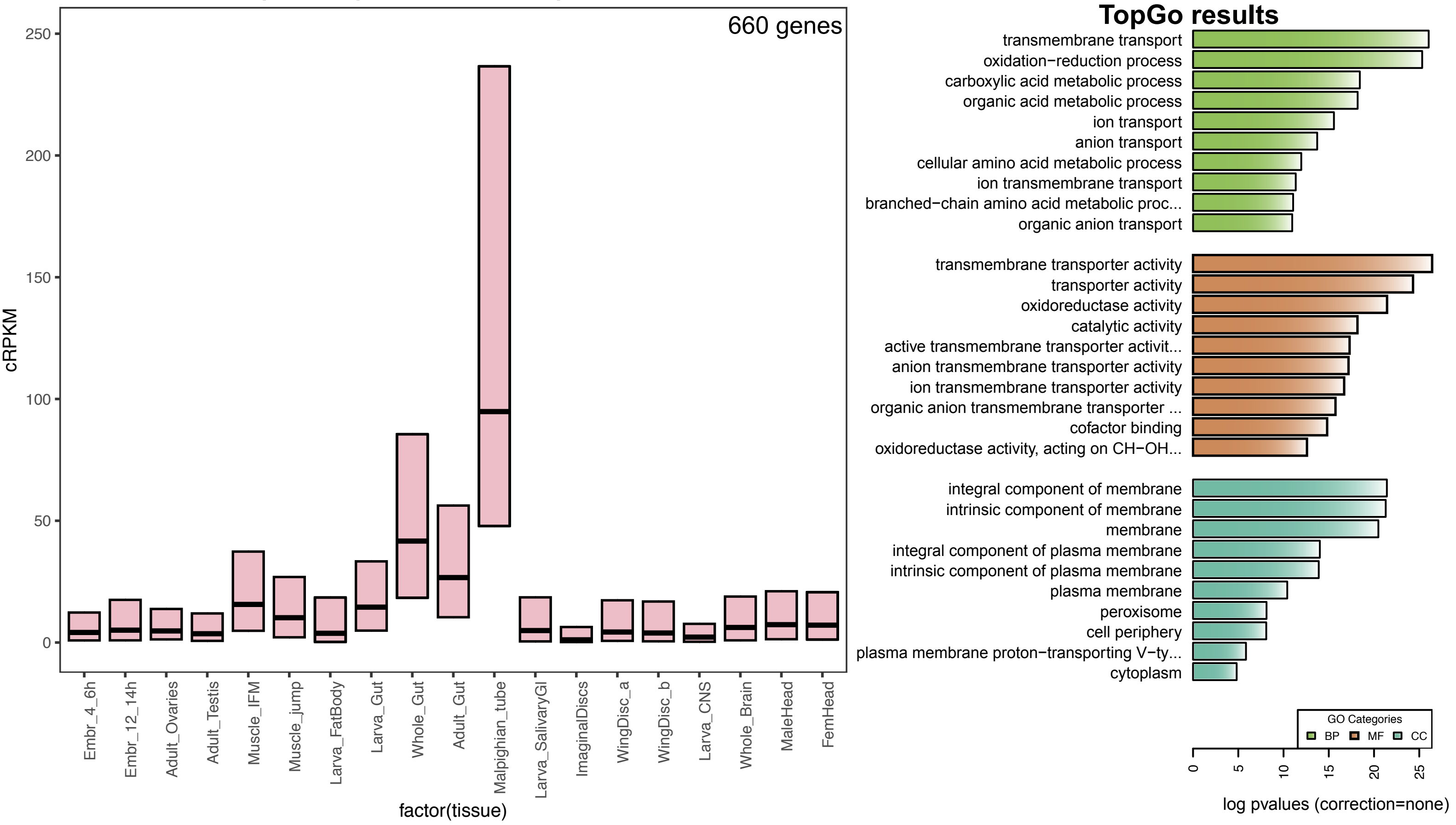

Module:skyblue (Adult gut)

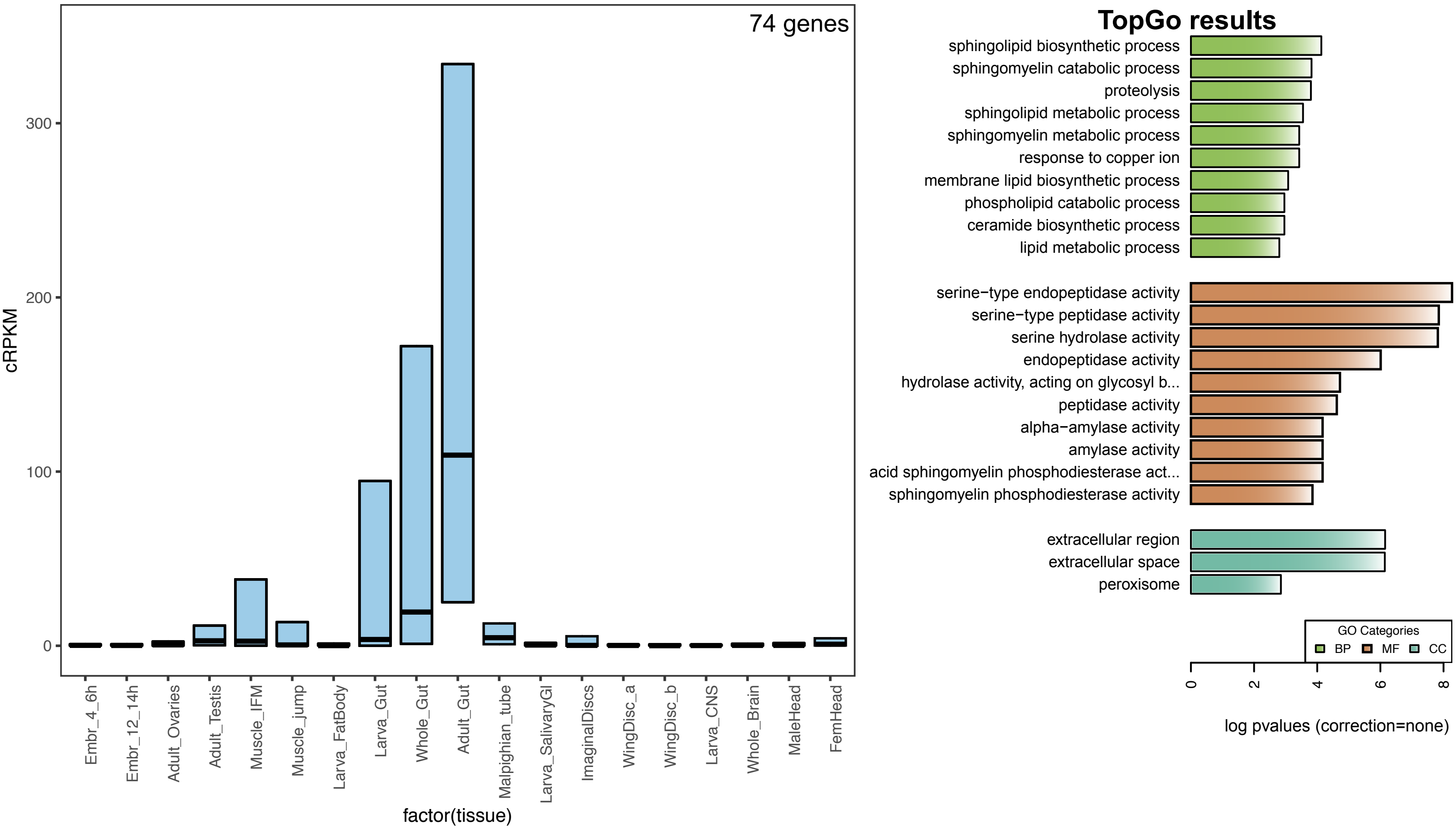

D. melanogaster modules

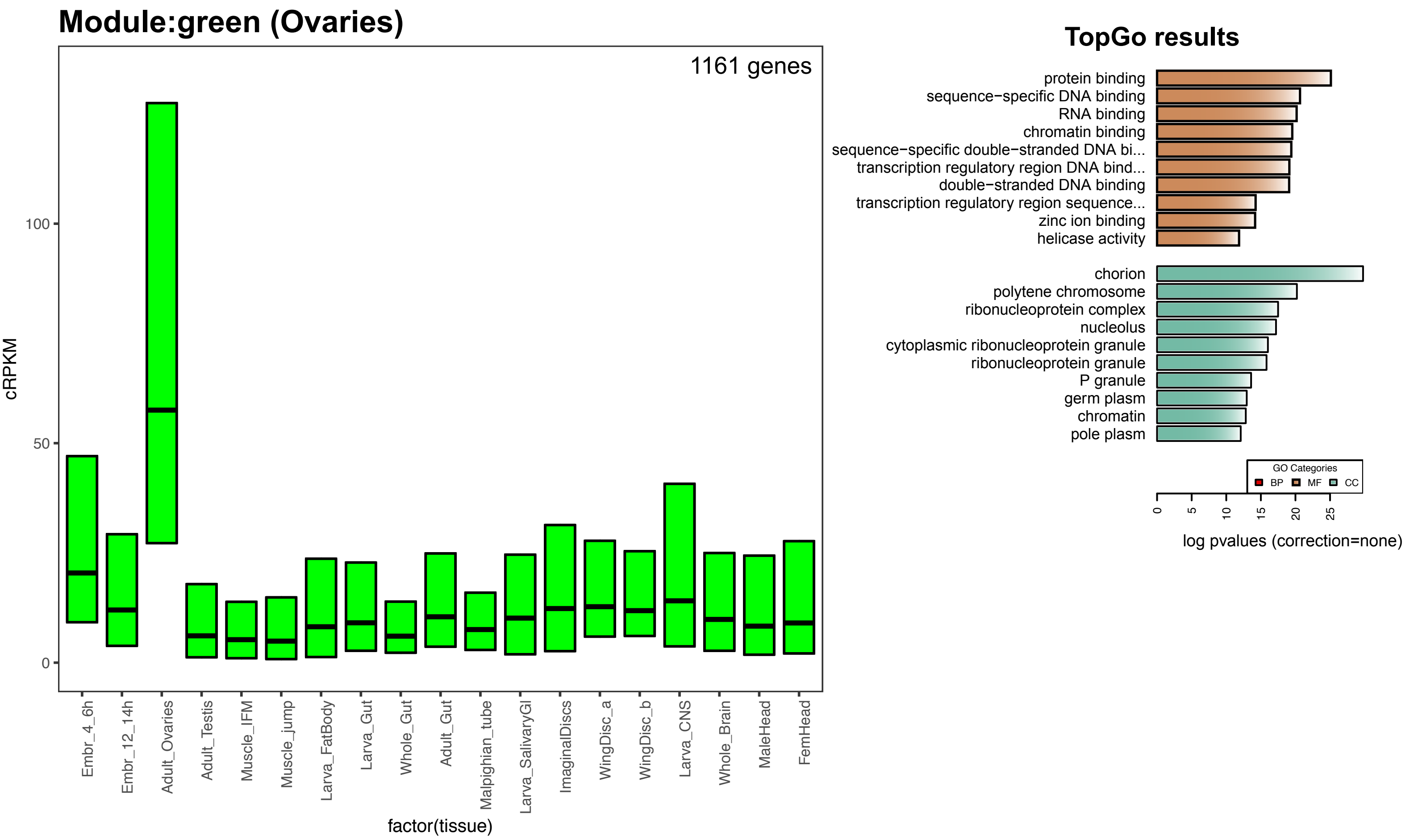

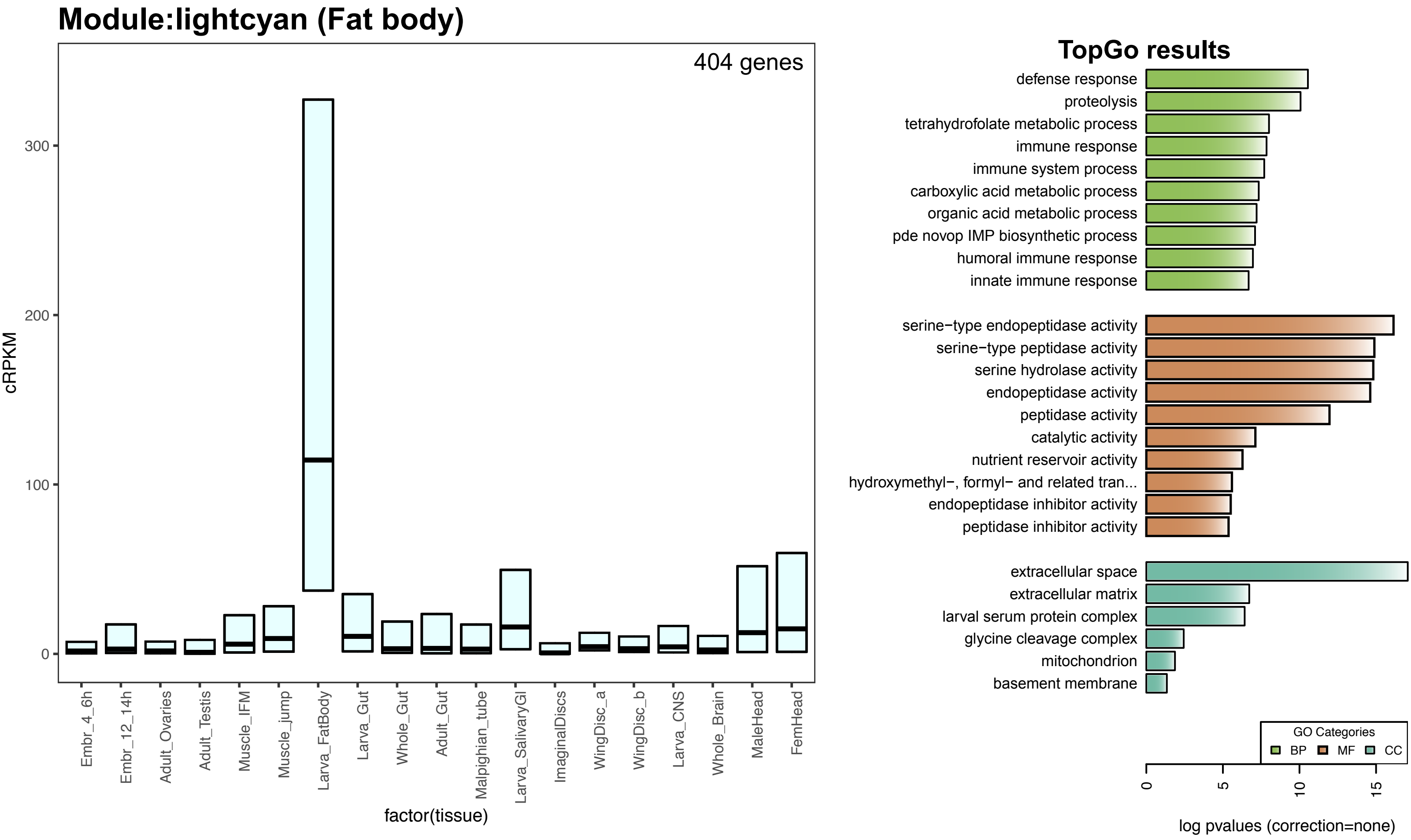

D. melanogaster modules

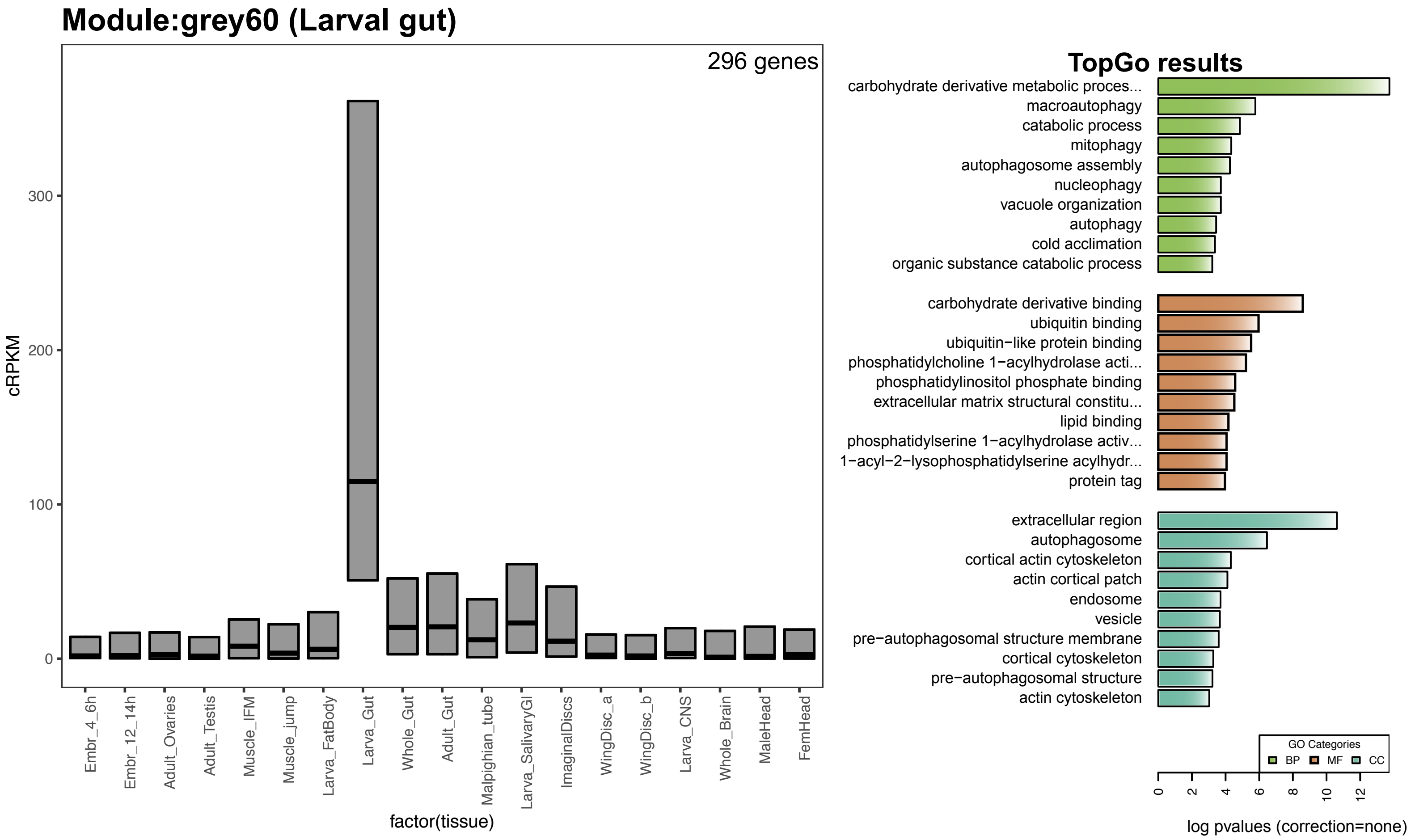

S. maritima modules

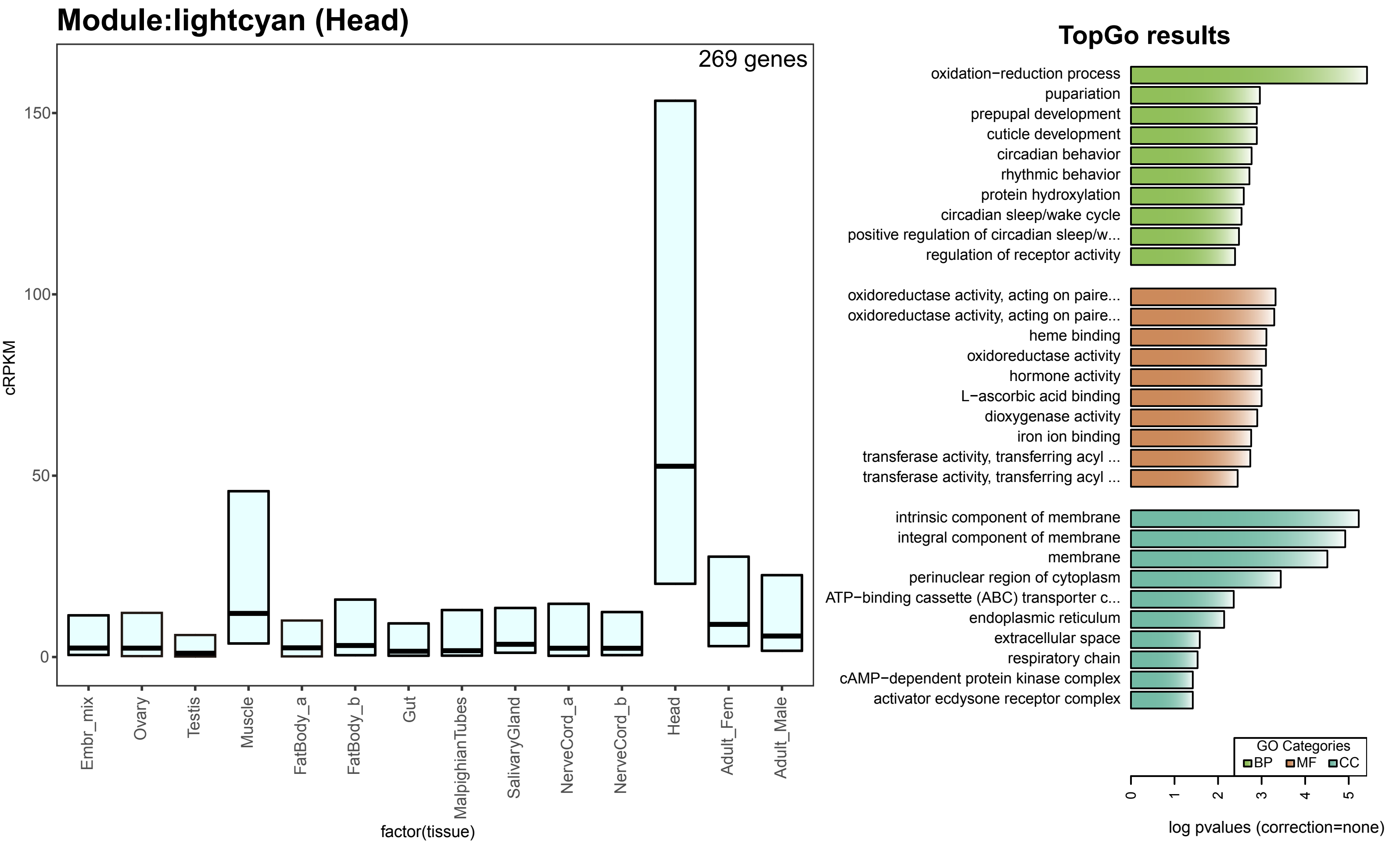

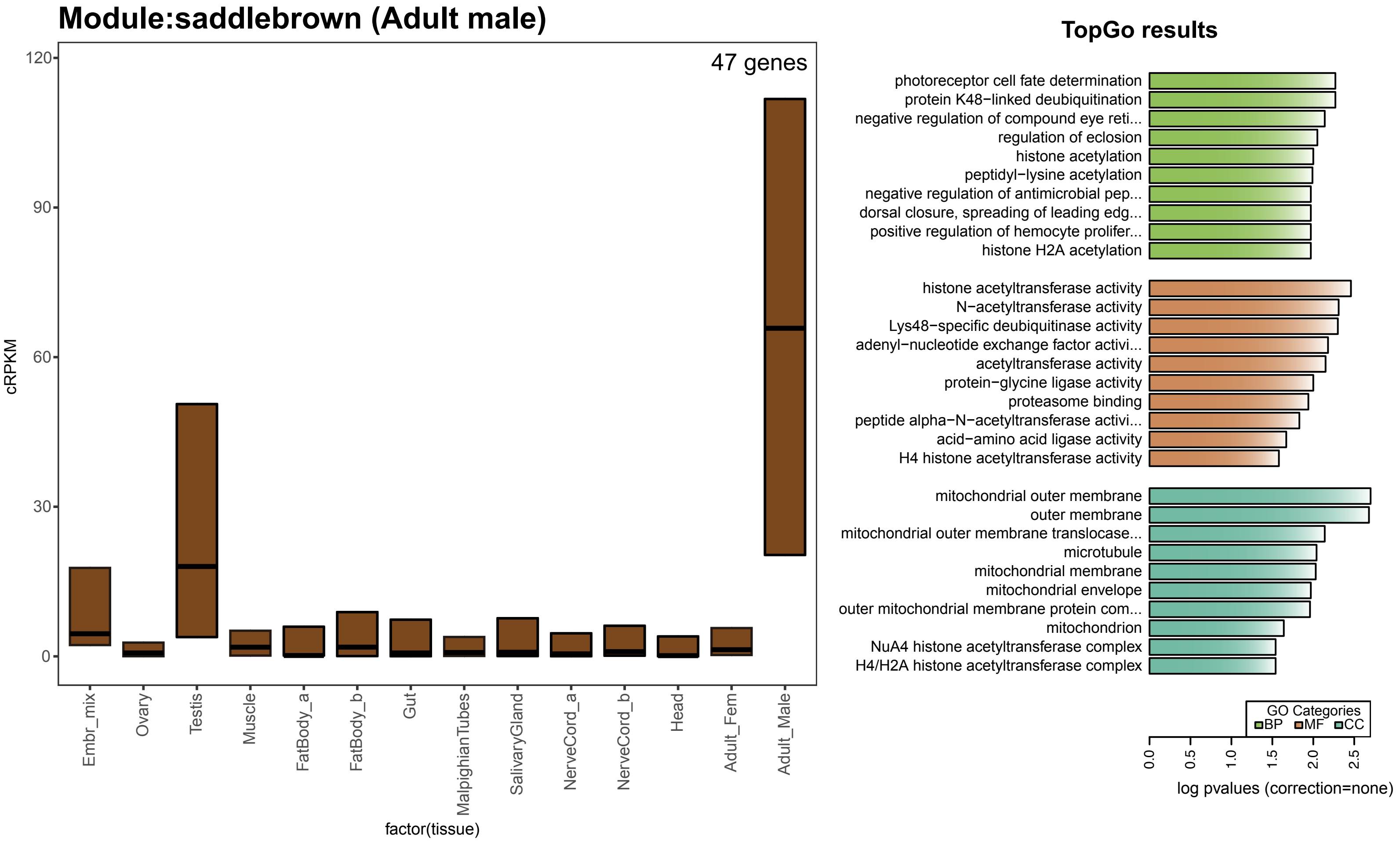

S. maritima modules

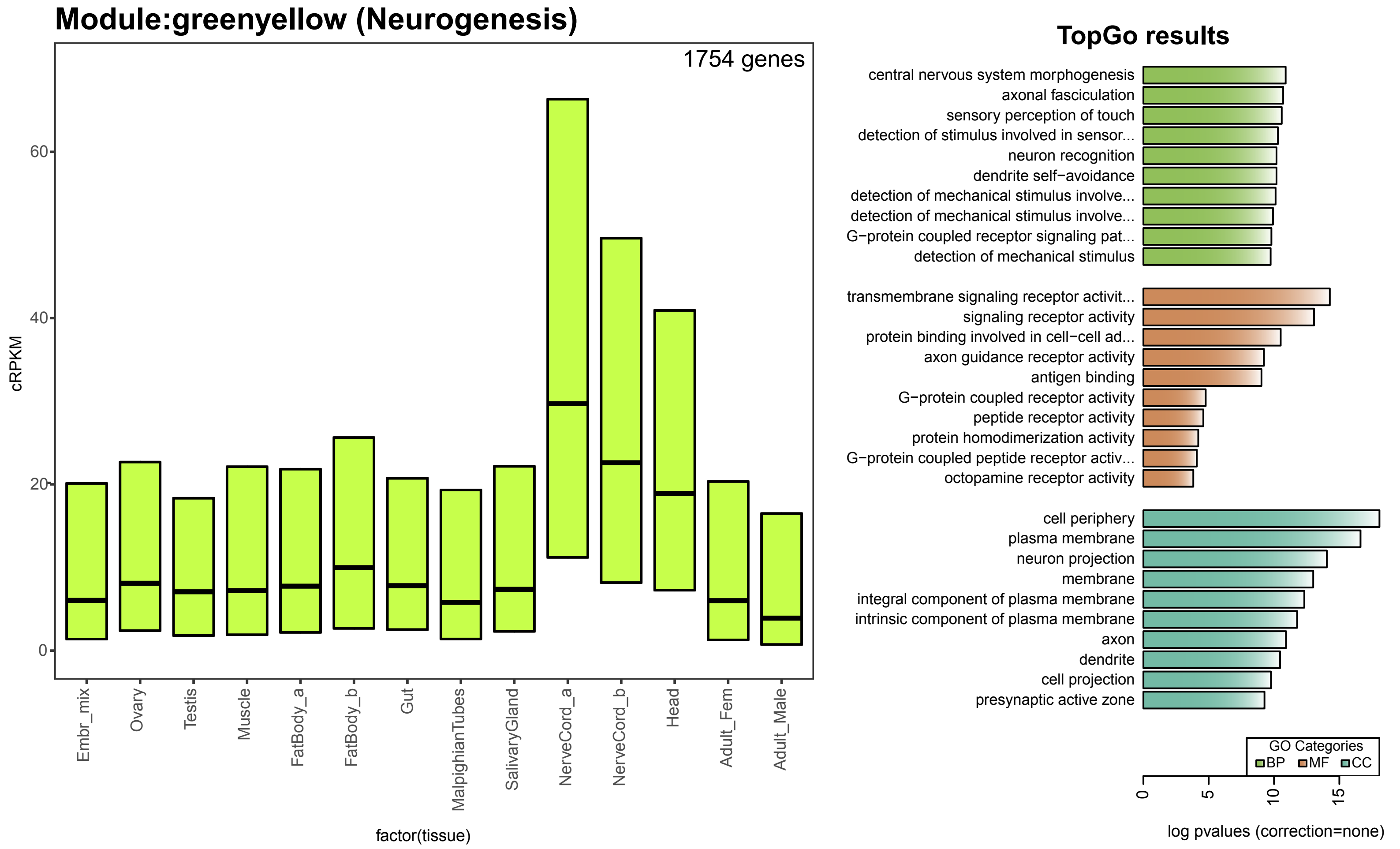

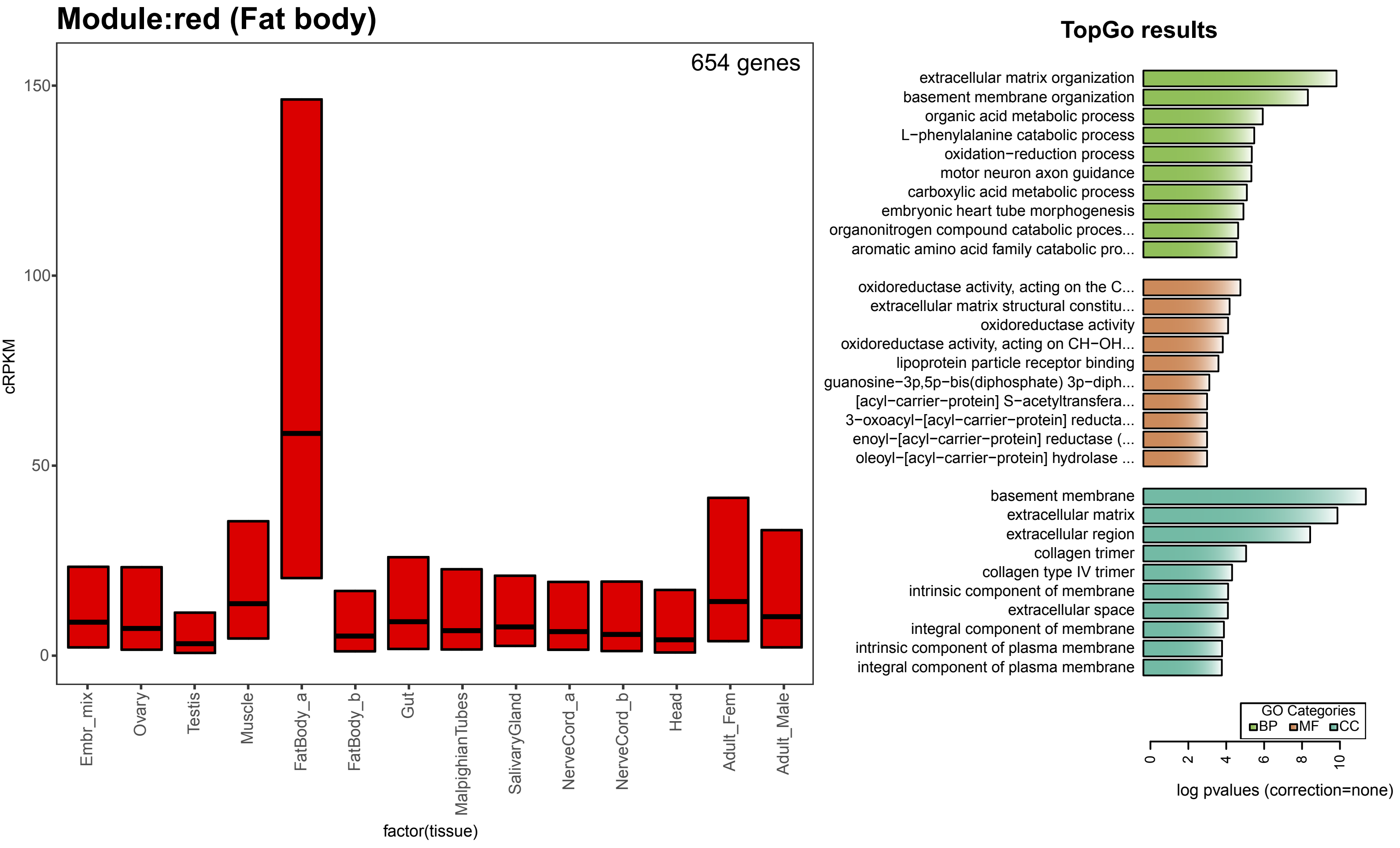

S. maritima modules

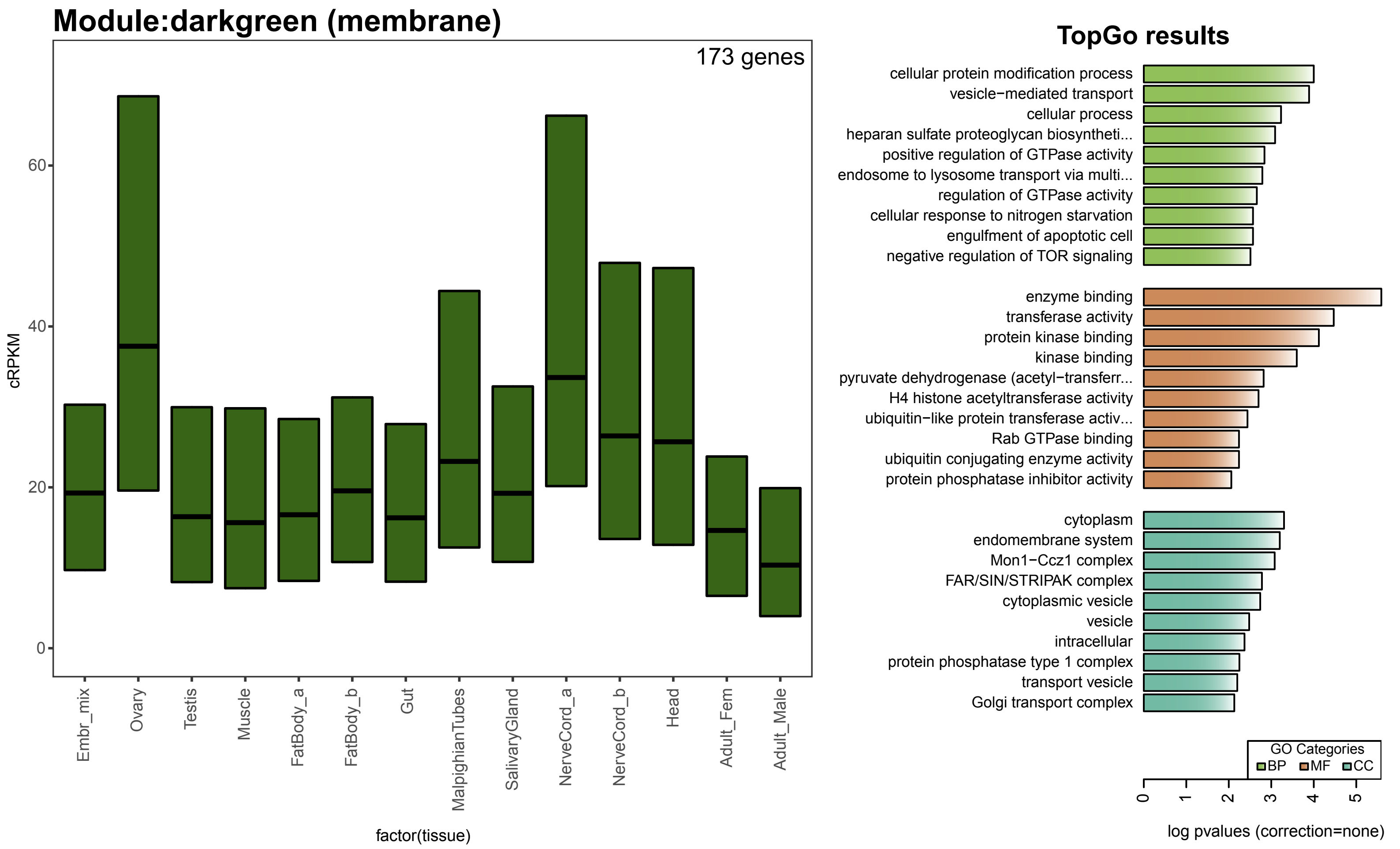

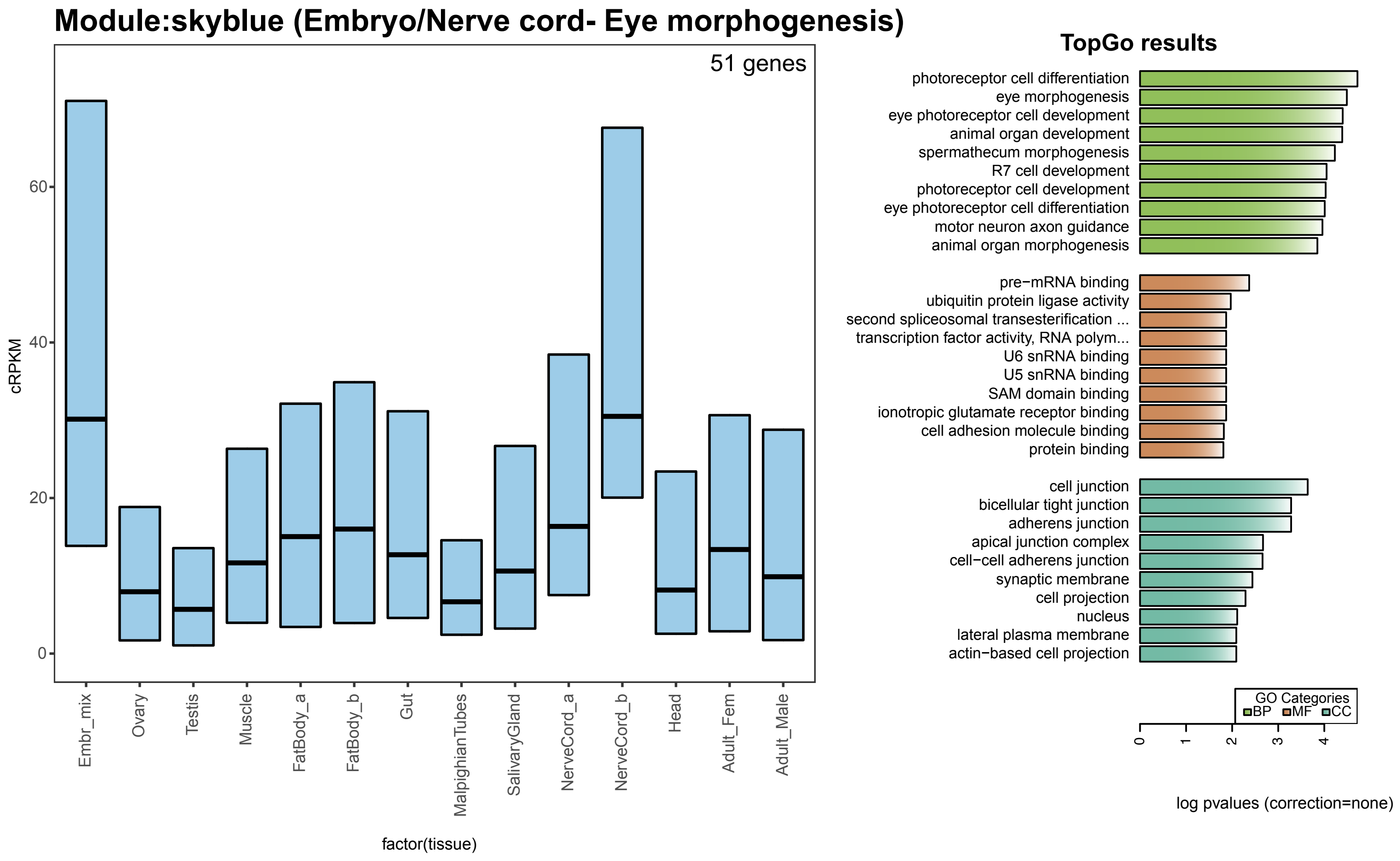

S. maritima modules

Module:orange (Protein synthesis)

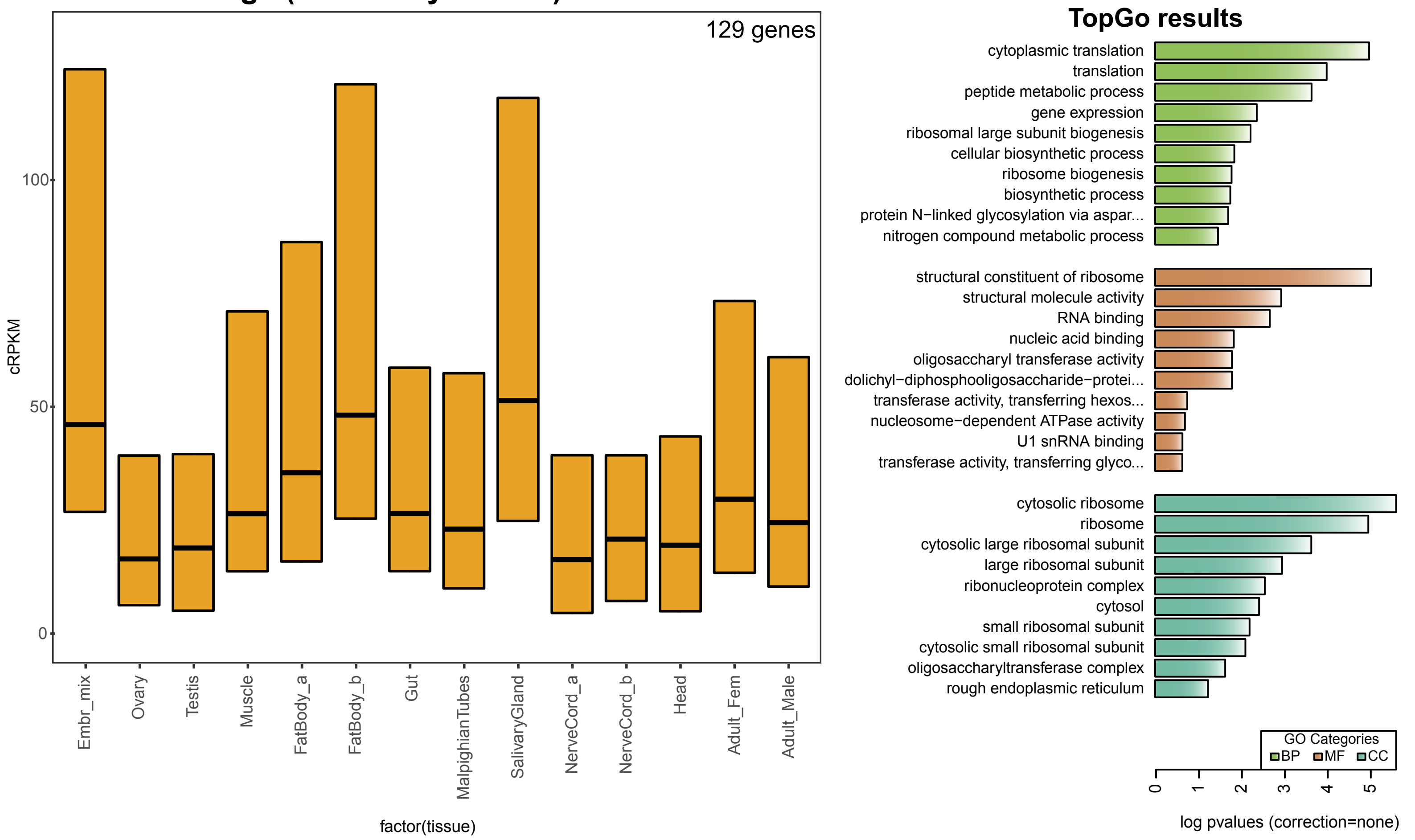

Module:black (Nerve Cord- Synapsis)

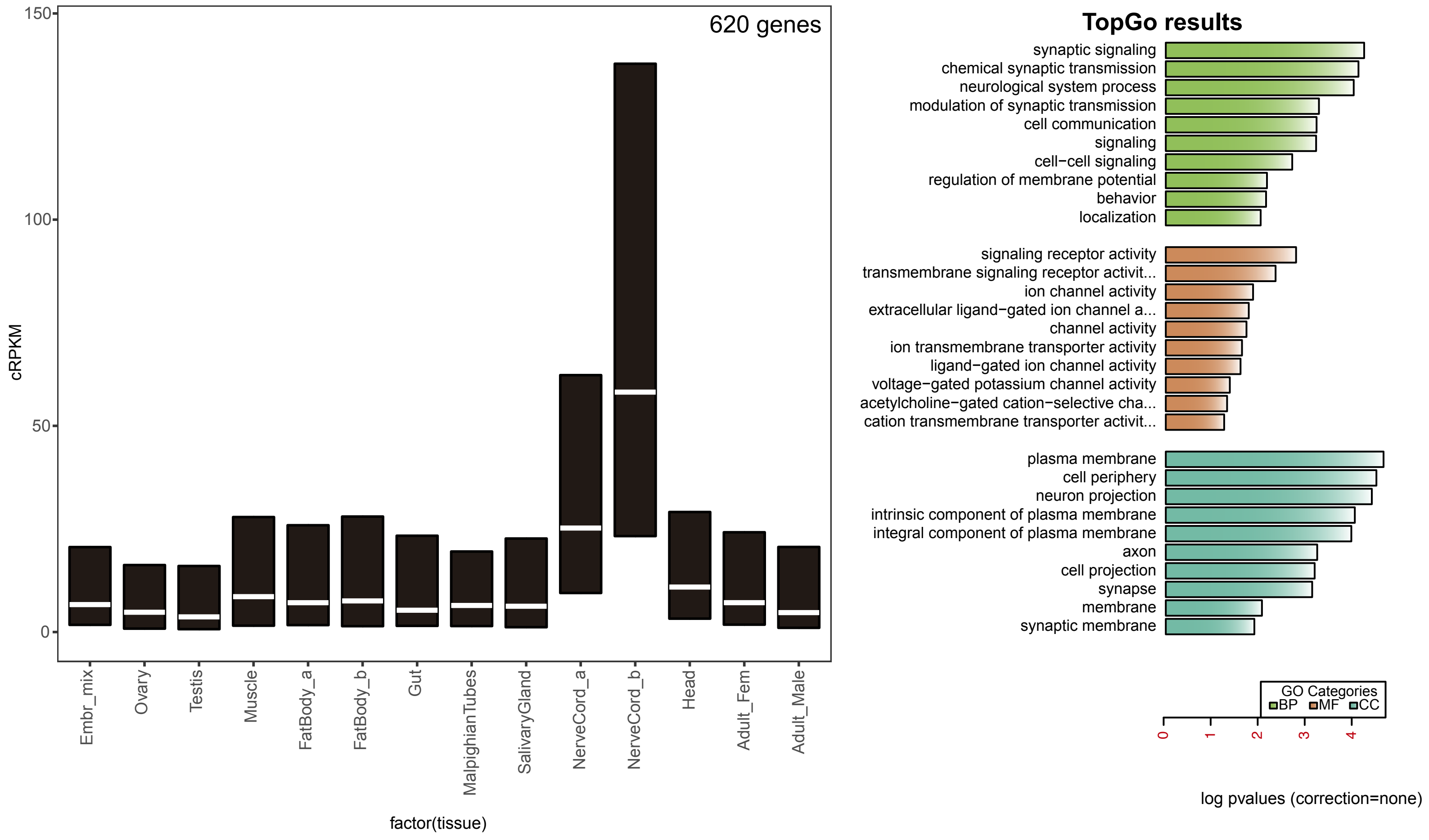

S. maritima modules

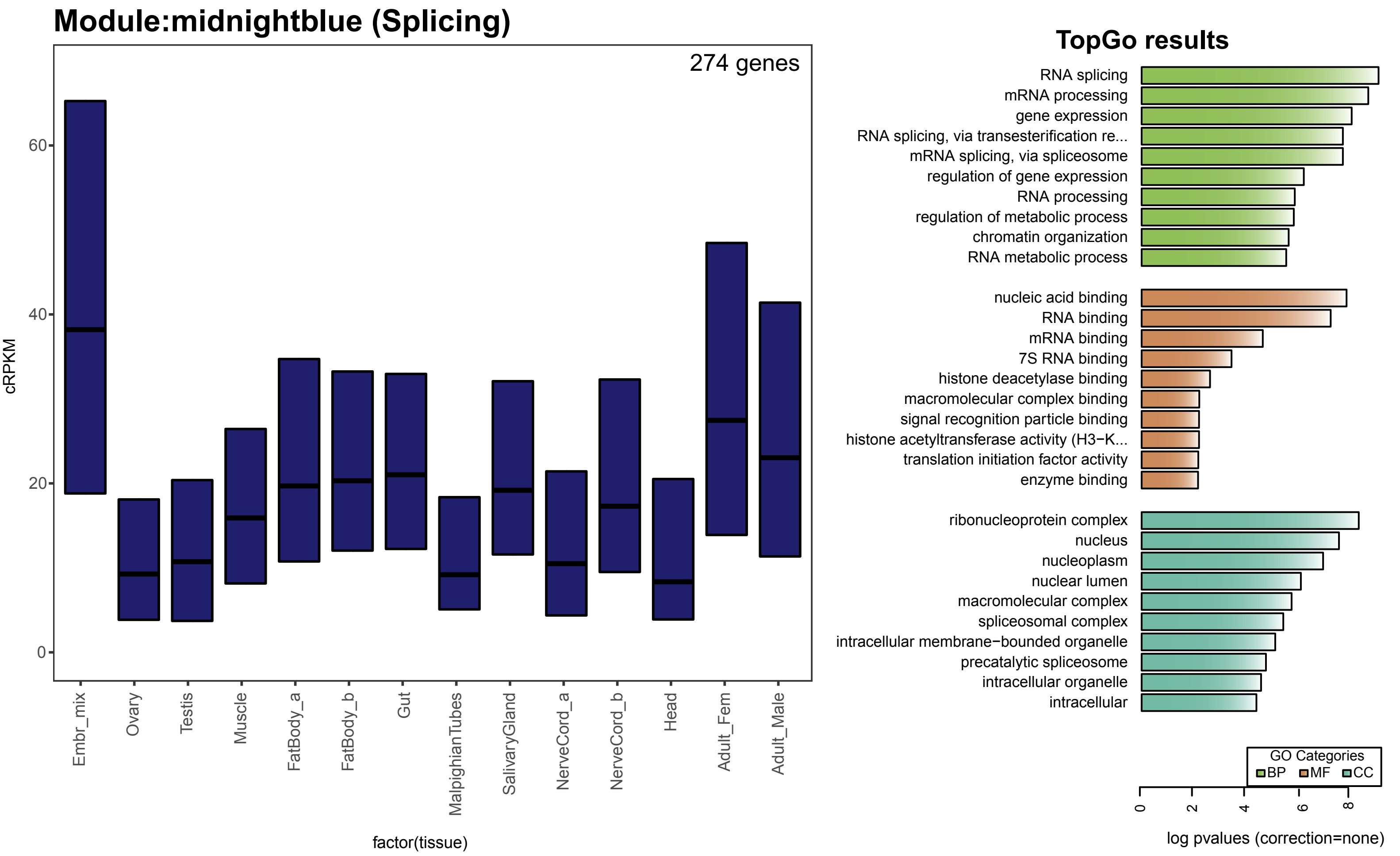

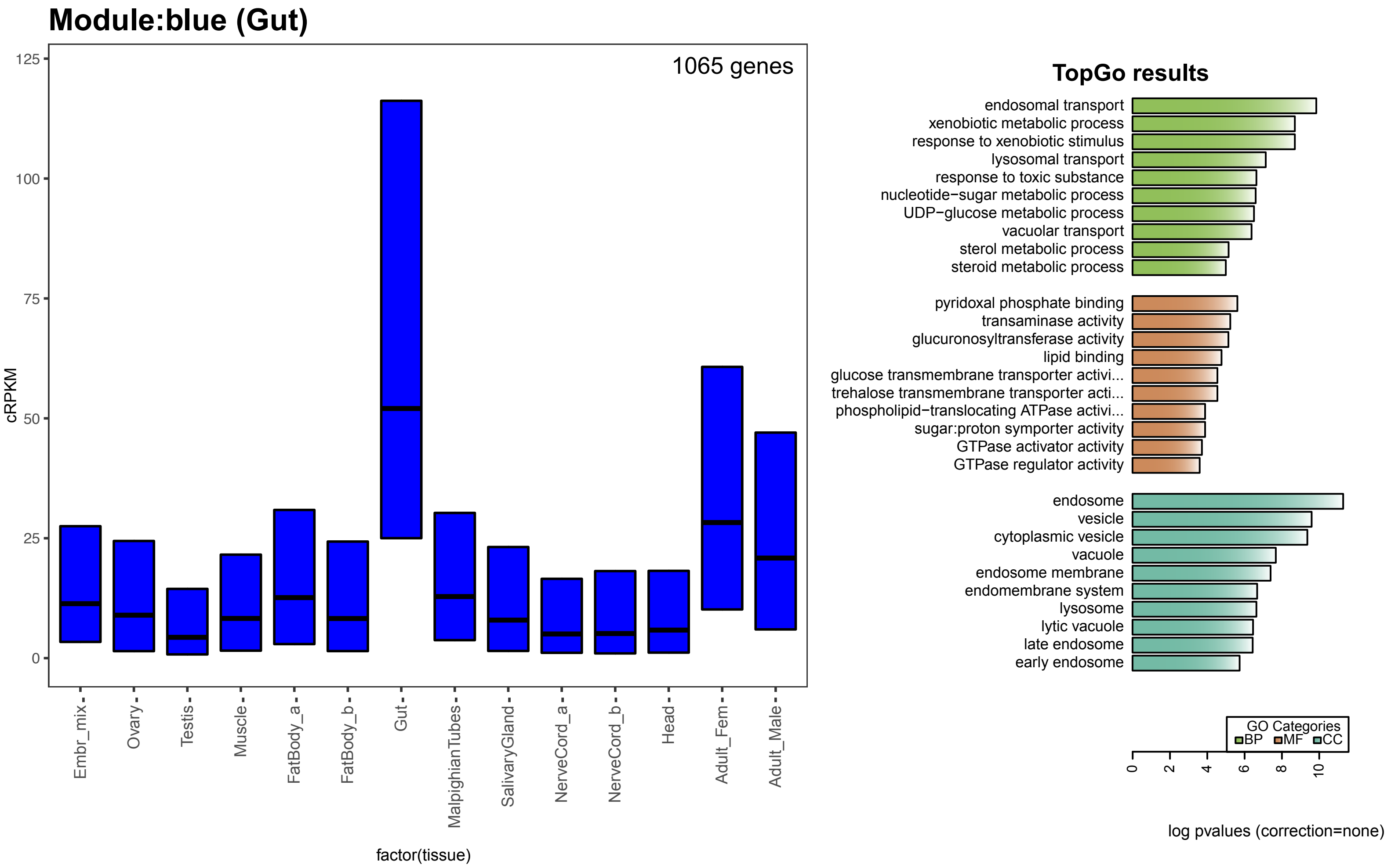

S. maritima modules

Module:grey60 (Malpighian tubules)

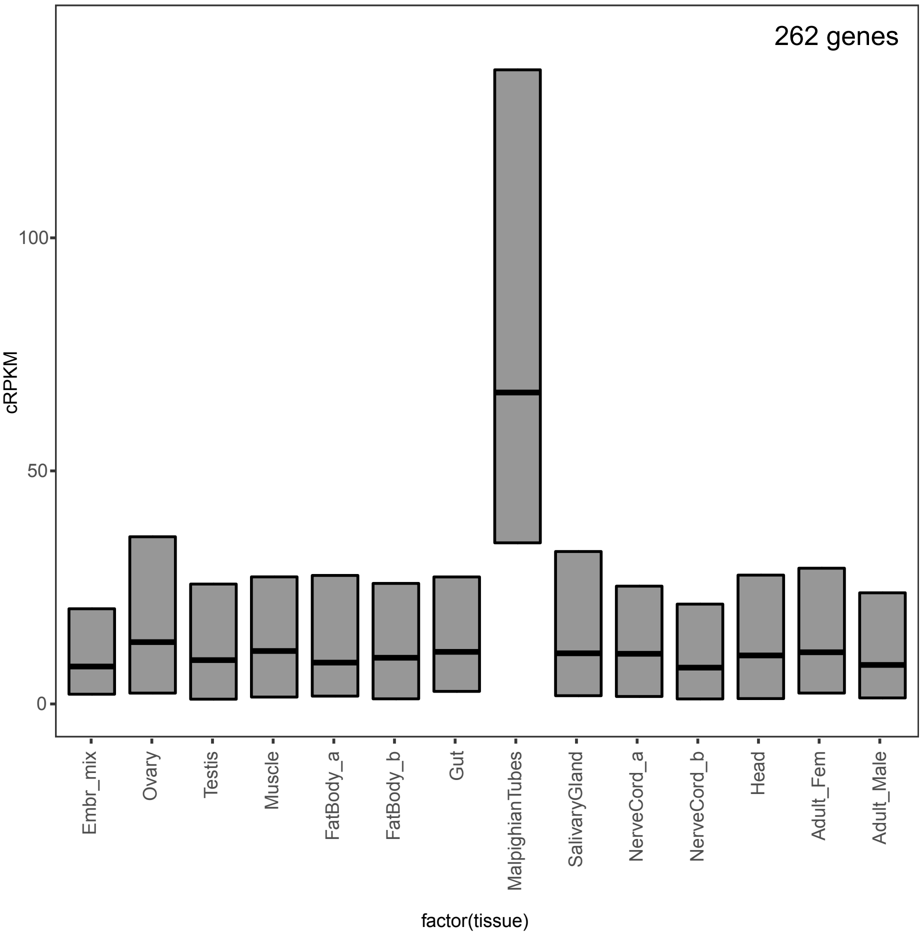

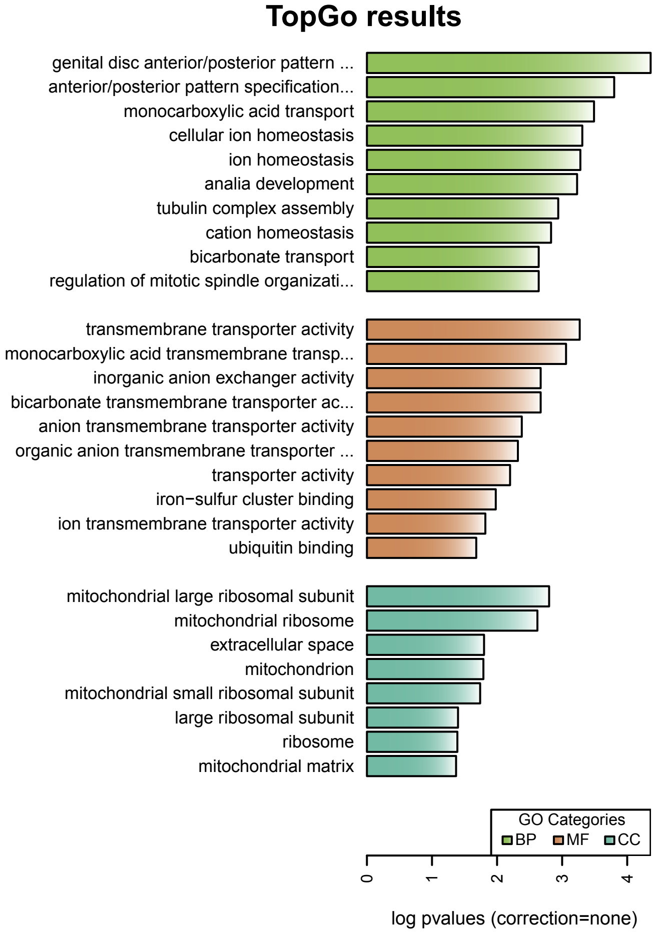

Module:purple (Fat body/Salivary gland- Translation)

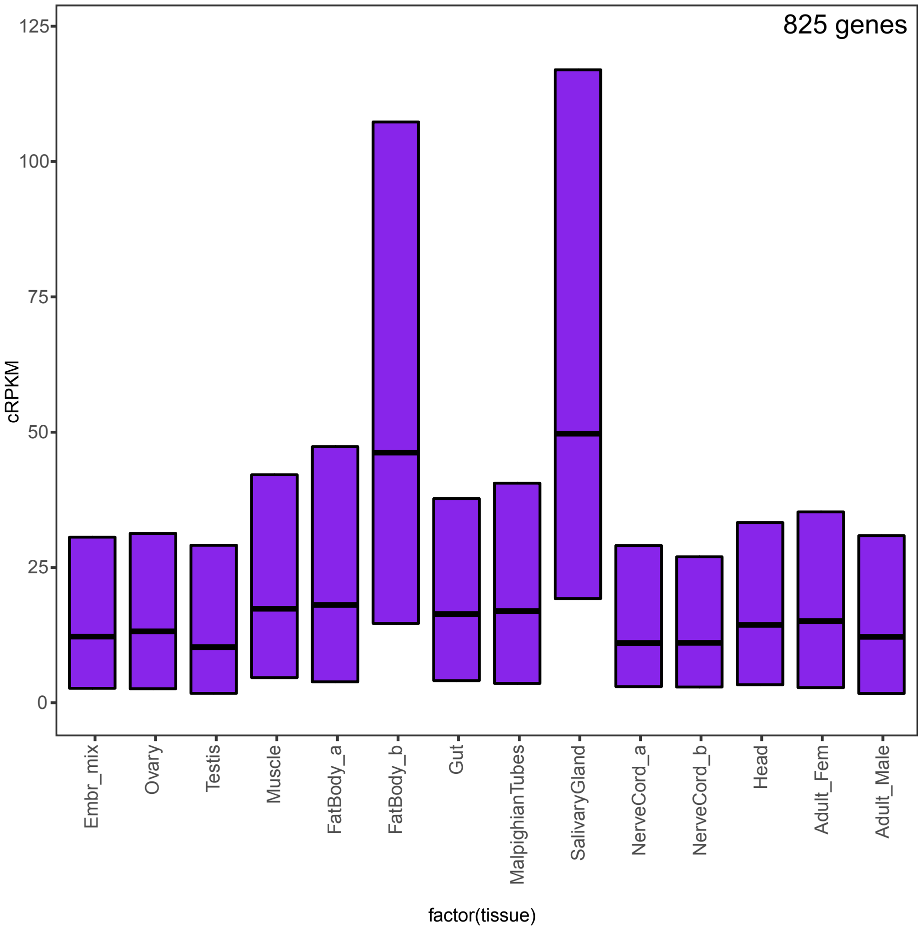

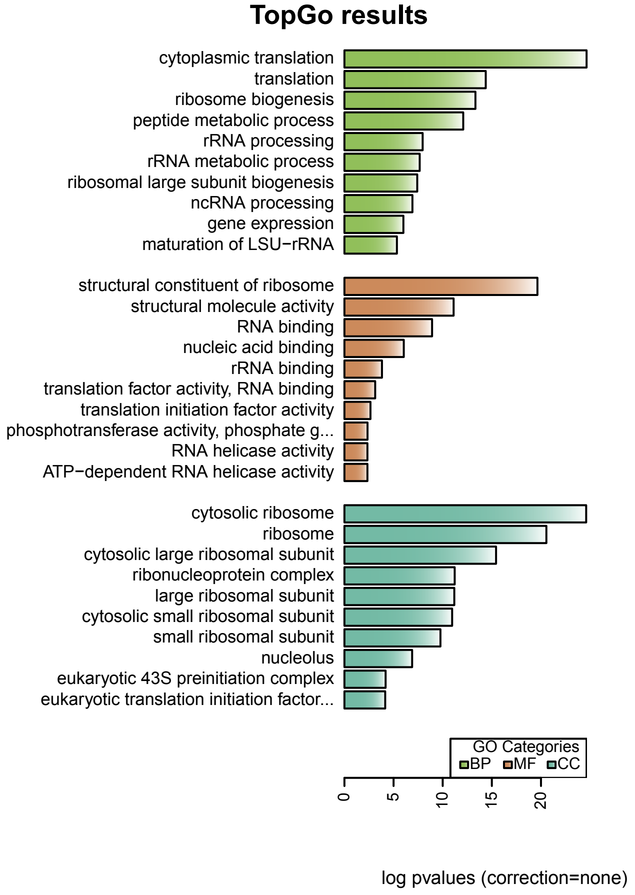

S. maritima modules

Module:white (Proteasome)

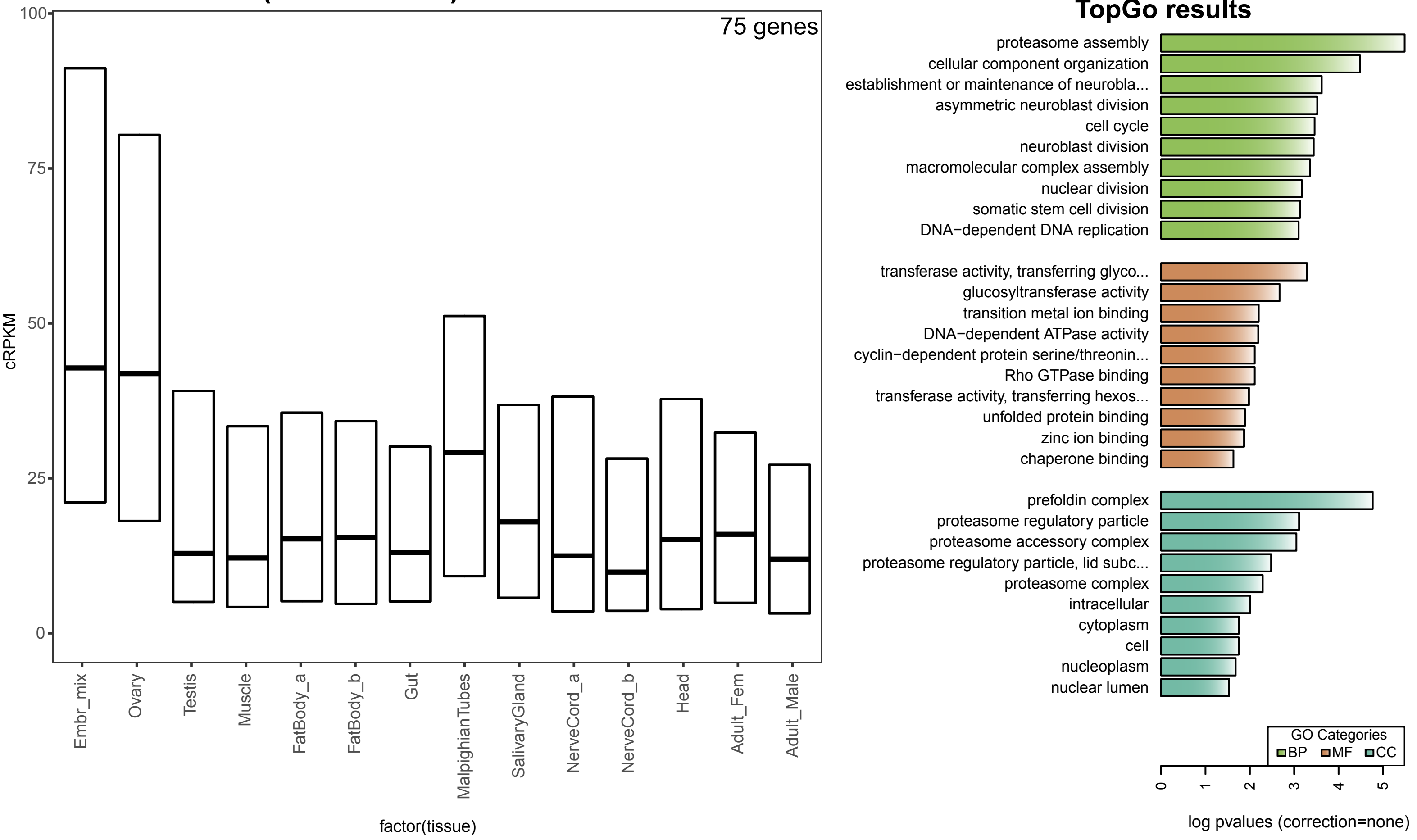

Module:lightgreen (Cellular respiration)

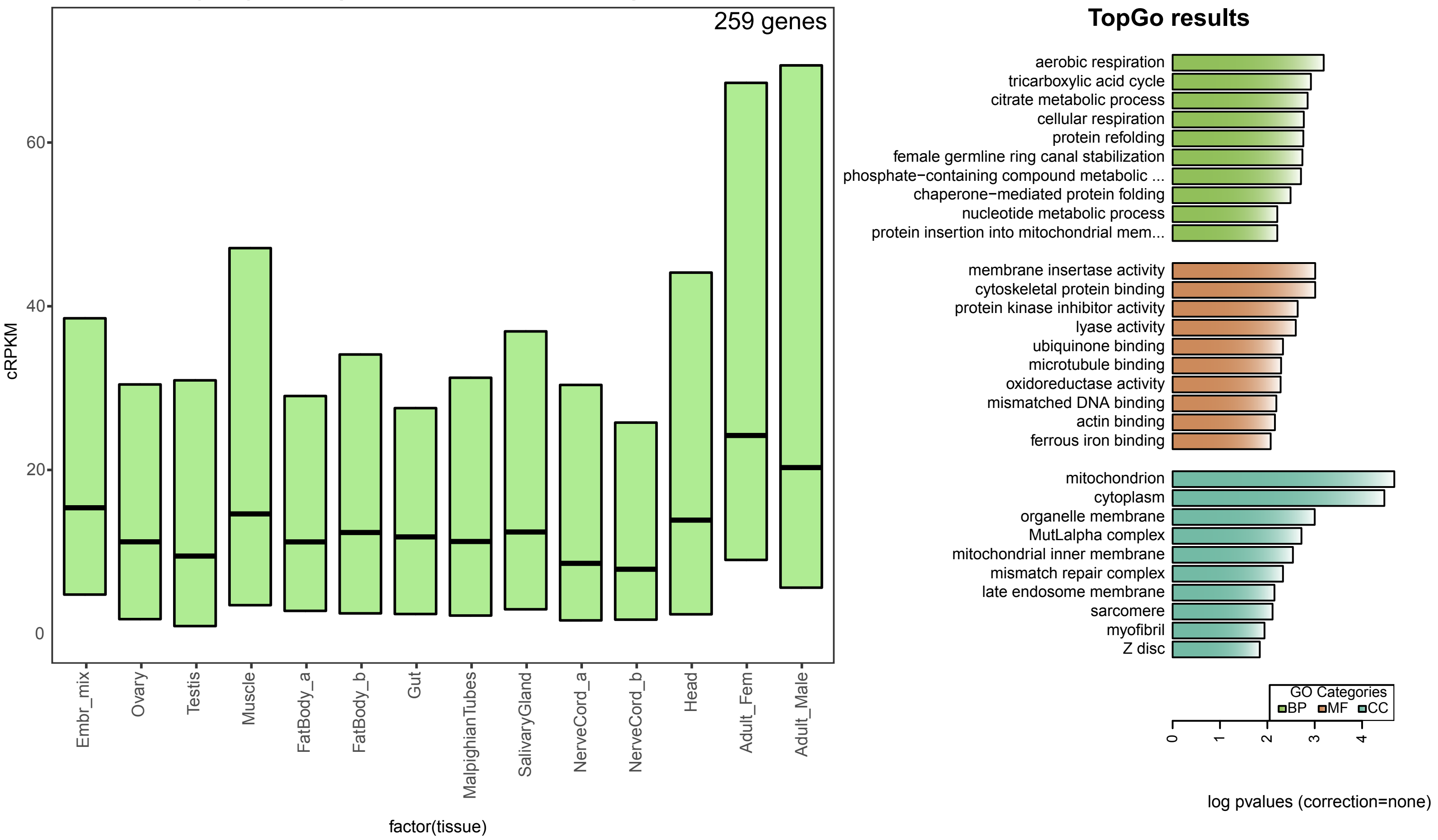

S. maritima modules

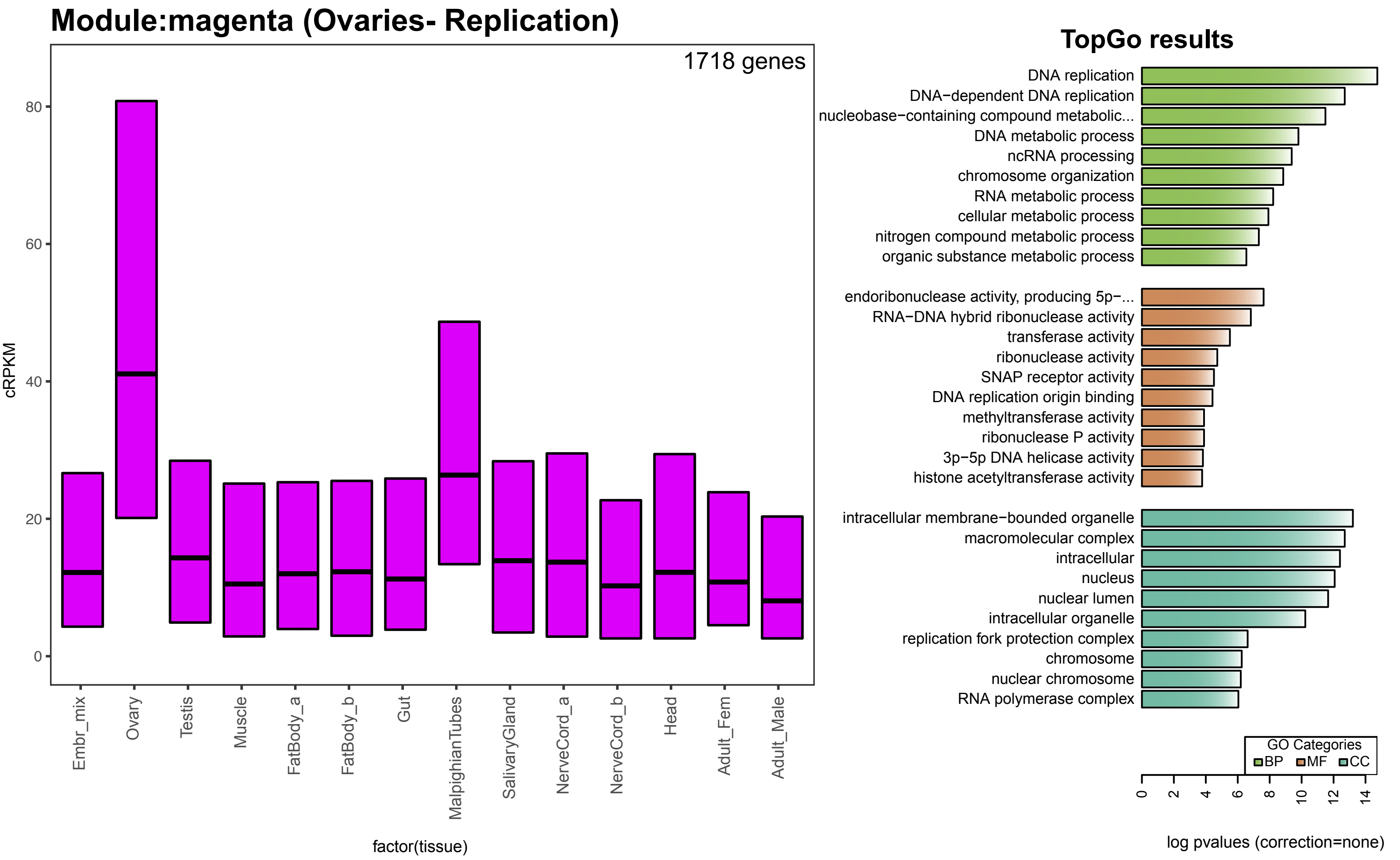

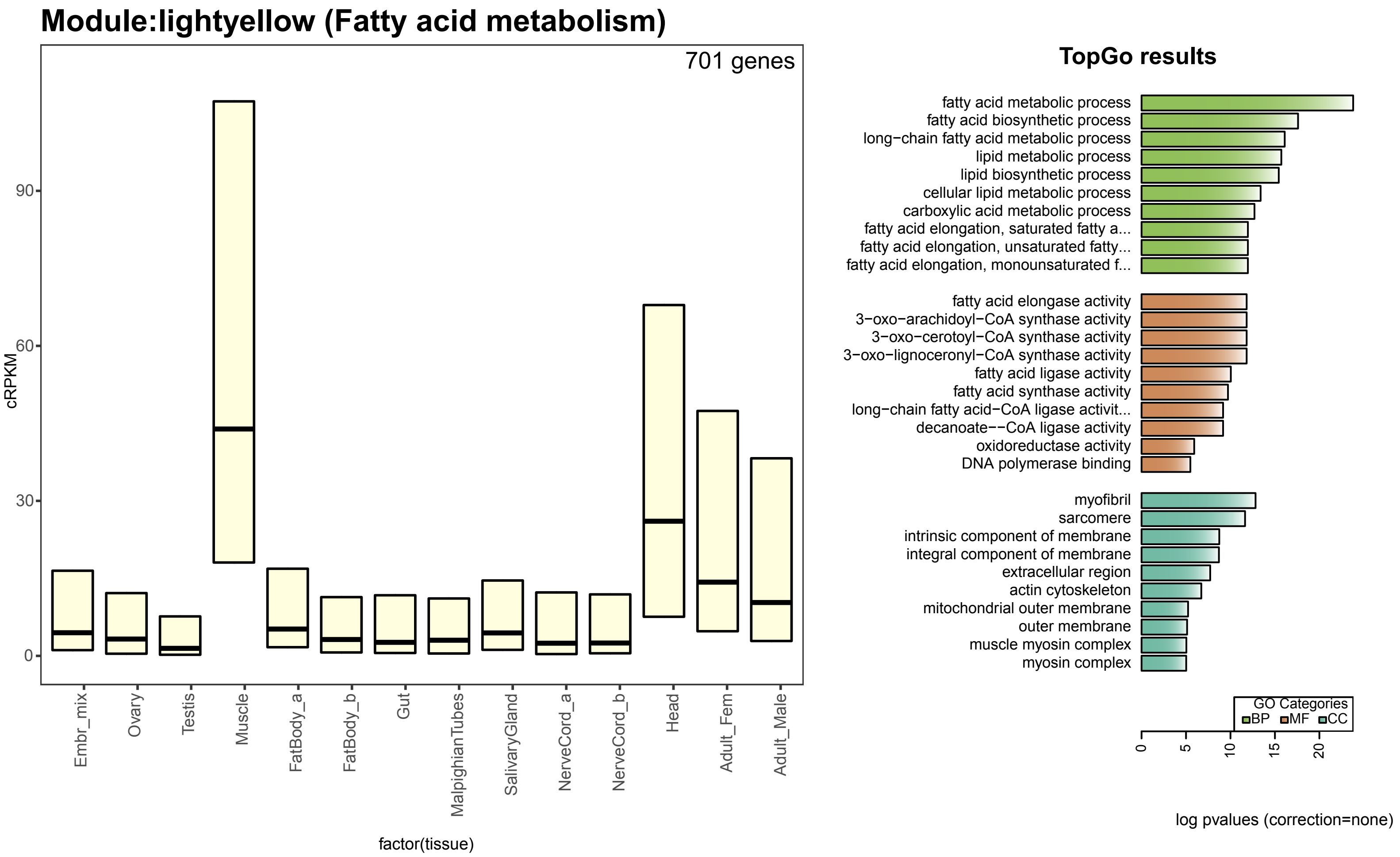

S. maritima modules

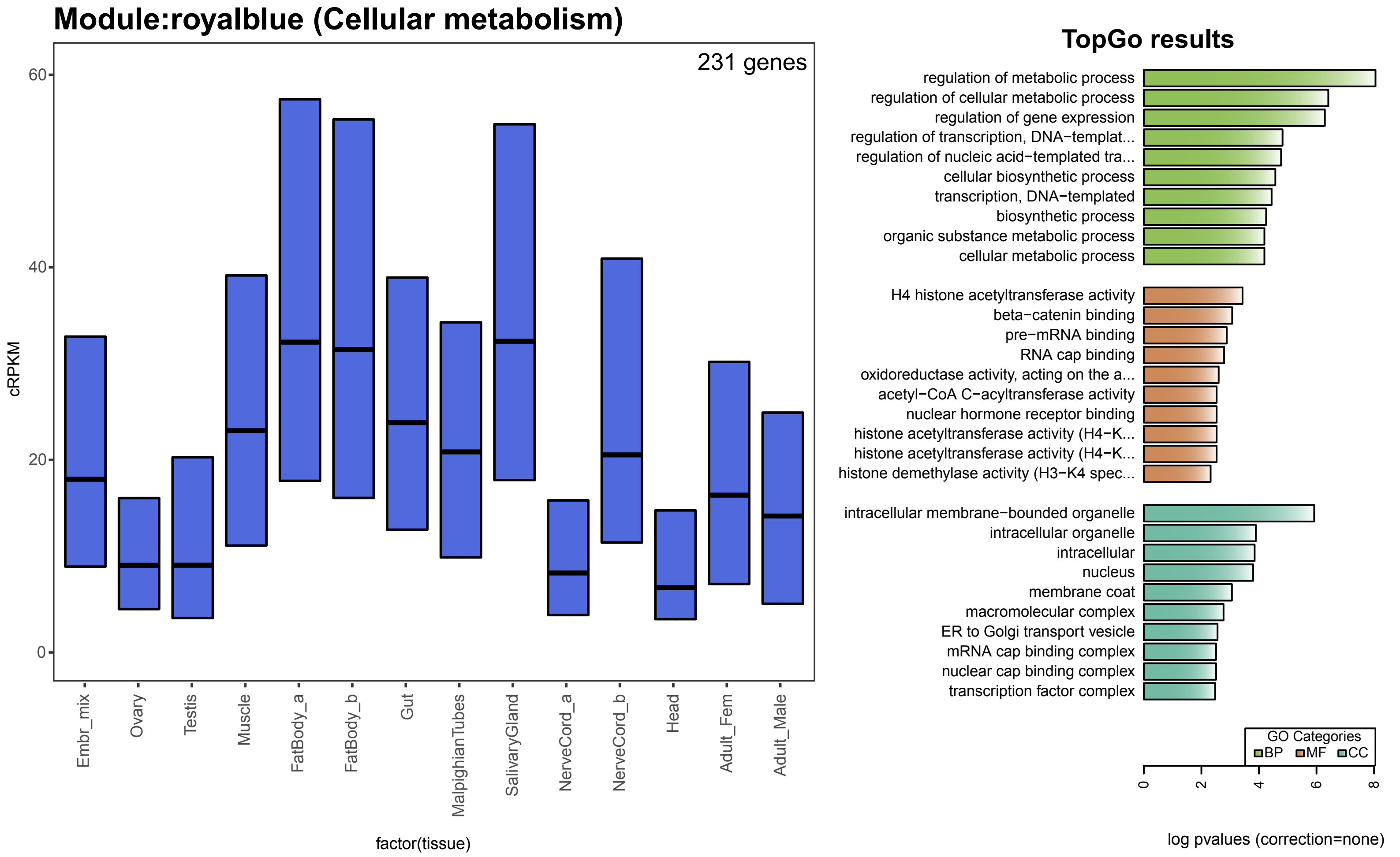

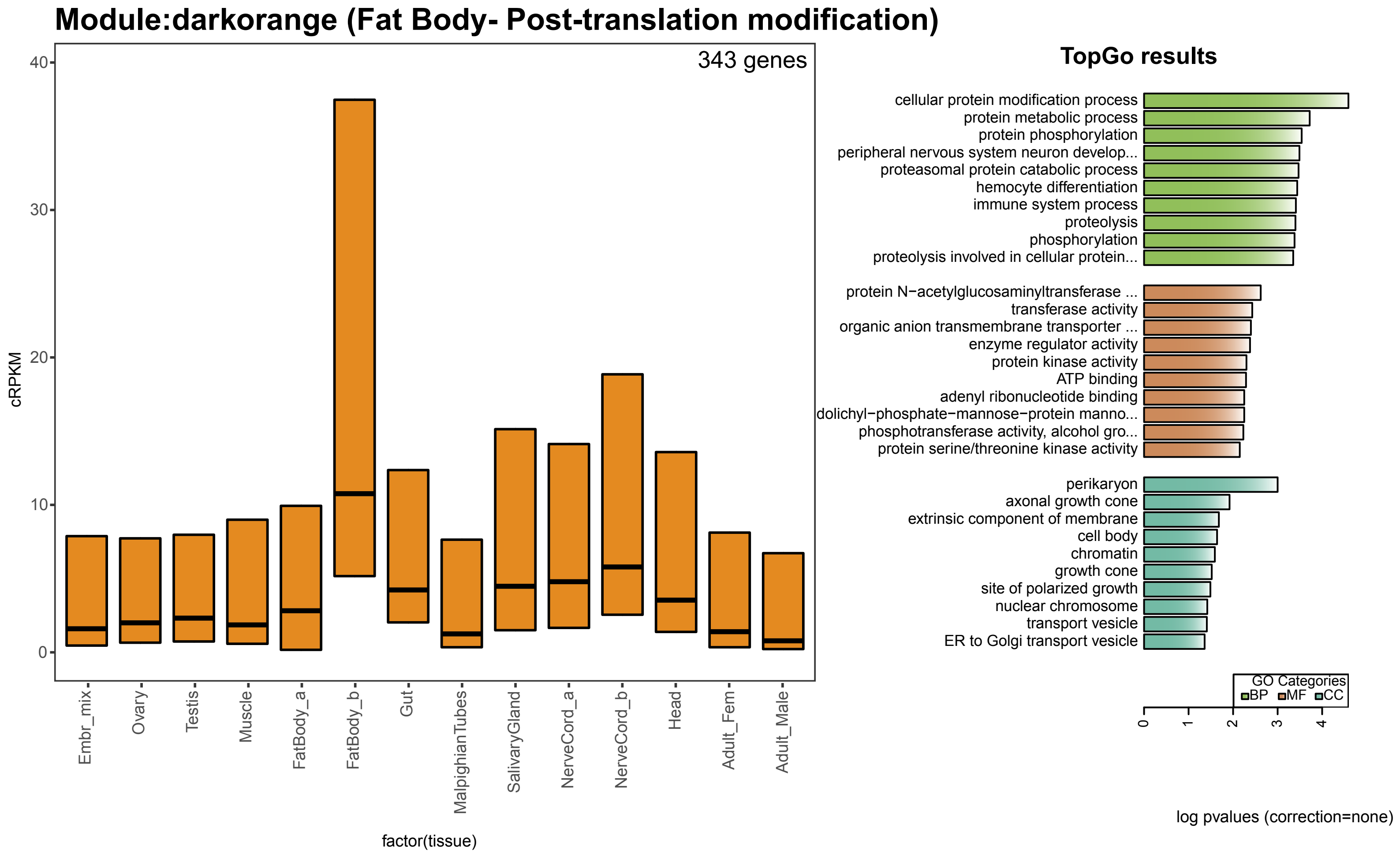

S. maritima modules

Module:brown (Embryogenesis)

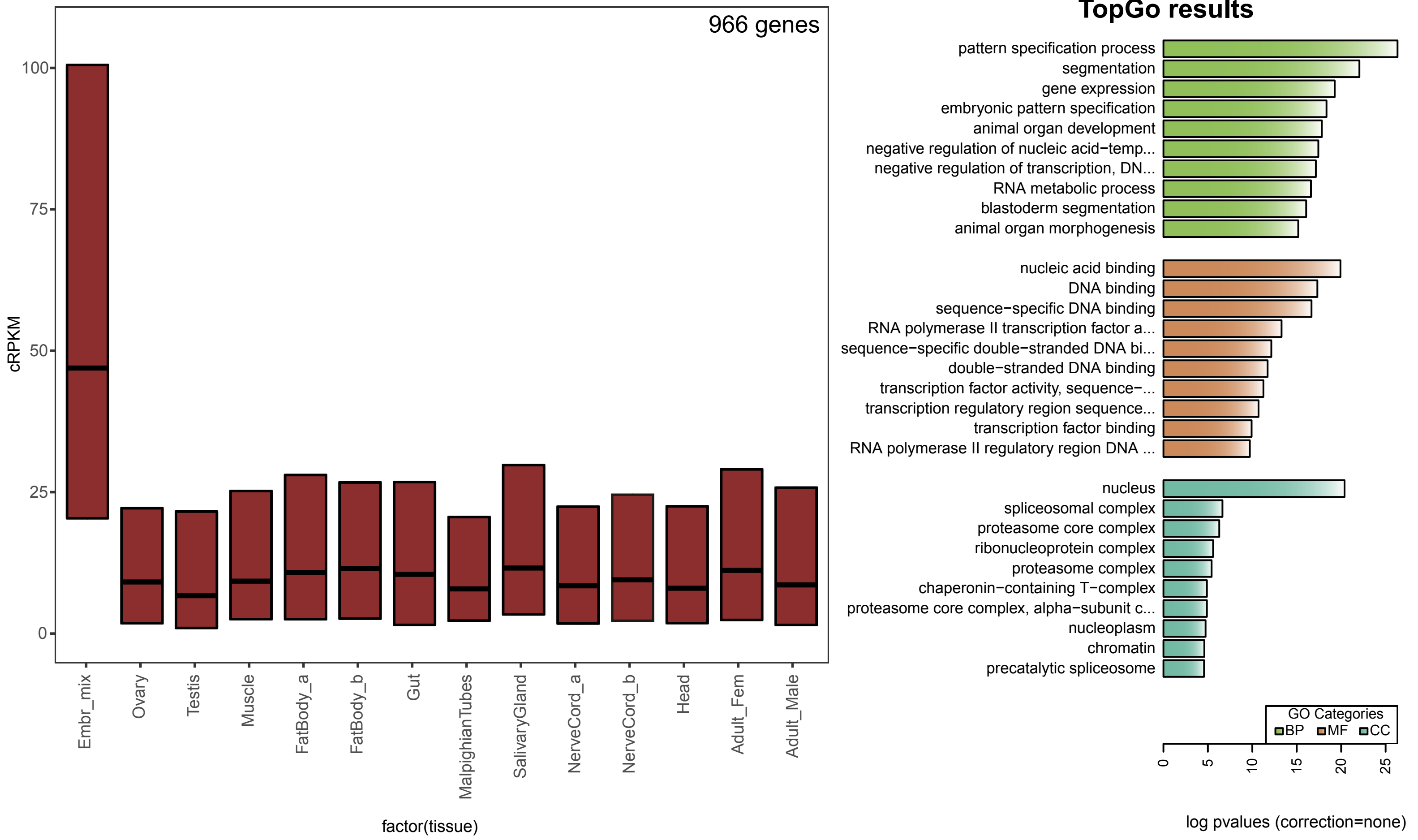

Module:turquoise (Testis- Cilium)

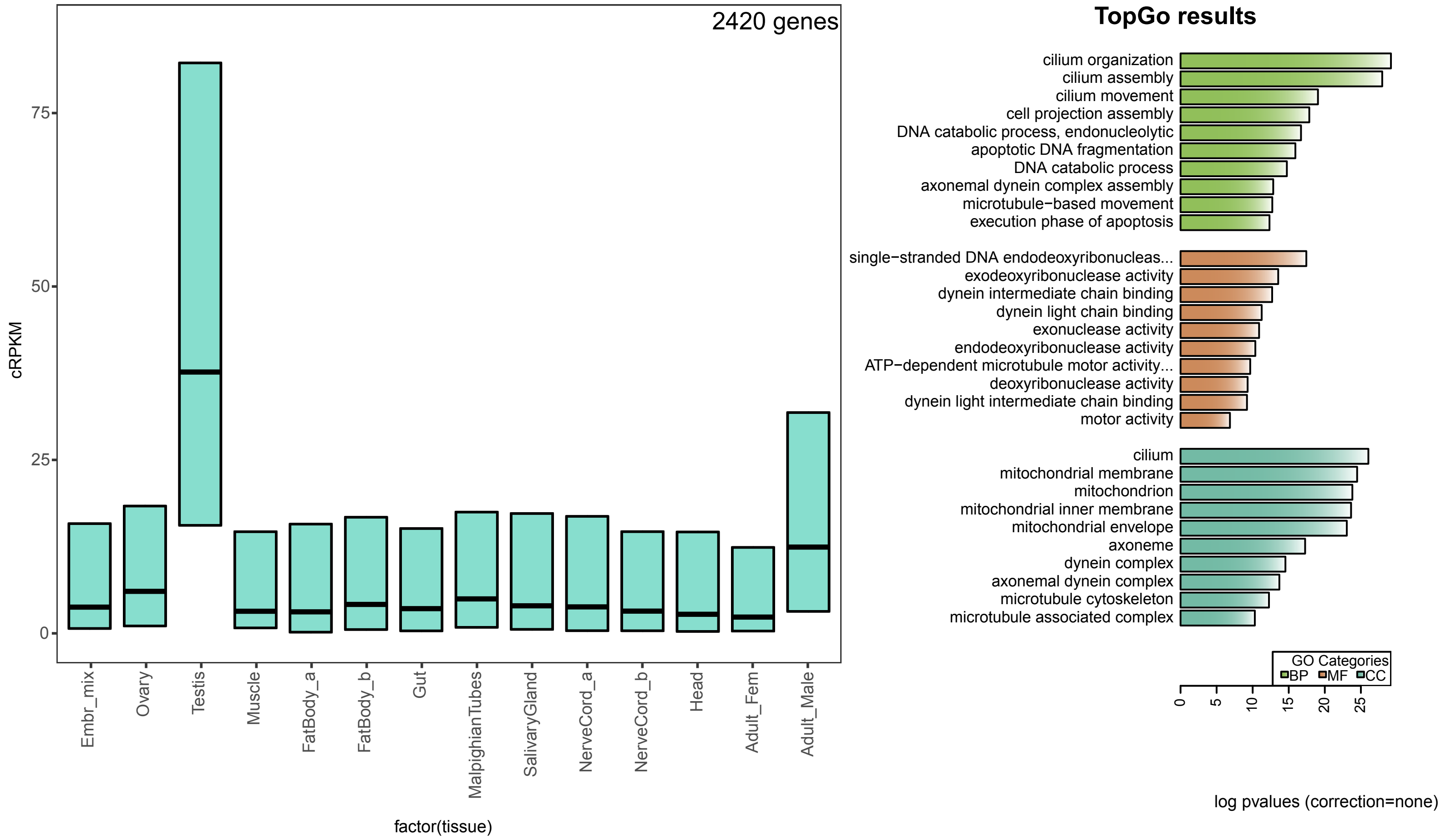

S. maritima modules

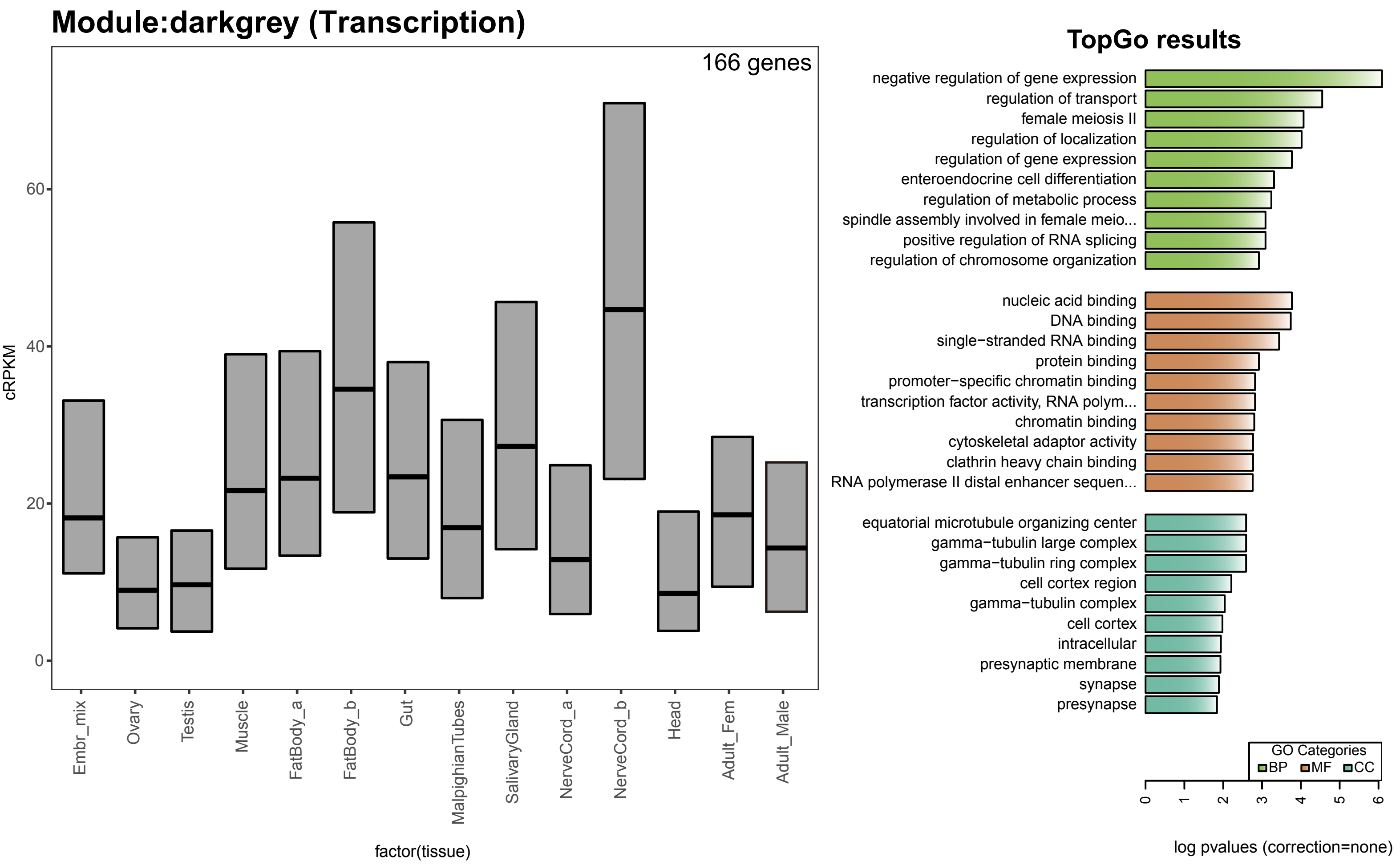

Supplement: Supplementary file 5 — Supplementary Data 1 [file 41467_2020_16284_MOESM5_ESM.pdf]
